# Ligand and Gold(I) Fluorescein–AIEgens as Photosensitizers in Solution and Doped Polymers

**DOI:** 10.1021/acs.inorgchem.3c00197

**Published:** 2023-05-04

**Authors:** Andrea Pinto, Alejandro Llanos, Rosa M. Gomila, Antonio Frontera, Laura Rodríguez

**Affiliations:** † Departament de Química Inorgànica i Orgànica, Secció de Química Inorgànica, Universitat de Barcelona, Martí i Franquès 1−11, 08028 Barcelona, Spain; ‡ Institut de Nanociència i Nanotecnologia (IN2UB), Universitat de Barcelona, 08028 Barcelona, Spain; § Departament de Química, Universitat de les Illes Balears, 07071 Palma de Mallorca, Spain

## Abstract

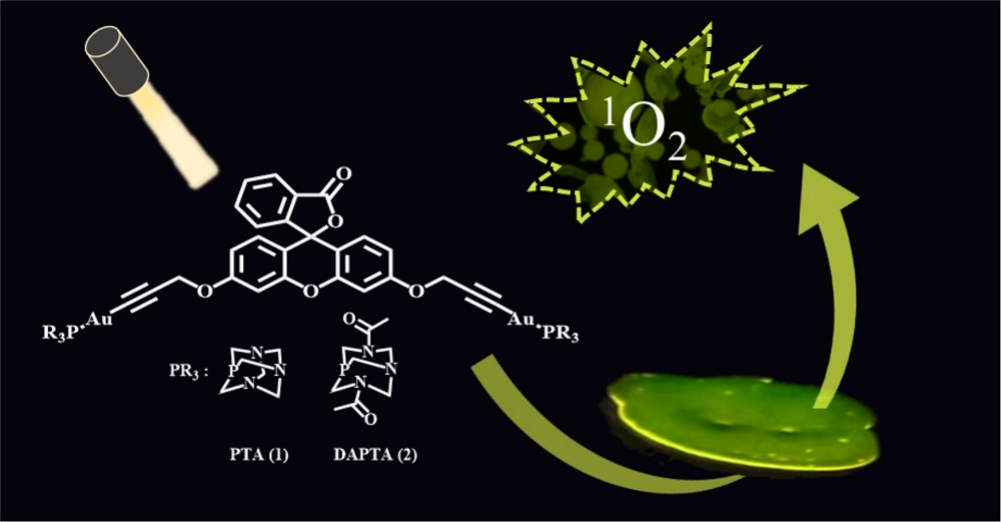

The synthesis of fluorescein propargyl diether (**L**) and two different dinuclear gold(I) derivatives containing a water-soluble phosphane [1,3,5-triaza-7-phosphatricyclo[3.3.1.13.7]decane (PTA) for complex **1** and 3,7-diacetyl-1,3,7-triaza-5-phosphabicyclo[3.3.1]nonane (DAPTA) for complex **2**] has been successfully performed. All compounds display intrinsic emission from fluorescein, being less intense for gold(I) complexes due to the heavy-atom effect. All compounds aggregate in acetonitrile/water mixtures with the formation of larger aggregates for those samples containing more water content, as evidenced by dynamic light scattering and small-angle X-ray scattering experiments, in agreement with the absorption and emission data. The emission of the samples increases when they are used to obtain luminescent materials with four different organic matrices [poly(methyl methacrylate, polystyrene (PS), cellulose, and Zeonex]. The compounds display very high values of singlet oxygen (^1^O_2_) production in dichloromethane. Singlet oxygen production was also evaluated in the doped matrices, being the highest in PS and with an exciting increase on PS microspheres. Density functional theory (BP86-D3) and GFN2-xTB calculations were used to model the assembly of **L** and complexes **1** and **2** with the different organic matrices and rationalize the experimental findings based on the geometries, molecular electrostatic potential surfaces, and complementarity and HOMO–LUMO gaps.

## Abstract 2

Ligand and gold(I) fluorescein−AIEgens have been studied as photosensitizers in solution and doped polymers. Thanks to the functionalization of a fluorescein molecule with allyl and gold(I) moieties, the aggregation-induced emission (AIE) mechanism was possible, avoiding the aggregation-caused quenching seen before for other fluorescein derivatives. Importantly, the compounds display very high values of singlet oxygen (^1^O_2_) production in dichloromethane. Interestingly, the doped matrices with fluoresecein derivatives show high singlet oxygen production in polystyrene (PS) and with an exciting increase on PS microspheres.

## Introduction

Fluorescein is a xanthene dye widely used in industry. It presents excellent photophysical properties with high quantum yield values in aqueous solution, and it is one of the most well-known probes used as sensors for biomolecules, with very low limits of detection, fast response, and the capability of affording high spatial resolution via microscopic imaging.^1^ They have also been used as highly responsive chemosensors, fluorescent markers, singlet oxygen (^1^O_2_) producers, and biolabels among other possible applications.^2−6^

The use of fluorescein in fluorescent materials is scarcely explored because its emission is almost completely quenched in aggregated form (concentrated solutions or powder). This is due to the phenomenon called aggregation-caused quenching (ACQ).^7^ Under these conditions, fluorescein could only be used under diluted concentrations and their applications in areas such as fluorescent materials would be reduced. These types of materials are relevant in very promising research fields such as optoelectronic devices, sensors, fluorescent brighteners, organic light-emitting diodes (OLEDs), or laser dyes among others.^8−13^

Aggregation-induced emission (AIE) is the opposite phenomenon where molecules become emissive upon aggregation. There are several proposed mechanisms that explain the AIE phenomenon, such as restriction of intramolecular motions, restriction of intramolecular rotation, and restriction of intramolecular vibrations.^14^ Overcoming ACQ with AIE mechanisms will open the possibility of using ACQ fluorophores in the previously mentioned research fields such as OLEDs or fluorescent materials.^11,14−16^

Hence, the investigation of possible mechanisms that would avoid ACQ in highly fluorescent probes such as fluorescence is of great importance. Feng et al. accidentally discovered strong solid-state emission in conventional fluorescein by introducing minimal modifications on its structure.^2^ This was observed by functionalization of this molecule with allyl moieties, precluding face-to-face stacking and, thus, hindering the resulting quenching effect due to aggregation.^17,18^ The resulting molecules present protected hydroxyl groups, thus avoiding efficient face-to-face π–π stacking interactions in their crystalline states because of the benzoic ester groups and their resulting steric spatial repulsion together with a staggered arrangement of the xanthenone moiety planes. This steric hindrance results in an opening of the radiative decay channels considering a nonplanar arrangement of these twisted molecules in the solid state.^2,19,20^

Hence, it seems clear that a powerful approach to achieving the ACQ-to-AIE transformation of fluorescein deserves their investigation for the development of new AIEgens and solid luminescent materials. It must be considered that the use of AIEgens as luminescent materials would be much more relevant, with the luminophores introduced in the polymer matrices having the ability to easily form films and be morphologically and thermally stable, which is a great advantage within the optoelectronic industry.

Taking this into consideration, we designed and synthesized herein a fluorescein derivative containing two propargyloxy groups as pending arms in order to block the planarity of the molecule and use it as a AIE molecule. This ligand has also been coordinated to two gold phosphane moieties in order to compare the resulting photophysical properties, and they have also been explored as luminescent probes and materials and singlet oxygen photosensitizers.

## Results and Discussion

### Synthesis and Characterization

**Scheme 1:**
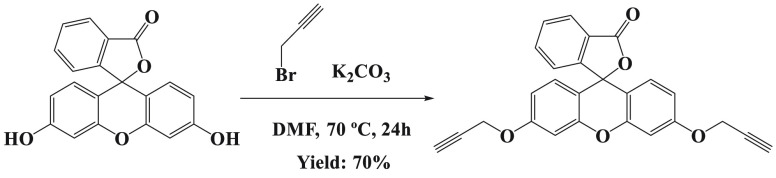
Synthesis of Fluorescein Propargyl Diether (**L**)

**Scheme 2:**
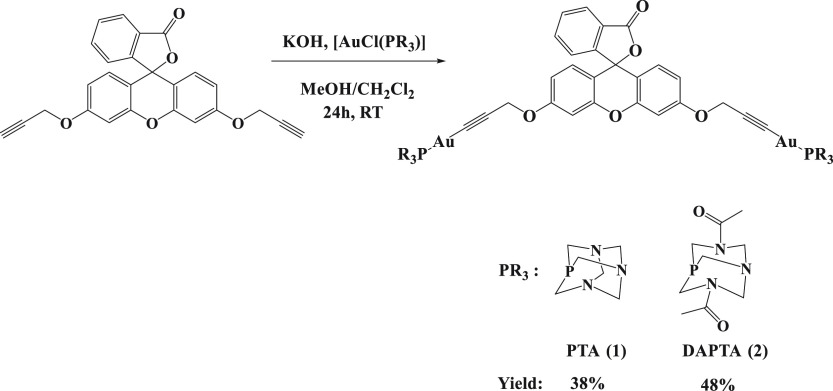
Synthesis of Gold(I) Fluorescein Derivatives **1** and **2**

Two dinuclear gold(I) complexes containing a fluorescein chromophore and two water-soluble phosphanes [1,3,5-triaza-7-phosphatricyclo[3.3.1.13.7]decane (PTA) for compound **1** and 3,7-diacetyl-1,3,7-triaza-5-phosphabicyclo[3.3.1]nonane (DAPTA) for compound **2**] at the second coordination position were synthesized using the procedure outlined in Schemes 1 and 2. The initial step in obtaining the gold(I) fluorescein derivatives is synthesis of the organic alkynyl ligand (**L**). Fluorescein propargylation was performed using the previously stated method, and a comparison of the previous spectroscopic data proved that it was properly formed (Scheme 1).^21^

The gold(I) fluorescein derivatives **1** and **2** were then prepared after deprotonating the terminal alkynyl protons of **L** using a KOH solution in methanol followed by the addition of 2 equiv of [AuCl(PR_3_)] (PR_3_ = PTA or DAPTA) complexes dissolved in dichloromethane (Scheme 2).

Complexes **1** and **2** were characterized by ^1^H and ^31^P NMR and IR spectroscopy and electrospray ionization mass spectrometry [ESI(+)-MS]. The absence of the terminal alkynyl proton in IR and ^1^H NMR spectra evidenced the correct formation of the complexes. The phosphane protons exhibit the typical patterns of PTA and DAPTA. A single peak ca. 10 ppm downfield-shifted with respect to the [AuCl(PR_3_)] precursors has been observed in ^31^P{^1^H} NMR spectra, as was found for other similar compounds (Figures S1–S4).^22−24^ ESI(+)-MS spectra of **1** and **2** show the [M + H]^+^ monoprotonated species (Figures S7 and S8).

### Photophysical Characterization

**Figure 1:**
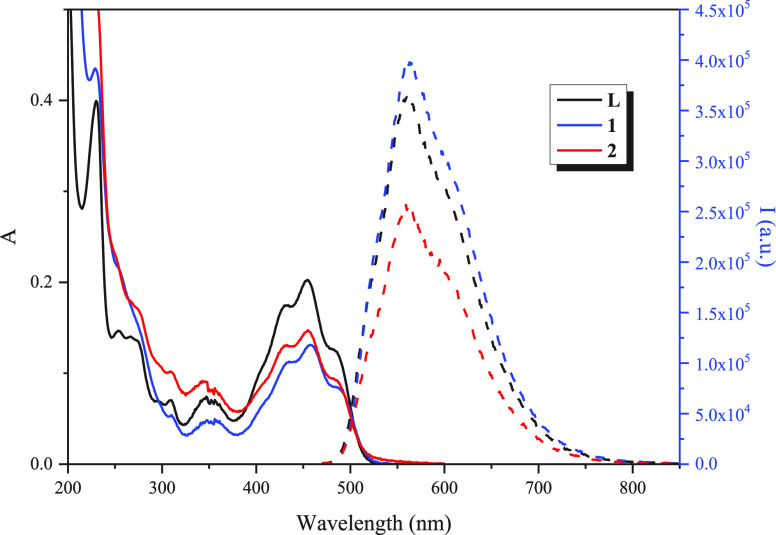
Absorption (solid lines) and emission (dashed lines; λ_exc_ = 450 nm) spectra of **L** and gold(I) complexes **1** and **2** in ACN.

The absorption and emission spectra of the free ligand **L** and the gold(I) complexes were recorded at room temperature in 10^–5^ M acetonitrile (ACN) solutions. The obtained data are tabulated in Table 1 and shown in Figure 1.

**Table 1:** Electronic Absorption and Emission Data, Quantum Yields (Φ_fl_), and Lifetimes (τ_fl_) of **L** and Complexes **1** and **2** (λ_exc_ = 450 nm)

compound	absorption λ_max_ (ε cm^–1^ M^–1^)	emission	Φ_fl_	τ_fl_ (ns)
**L**	235 (38000), 355 (6600), 456 (20132)	551	0.05	0.31, 5.06
**1**	226 (42856), 354 (4471), 463 (12546)	564	0.04	0.69, 2.96
**2**	230 (51875), 351 (8000), 457 (14559)	565	0.04	0.5, 0.96

Gold(I) complex absorption spectra display the same pattern as ligand **L** with a slight red shift on the lowest energy band (around 5 nm) in the case of **1**. This band can be assigned to the π–π* transition of fluorescein based on previous results^25^ and corroborated by density functional theory (DFT) calculations (Figure S9). Moreover, the shape and location of the electron density in the highest occupied molecular orbital (HOMO)–lowest unoccupied molecular orbital (LUMO) do not change in compounds **1** and **2** with respect to ligand **L**.

A similar trend was observed in the emission spectra after the samples were excited at the lowest energy absorption band, with the gold(I) compounds showing a small red shift of ca. 15 nm, which may be attributed to an increase in the conjugation by combining the π system of the ligand with the metal d orbital.^26^ The broadening shape of the emission band must be ascribed to the π*–n/π*−π transitions.^25^

Fluorescence quantum yields (Φ_fl_) and lifetimes (τ_fl_) were measured for all compounds in ACN (Table 1), and the recorded values are below 1%. These low values agree with the presence of a nonconjugated π bond in the lactone conformation in the fluorescein unit (neutral form). All of the compounds’ fluorescence decays were fitted to a double exponential, suggesting the presence of two distinct species in the excited state, which are attributed to two potential different conformations on the lactone ring.^27^

### Aggregation Studies

**Figure 2:**
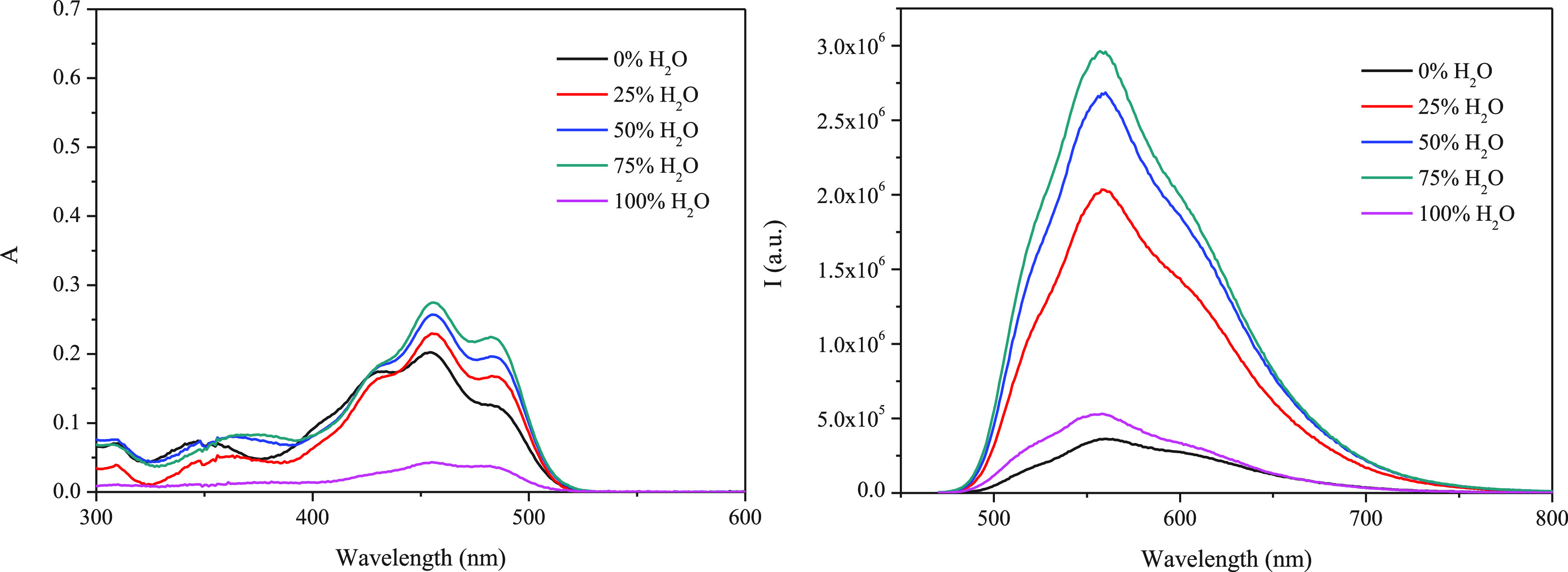
Absorption (left) and emission (right) spectra of **L** in ACN/water mixtures.

Fluorescein’s transformation from a ACQ molecule to an AIE molecule is, as stated above, an exciting research area that will certainly expand the applications of this fluorophore, which is scarcely explored. Interestingly, gold(I) complexation is a very useful tool for modulating the resulting luminescent properties of the molecules and plays a direct role in the resulting aggregation and AIE properties.^16,23,28^ This behavior is enhanced in solvent (herein ACN)/water mixtures, where water acts as a nonsolvent and consequently promotes the aggregation process. It is usually governed by the establishment of several types of noncovalent interactions, such as hydrogen bonds, π–π stacking, and aurophilic contacts.^23,29,30^ Having this in mind, absorption and emission spectra of our compounds were recorded in ACN/water mixtures with increasing water content. A red shift of the lowest absorption band was observed in the absorption spectra with increasing water contents, which is ascribed to the formation of *J* aggregates (Figures 2, left, and S10 and S11). Interestingly, the emission intensity increases in agreement with AIE behavior. The presence of lactone conformation avoids the possibility of forming close face-to-face π–π stacking interactions, leading to the marked ACQ effect of fluorescein. Most importantly, the addition of the propargyl diether group introduces steric spatial repulsion and helps the AIE phenomenon. The absorption and emission of all compounds decrease in pure water solutions probably due to the formation of small insoluble aggregates that cannot be observed by the naked eye.

**Figure 3:**
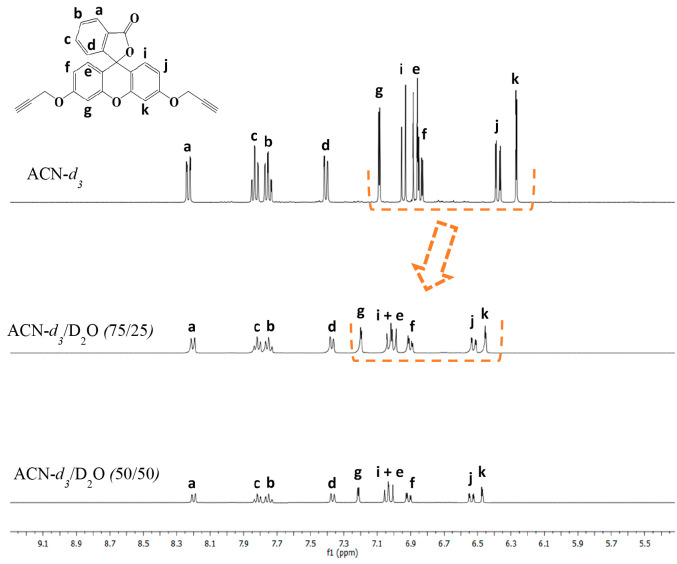
^1^H NMR spectra of **L** in ACN-*d*_3_/D_2_O mixtures.

^1^H NMR experiments were performed in order to gain insight into the aggregation process in the presence of water (Figures 3 and S12). The ^1^H NMR spectrum of **L** in pure ACN-*d*_3_ showed sharp and well-resolved signals, in agreement with the presence of discrete species. The subsequent addition of water induces a shift in some of the proton signals of **L** together with a broadening and a decrease of the aromatic signals. The proton signals of xanthene are downfield-shifted probably due to CH−π and hydrophobic interactions.^31,32^ The broadening and decrease of the aromatic signals suggest the presence of aggregates where the aromatic rings are involved. Similar trends were observed for the gold(I) complexes (Figure S8). In these cases, signals from the aromatic unit’s magnetic relaxation were less efficient than relaxation of the phosphane unit, suggesting that the aromatic unit could be more restricted than the phosphane unit.^23^

**Figure 4:**
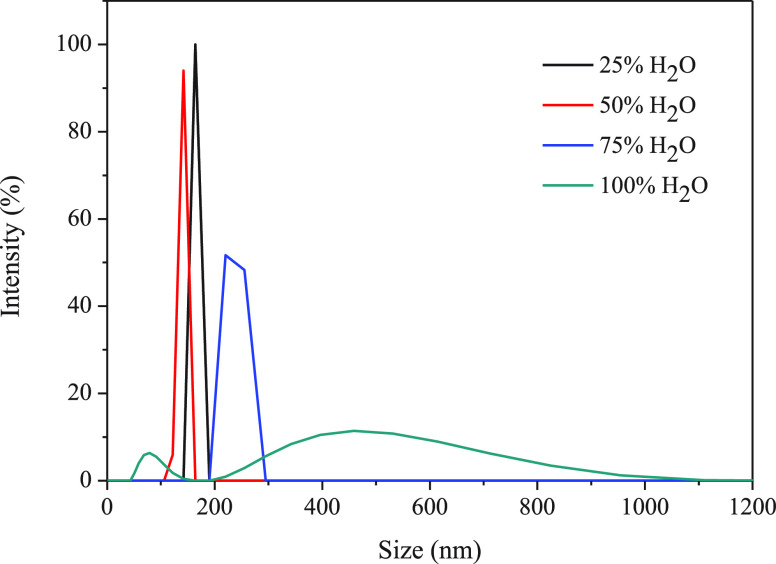
Size distribution obtained by DLS for **L** at different water/ACN ratios.

Dynamic light scattering (DLS) and small-angle X-ray scattering (SAXS) experiments were performed in order to corroborate the presence of aggregates in solution. The measurements were performed with 10^–5^ M solutions of **L** and gold(I) complexes **1** and **2** dissolved in analogous ACN/water mixtures. The key role of water in the formation of aggregates is first observed by DLS data where no aggregates were detected in ACN (100%) solutions. The size of the resulting aggregates increases with larger water contents, and a broader distribution is also observed, giving up to 500 nm supramolecular assemblies (Figures 4 and S13 and S14).

**Figure 5:**
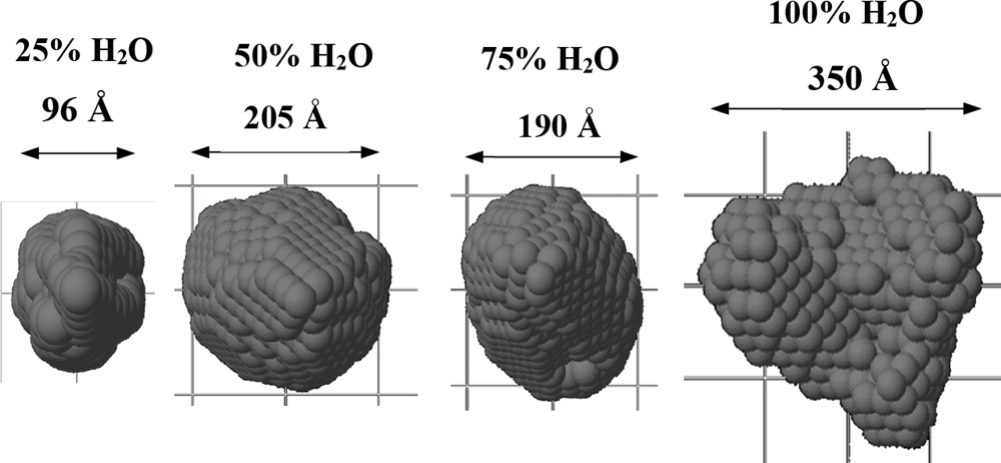
*DAMMIN* low-resolution structures reconstructed from the SAXS patterns for **L** at different water/ACN ratios.

On the other hand, SAXS studies allow us to analyze the shape and size of the aggregates in an early stage. The measurements were performed with 10^–5^ M solutions of **L** and gold(I) complexes **1** and **2** dissolved in analogous ACN/water mixtures. The *DAMMIN* program was used to reconstruct the low-resolution structures from the scattering patterns^33^ (Figures 5 and S15 and S16).

The global information retrieved from scattering experiments indicates that the aggregates are already formed at the very early stages, with the formation of small assemblies of a few angstoms that merge to larger assemblies of hundreds of nanometers in ACN/water mixtures. These results also agree with the photophysical experiments. Interestingly, gold(I) complexes display larger aggregates than the corresponding ligand **L** probably due to an increase of steric hindrance when the gold phosphane moiety is incorporated and to the presence of gold(I) as an additional point to potentiate intermolecular contacts.

### Hybrid Materials Doped with Fluorescein Compounds

**Figure 6:**
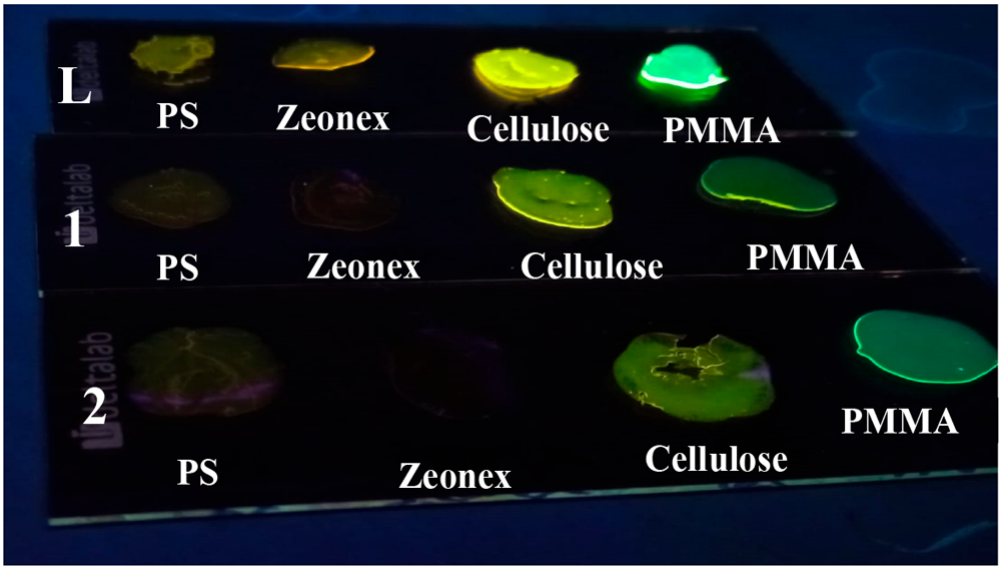
Representation of the color change that displays the different polymer matrices (PS, Zeonex, cellulose, and PMMA) with **L** and gold(I) complexes **1** and **2** at room temperature (λ_exc_ = 365 nm).

The new compounds were dispersed in four different polymeric matrices of different nature [cellulose, poly(methyl methacrylate) (PMMA), polystyrene (PS), and the cyclic olefin copolymer Zeonex) in an attempt to see how the photophysical properties are being affected. Two different trends can be observed. While the more apolar PS- and Zeonex-doped matrices display a yellow/orange emission (10 nm red-shifted in comparison with those of solution), the more polar PMMA- and cellulose-doped materials induce a blue shift in the emission (10 nm) (Figures 6 and S17–S19).

Different concentrations of **L** and gold(I) complexes **1** and **2** doped in the different polymer matrices were tested (0.5%, 3%, and 10%). The emission increases with the concentration except for the highest concentration, where a quenching of the emission was observed. This is in agreement with the aggregation studies, in which at 100% water a quenching in the emission was observed. Therefore, 3% of the luminescent compound was used for further studies.

The quantum yields and lifetimes for different emissive materials are collected in Tables 2 and 3. Generally, a clear increase in the quantum yields and emission lifetimes is observed when the compounds are put into thin films with respect to the more compact solid state. Interestingly, the quantum yields are also larger in the most polar matrices (PMMA and cellulose). In general, the gold(I) compounds display lower quantum yields, meaning that probably we are promoting the triplet excited state through intersystem crossing due to the heavy-atom effect.

**Table 2:** Luminescence Quantum Yields, Φ (%), of **L**, **1**, and **2** in the Solid State and in Matrices of Cellulose, PMMA, PS, and Zeonex

	Φ_fl_				
compound	solid	cellulose	PMMA	PS	Zeonex
**L**	0.5	23.0	29	7.6	2.4
**1**	0.3	25.6	9.3	8.2	1.2
**2**	0.2	11.6	14.6	8.1	0.7

**Table 3:** Lifetimes (ns) of **L**, **1**, and **2** in the Solid State and in Matrices of Cellulose, PMMA, PS, and Zeonex

	τ_fl_				
compound	solid	cellulose	PMMA	PS	Zeonex
**L**	0.91, 2.11	1.99, 4,06	2.40, 4.22	1.44, 6.62	0.83, 3.33
**1**	0.50, 3.45	1.55, 4.42	1.90, 4.29	1.17, 3.92	0.48, 3.53
**2**	0.43, 3.19	2.04, 4,07	2.54, 4.33	1.19, 3.83	0.27, 3.57

**Figure 7:**
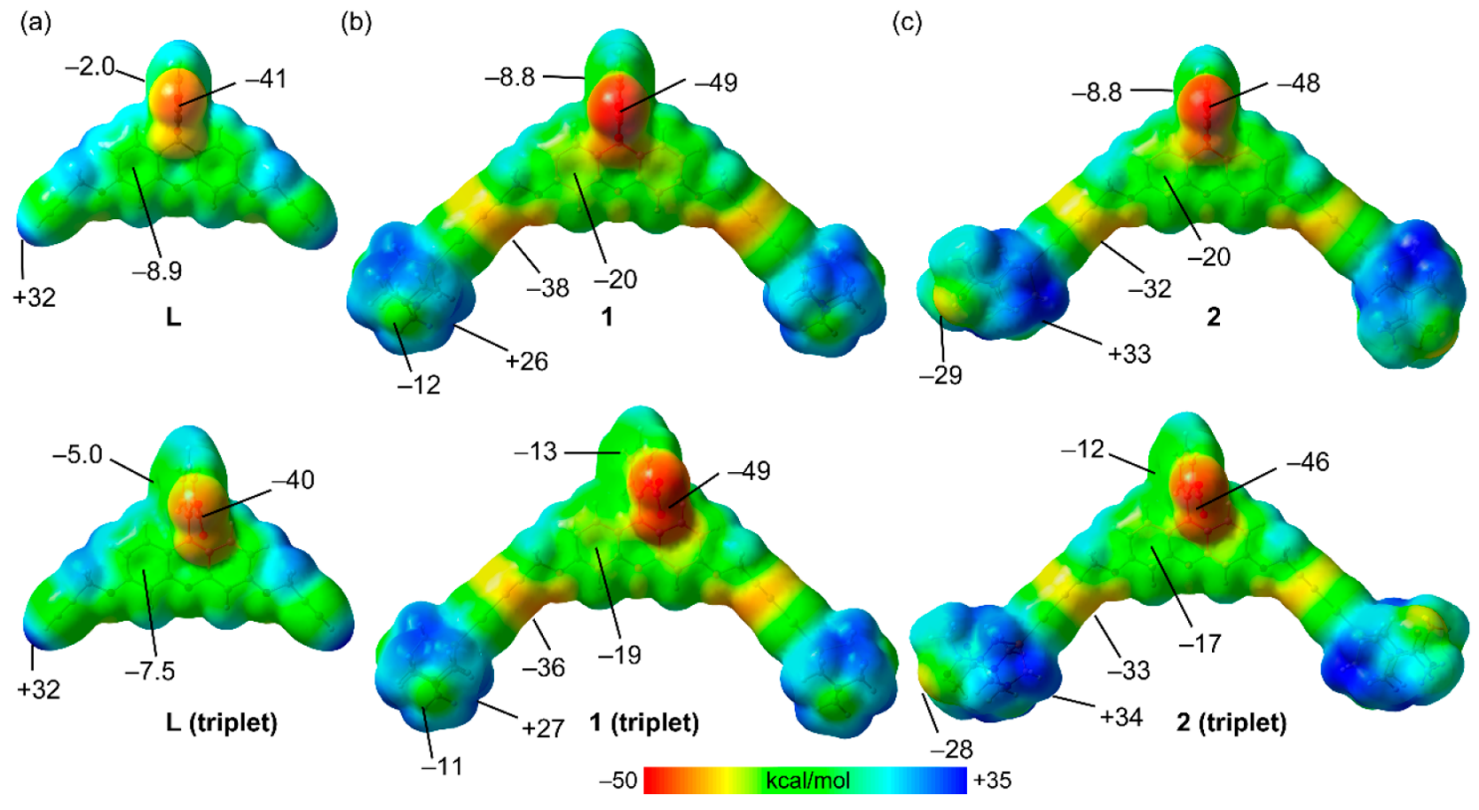
MEP surfaces of the singlet and triplet geometries of the ligand (a) and complexes **1** (b) and **2** (c). The MEP values are given in kilocalories per mole at the RI-BP86-D3/def2-TZVP level of theory.

**Figure 8:**
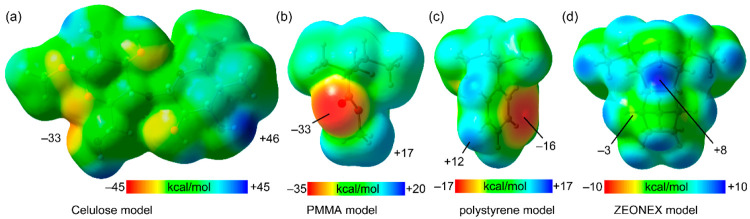
MEP surfaces of monomeric models of cellulose (a), PMMA (b), PS (c), and Zeonex (d). The MEP values are given in kilocalories per mole.

DFT/GNB-xTB calculations (see Theoretical Methods) have been performed to study the interaction of the ligand and compounds **1** and **2** with different matrices and to analyze the effect of the matrices on the HOMO–LUMO gaps. As a first approximation, we have computed the molecular electrostatic potential (MEP) surfaces of compounds **1** and **2** and **L** in their singlet and triplet states and those of the matrices (using a monomeric fragment) to analyze their supramolecular complementarity. The MEP surfaces of **L** and complexes **1** and **2** are given in Figure 8, evidencing that the gold(I) complexes are more basic than the ligand. That is, the MEP values are more negative at the lactone with respect to the ligand, and gold(I) also confers an additional region of negative potential. The most positive regions are located at the ethynyl hydrogen atoms in the ligand (+32 kcal/mol) and at the hydrogen atoms of the phosphine ligands in compounds **1** and **2**. The MEP surfaces of the fully optimized triplets are given in the lower part of Figure 7. Subtle differences regarding the electron distribution are observed. Basically, the π acidity of the lactone ring increases in the triplet state for all three compounds. Therefore, the ability to establish donor–acceptor interactions is expected to be similar in both the singlet and triplet forms of the ligand and compounds **1** and **2**.

The MEP surfaces of monomeric units of the polymeric matrices have also been computed in order to anticipate the affinity of the matrices to the ligand and gold complexes. The plots are depicted in Figure 8, revealing that cellulose is the matrix that presents larger differences between the MEP minimum (−33 kcal/mol) and maximum (+46 kcal/mol), thus suggesting that this matrix should be the one with stronger interactions with the complexes because it has quite complementary values (minimum of −49 kcal/mol in **1** and maximum of +34 kcal/mol in complex **2**). Moreover, this matrix is also the one that has a better electronic distribution to stabilize the triplet state. MEP surface analysis also reveals that the PMMA monomer exhibits a large MEP minimum (−33 kcal/mol), comparable to cellulose, but a modest MEP maximum (+17 kcal/mol). Taking into consideration the basicity of the complexes in both singlet and triplet states, PMMA is expected to have a lower influence in the absorption/emission parameters of complexes compared to cellulose. This behavior is clearly seen in complex **1**, while no significant differences can be observed between these two matrives in **L** and **2**. Examination of the MEP surfaces also suggests that the other two matrices should form weaker interactions with the ligand and complexes than cellulose and PMMA, especially Zeonex. This agrees well with the smaller increase in the quantum yields observed for PS and Zeonex hybrid materials with respect to the solid materials.

**Figure 9:**
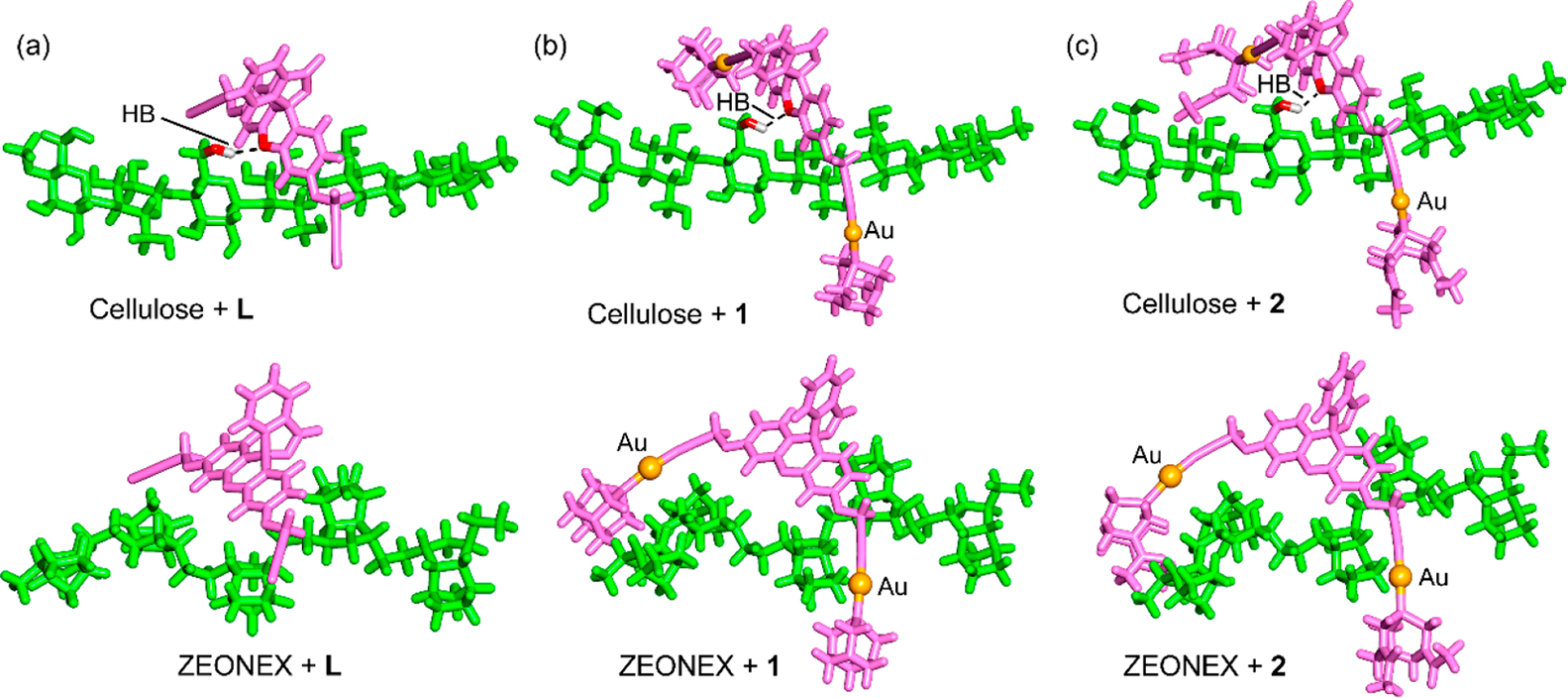
GFN2-xTB geometries of the ligands (a) and compounds **1** (b) and **2** (c) interacting with models of cellulose (upper panel) and Zeonex (lower panel). Hydrogen bonds are represented as dashed lines.

We have also analyzed how the interaction of the ligand and complexes **1** and **2** with the matrices affects the HOMO–LUMO gaps (Table 4). This provides an idea of the influence of the dispersion of the material onto the matrix on the absorption and emission properties of the compounds. It can be observed that cellulose induces on the compounds the largest reduction of the HOMO–LUMO gaps compared to the other matrices, followed by PMMA, in line with the MEP surface analysis. As exemplifying assemblies of polar and nonpolar matrices with the ligand and compounds **1** and **2**, the fully optimized (GFN2-xTB) adducts of cellulose and Zeonex are represented in Figure 9. It can be observed that for cellulose a strong OH···O hydrogen bond fixes the geometry in all three assemblies. For complexes **1** and **2**, the long arms embrace the polymer, incrementing the number of contacts between the matrix and guest. A similar behavior is observed for assemblies of the Zeonex matrix (Figure 9, lower panel). In this case, weak van der Waals contacts are established between the matrix and compounds. These weak interactions likely have a small influence on the luminescence properties of the compounds, as revealed by the small experimental increment of quantum yields for Zeonex summarized in Table 2 in comparison to the solid compounds.

**Table 4:** HOMO–LUMO Gaps in Electronvolts for Assemblies of the Ligand and Compounds **1** and **2** with Pentameric Models of the Matrices^a^

compound and matrix	HOMO–LUMO	compound and matrix	HOMO–LUMO
**L** + cellulose	–2.3767	**L** + PS	–2.4643
**1** + cellulose	–1.9598	**1** + PS	–2.2152
**2** + cellulose	–2.0629	**2** + PS	–2.1581
**L** + PMMA	–2.4203	**L** + zeonex	–2.5200
**1** + PMMA	–2.1214	**1** + zeonex	–2.2628
**2** + PMMA	–2.2037	**2** + zeonex	–2.2053

a Level of theory BP86-D3/def2-TZVP//GFN2-xTB.

### Singlet Oxygen

The population of the triplet state, promoted by gold(I) and its heavy-atom effect, could be one of the explanations for their recorded decrease on the fluorescence quantum yields together with the shorter decay times of their singlet excited states emission compared to the organic precursor. Because phosphorescence was not recorded (Figures S20–S22), the population of the triplet excited state can also be evidenced by the potential of the compounds as singlet oxygen photosensitizers.

The direct measurement of the singlet oxygens’ phosphorescence at 1270 nm has been used to prove its production. To quantify this process, the standard reference 1*H*-phenal-1-one was used (Figure S23). The singlet oxygen quantum yield production values (45–92%; Table 5) obtained are significantly higher compared with other gold(I) complexes found in the literature.^24,34−37^

**Table 5:** Singlet Oxygen Sensitization Quantum Yields, Φ_Δ_, Obtained in Dichloromethane Air-Equilibrated Solutions for the Investigated Compounds (λ_exc_ = 450 nm)

compound	Φ_Δ_
**L**	0.92
**1**	0.45
**2**	0.81

**Figure 10:**
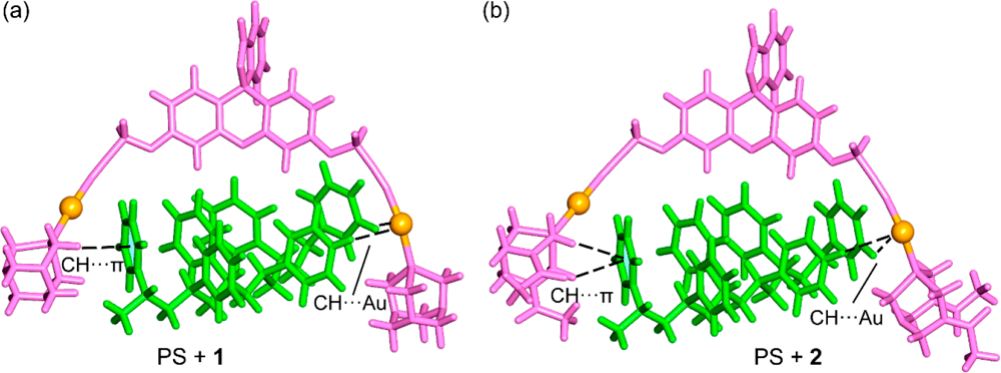
GFN2-xTB geometries of compounds **1** (a) and **2** (b) interacting with models of PS. Noncovalent interactions are represented as dashed lines.

The production of singlet oxygen of the compounds was also studied in the doped polymer matrices, and singlet oxygen production was detected in almost all cases (Figures S24–S26). Interestingly, PS presents the higher-intensity signal of singlet oxygen production for all compounds. This may be attributed to the presence of less aggregates in this polymer matrix than the others. In fact, theoretical calculations reveal that for this matrix a different interaction mode occurs (Figure 10) in comparison with the others described above (Figure 9). That is, the fluorophore is not in contact with the matrix, likely due to the fact that there is not enough space to intercalate the aromatic surface of the fluorophore between the phenyl rings of the matrix. The interaction of compounds **1** and **2** with the matrix basically involves either the positive hydrogen atoms of the coordinated phosphine and the negative aromatic ring of the matrix (CH···π) or the positive aromatic hydrogen atoms of the matrix with the negative gold(I) atoms (CH···Au), as depicted in Figure 9 by dashed lines. Both interactions are in line with the MEP surface analysis (vide supra) of the compounds and matrices.

In order to improve the signal obtained, different concentrations were tested, where it was found that, as expected, the emission intensity of ^1^O_2_ is proportional to the concentration used (Figures S29–S31).

**Figure 11:**
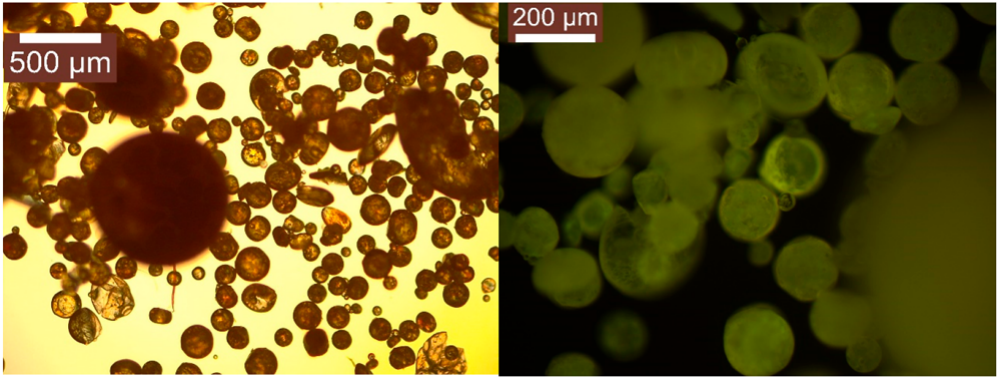
Optical microscopy image of the PS microspheres of **2** under light (left) and UV light (right).

The highest signal recorded in PS encouraged us to synthesize PS microspheres doped with our compounds in order to increase the active surface where the photosensitizer was deposited. In fact, some studies with other polymer matrices have been found in which generation of the singlet oxygen is mainly at the surface.^38^ As can be observed in the corresponding fluorescence microscopy images (Figures 11 and S27 and S28), our compounds are homogeneously distributed along the whole material.

Thus, singlet oxygen measurements were carried out, and higher ^1^O_2_ production was recorded in comparison with their analogues of PS-doped polymer films (Figures S29–S31). This increase can be ascribed to an increase of the surface containing the fluorophore, which is acting as the photosensitizer. Interestingly, this effect is much more favored for gold(I) complexes and especially for the less emissive complex **2** with a 3-fold increase for **1** and a 6-fold increase for **2** of the singlet oxygen emission. These results suggest that these PS-doped microspheres could be used for the photodegradation of organic pollutants in water as a potential application.

## Conclusions

The synthesis of fluorescein derivatives containing substituents that induce steric hindrance has been shown to be an interesting tool to generate fluorescein–AIE samples. In particular, the use of fluorescein propargyl diether and two gold(I) dinuclear compounds has demonstrated an increase of the emission intensity in ACN/water mixtures, in agreement with previous data with organic fluorescein compounds functionalized with allyl moieties. Thus, it has also been demonstrated herein that this is a promising way to preclude the face-to-face stacking well-known to produce ACQ.

The emission of the compounds is very intense in dichloromethane solutions but quite weak in the solid state. Nevertheless, the introduction of the compounds in organic matrices allow us produce highly emissive materials. Model assemblies of the matrices with the ligand and two gold(I) dinuclear compounds have been calculated along with their influence on the HOMO–LUMO gaps, allowing us to rationalize the higher ^1^O_2_ production of the PS-doped matrix. Moreover, MEP surface analysis is a convenient tool to anticipate the complementarity between the fluorescein derivatives and matrices.

The population of the triplet excited state was evidenced by the efficiency of the compounds as singlet oxygen photosensitizers. Interestingly, the preparation of fluorescein-doped materials that act also as photosensitizers was also evaluated, and very relevant results were obtained for mainly PS microspheres, with a gold(I) compound that displayed lower emission intensity.

## Experimental Section

### General Procedures

All manipulations were performed under prepurified N_2_ and using standard Schlenk techniques. All solvents were distilled from the appropriated drying agents. The commercial reagents fluorescein (Aldrich), propargyl bromide (Aldrich, 80%), 1,3,5-triaza-7-phosphatricyclo[3.3.1.13.7]decane (PTA, Aldrich 97%), and 3,7-diacetyl-1,3,7-triaza-5-phosphabicyclo[3.3.1]nonane (DAPTA, Aldrich 97%) were used as received. Literature methods were used for the preparation of [AuCl(PR_3_)] (PR_3_ = PTA^39^ or DAPTA^40^) and fluorescein propargyl diether (**L**).^21^

### Physical Measurements

IR spectra were recorded on a Nicolet FT-IR 520 spectrophotometer. ^1^H NMR [δ (TMS) = 0.0 ppm] spectra were obtained on Varian Mercury 400 and Bruker 400 (Universitat de Barcelona) spectrometers. ESI(+)-MS spectra were recorded on a Fisons VG Quatro spectrometer (Universitat de Barcelona). Absorption spectra were recorded on a Varian Cary 100 Bio UV spectrophotometer and emission spectra on a Horiba Jobin-Yvon SPEX Nanolog spectrofluorimeter (Universitat de Barcelona). Luminescence quantum yields were recorded upon excitation of the samples at 400 nm using an absolute photoluminescence quantum yield spectrometer from Hamamatsu Photonics. Fluorescence lifetimes were measured on a time-correlated single-photon-counting technique using a DeltaPro fluorescence lifetime system from Horiba upon excitation of the sample with a 390 nm nanoLED. DLS data were carried out in a Zetasizer NanoS spectrometer (Universitat de Barcelona). The samples were measured in quartz cuvettes. SAXS experiments were performed at the ALBA Synchrotron on the NCD-SWEET beamline at 12.4 keV, and the sample-to-detector distance was 6.2 m to cover the range of momentum transfer of 0.028 < *q* < 2.56 nm^–1^. The recorded data were collected on a Pilatus3S 1 M detector with a pixel size of 172.0 × 172.0 μm^2^. The exposure time was 30 s. The *q*-axis calibration was obtained using silver behenate.^41^ The program *pyFAI* was used for integration of the SAXS 2D data into 1D data.^42^ The data were then subtracted by the corresponding background using *PRIMUS* software.^43^ The maximum particle dimension *Dmax* and the pair distance distribution *P*(*r*) were determined with *GNOM*.^44^ The low-resolution structure of the aggregates was reconstructed ab initio using the program *DAMM* from the initial portions of the scattering patterns.^33^ Optical microscopy images were acquired on a Leica ICC50 W microscope equipped with a Nikon DXM1200F digital camera.

### Theoretical Methods

The geometries of the matrix models and their complexes with compounds **1** and **2** and ligand **L** were fully optimized using the GFN2-xTB method.^45^ This was developed for calculation of the geometries and noncovalent interaction energies for large molecular systems. The D4 correction model is included in the definition, and thus the method is adequate for studying π-stacking interactions. The initial calculations were performed using the *xTB* program.^46^ Solvent effects were taken into consideration by using the GBSA model.

All DFT calculations were done by means of the *TURBOMOLE 7.0* program.^47^ For the HOMO–LUMO plots and singlet and triplet geometries of the ligand and compounds **1** and **2**, DFT optimizations were performed using the cost-effective BP86-D3/def2-TZVP method.^48−52^ For gold, effective core potentials (ECP-60 scheme)^53^ were used for the inner-shell electrons that also include scalar relativistic effects. The conductor-like solvation model (COSMO)^54^ was used to emulate solvent effects, as implemented in *TURBOMOLE 7.0*. The HOMO–LUMO gaps of the matrices with the compounds were calculated using the GFN2-xTB geometries and single points at the BP86-D3/def2-TZVP level of theory. The Cartesian coordinates of the optimized dimers are given in the Supporting Information.

### Singlet Oxygen Quantum Yields

Room-temperature singlet oxygen emission was detected at 1270 nm with a Horiba Jobin-Yvon SPEX Nanolog spectrofluorimeter (Universitat de Barcelona) using a DSS-IGA020L detector. It was necessary to use a Schott RG 1000 filter to completely remove the first harmonic contribution of the sensitizer emission in the range below 850 nm from the IR signal. The quantum yield of singlet oxygen production was measured directly from phosphorescence at 1270 nm after irradiation of the aerated solutions of the samples. 1*H*-Phenal-1-one in dichloromethane was used as the standard reference, and eq 1 was applied.
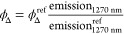
1with ϕ_Δ_^ref^ the singlet oxygen formation quantum yield of the reference compound.^55^

### Preparation of Matrices Doped with **L** and Gold(I) Complexes **1** and **2**

Cellulose, PMMA, PS, and Zeonex (Zeon Corp., Japan) were used as the matrix polymers. The films were made using a drop-casting technique with a combination of dopant and host (cellulose, PMMA, PS, or Zeonex). Polymer solutions were prepared as follows: PMMA (MW 120000, 30% solution in dichloromethane), PS (MW 45000, 35% solution in dichloromethane), cellulose (MW 30000, 20% in acetone), and Zeonex (20% in chloroform). The same volume of a sample solution with a concentration of 20 g/mL was added to a polymer solution. To prevent any thermal annealing, the films were drop-casted onto a quartz substrate at ambient temperature.

### Preparation of PS Microspheres

PS microspheres were prepared through the extraction–evaporation method, as previously reported.^56,57^ Briefly, a solution of PS and the corresponding fluorophore in dichloromethane were mixed with a 1% poly(vinyl alcohol) aqueous solution and stirred for 24 h. The microspheres formed were filtered and dried under air for 48 h.

### Synthesis and Characterization

#### Synthesis of [(C_20_H_10_O_3_)(OCH_2_C≡CAuPTA)_2_] (**1**)

**L** (51 mg, 0.13 mmol) in methanol (5 mL) was combined with KOH (21 mg, 0.57 mmol) in 5 mL of methanol. A dichloromethane solution (10 mL) of [AuCl(PTA)] (112 mg, 0.26 mmol) was added after 30 min of stirring, and the solution was kept at room temperature while being shielded from light by aluminum foil. After 24 h of stirring, the solution was concentrated to ca. 5 mL, and 10 mL of hexane was then added to precipitate an orange solid. The solid was isolated by filtration in 38% yield (53 mg).

^1^H NMR (CDCl_3_): δ 8.21 (d, *J* = 8.0 Hz, 1H, H_a_), 7.72–7.68 (td, *J* = 4.0 and 2.4 Hz, 1H, H_b_), 7.65–7.61 (td, *J* = 4.0 and 2.4 Hz, 1H, H_c_), 7.27 (d, *J* = 8.0 Hz, 1H, H_d_), 7.14 (d, *J* = 1.2 Hz, 1H, H_g_), 6.83 (d, *J* = 12.0 Hz, 1H, H_e_), 6.81 (d, *J* = 8.0 Hz, 1H, H_i_), 6.77 (dd, *J* = 8.0 and 3.0 Hz, 1H, H_f_), 6.51 (dd, *J* = 12 and 3.2 Hz, 1H, H_j_), 6.44 (d, *J* = 3.2 Hz, 1H, H_k_), 4.54–4.44 (AB q, *J* = 12.8 Hz, 6H, H_2_), 4.22 (s, 6H, H_1_). ^31^P{^1^H} NMR (CDCl_3_): δ −50.8 (br). ^13^C NMR (CDCl_3_): δ 185.72, 173.51, 167.21, 162.28, 159.38, 154.46, 134.32, 132.58, 131.20, 130.64, 130.53, 129.73, 120.61, 128.84, 117.82, 115.22, 114.84, 105.41, 101.79, 73.16, 72.30, 57.51, 56.31, 55.80, 54.58, 52.34, 29.72, 20.44, 1.03. IR (KBr, cm^–1^): ν(C–H) 2932, ν(C=O) 1717. ESI(+)-MS: *m*/*z* 1115.178 ([M + H]^+^, calcd *m*/*z* 1115.171).

#### Synthesis of [(C_20_H_10_O_3_)(OCH_2_C≡CAuDAPTA)_2_] (**2**)

Complex **2** was synthesized following the same experimental procedure as that reported for **1** but using [AuCl(DAPTA)] instead of [AuCl(PTA)]. An orange solid was obtained in a yield of 48% (74 mg).

^1^H NMR (CDCl_3_): δ 8.21 (d, *J* = 8.0 Hz, 1H, H_a_), 7.73–7.69 (td, *J* = 4.0 and 1.6 Hz, 1H, H_b_), 7.66–7.62 (td, *J* = 4.0 and 2.4 Hz, 1H, H_c_), 7.27 (d, *J* = 8.0 Hz, 1H, H_d_), 7.14 (d, *J* = 1.6 Hz, 1H, H_g_), 6.84 (d, *J* = 12.0 Hz, 1H, H_e_), 6.80 (d, *J* = 12.0 Hz, 1H, H_i_), 6.75 (dd, *J* = 8.0 and 3.0 Hz, 1H, H_f_), 6.51 (dd, *J* = 12 and 3.2 Hz, 1H, H_j_), 6.44 (d, *J* = 3.2 Hz, 1H, H_k_), 5.75 (d, *J* = 16.0 Hz, 2H, H_4,a_, H_4,a′_), 5.55 (dd, *J* = 16.0 and 4.0 Hz, 2H, H_3,a_, H_3,a′_), 4.93–4.88 (m, 4H, H_1,a_, H_1,a′_, H_5,a_, H_5,a′_), 4.61 (d, *J* = 16.0 Hz, 4H, H_3,b_, H_3,b′_, H_5,b_, H_5,b′_), 4.10 (d, *J* = 16.0 Hz, 2H, H_1,b_, H_1,b′_), 4.02 (d, *J* = 16.0 Hz, 2H, H_4,b_, H_4,b′_), 3.83 (s, 4H, H_2_), 3.63 (s, 4H, H_l_, H_m_), 2.07 (s, 6H, H_6_). ^31^P{^1^H} NMR (CDCl_3_): δ −22.9 (br). ^13^C NMR (CDCl_3_): δ 185.72, 170.03, 169.80, 165.64, 162.47, 154.19, 134.74, 132.70, 131.12, 130.62, 130.21, 129.95, 129.65, 128.73, 117.61, 114.97, 114.36, 105.73, 101.56, 67.34, 62.10, 57.59, 52.47, 49.66, 49.38, 44.63, 39.72, 39.42, 21.60, 21.27. IR (KBr, cm^–1^): ν(C–H) 2920, ν(C=O) 1719. ESI(+)-MS: *m*/*z* 1259.217 ([M + H]^+^, calcd *m*/*z* 1259.213).

## Supporting Information Available

^1^H, ^31^P, and ^13^C NMR spectra for gold(I) complexes, high-resolution MS spectra for gold(I) complexes, HOMO–LUMO plots for all compounds, absorption and emission spectra for all compounds, DLS spectra for gold(I) complexes, *DAMMIN* low-resolution structures for gold(I) complexes, optical microscopy images for all compounds, and Cartesian coordinates for all compounds (PDF)
