# Supplementary material for: Ligand and Gold(I) Fluorescein–AIEgens as Photosensitizers in Solution and Doped Polymers
Source: Inorg Chem. 2023 May 4;62(19):7131–40. doi: 10.1021/acs.inorgchem.3c00197 (PMC10189736; doi:10.1021/acs.inorgchem.3c00197)
Supplement: Supplementary file 1 — ic3c00197_si_001.pdf [file ic3c00197_si_001.pdf]

Ligand and Gold(I) fluorescein-AIEgens as photosensitizers in solution and doped-polymers

Andrea Pinto,[a,b*] Alejandro Llanos,[a] Rosa M. Gomila,[c] Antonio Frontera[c,*] and Laura Rodríguez[a,b]

[a] *Departament de Química Inorgànica i Orgànica, Secció de Química Inorgànica, Universitat de Barcelona, Martí i Franquès 1-11, E-08028 Barcelona, Spain. e-mail:* andrea.pinto@qi.ub.es

[b] *Institut de Nanociència i Nanotecnologia (IN²UB). Universitat de Barcelona, 08028 Barcelona, Spain*

[c] *Departament de Química, Universitat de les Illes Balears, 07071 Palma de Mallorca, Spain- e-mail:* toni.frontera@uib.es

Supporting Information

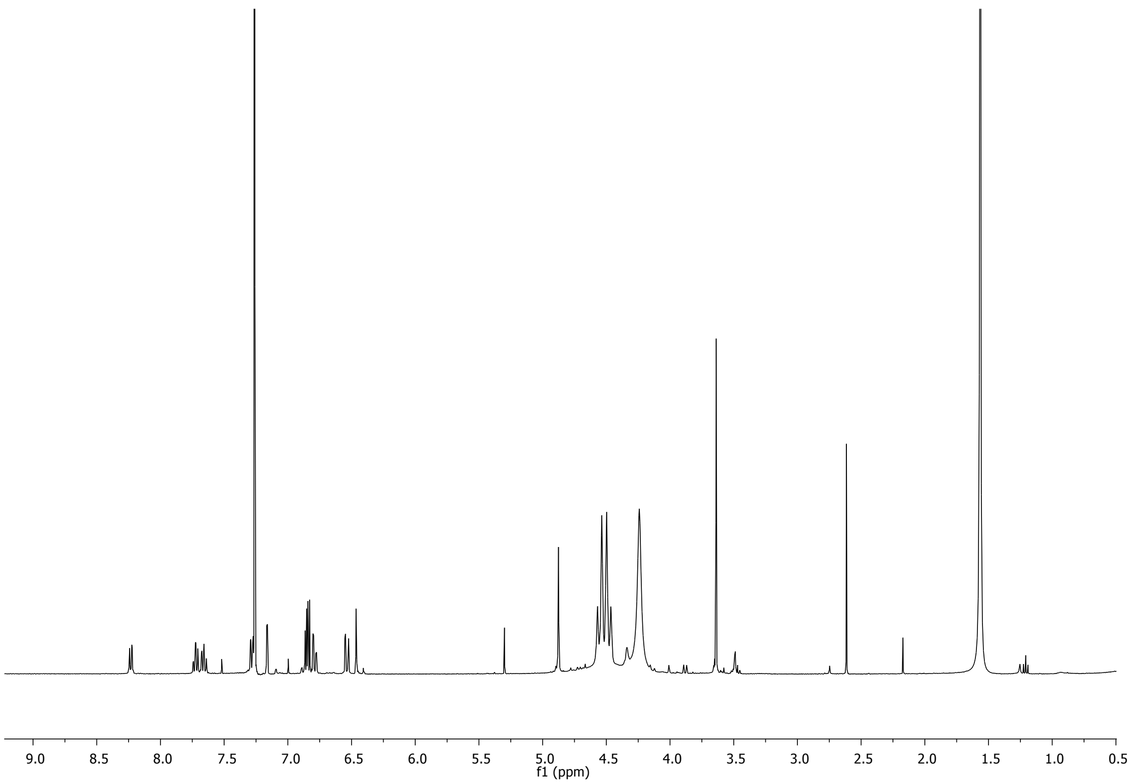

Figure S1. ^{1}H NMR spectrum of **1** in $CDCl_3$.

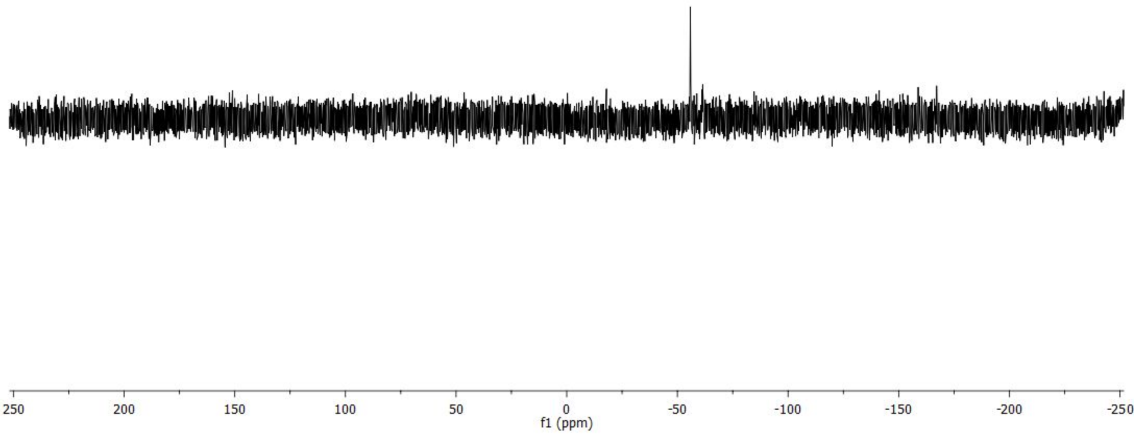

Figure S2. ^{31}P NMR spectrum of **1** in $CDCl_3$.

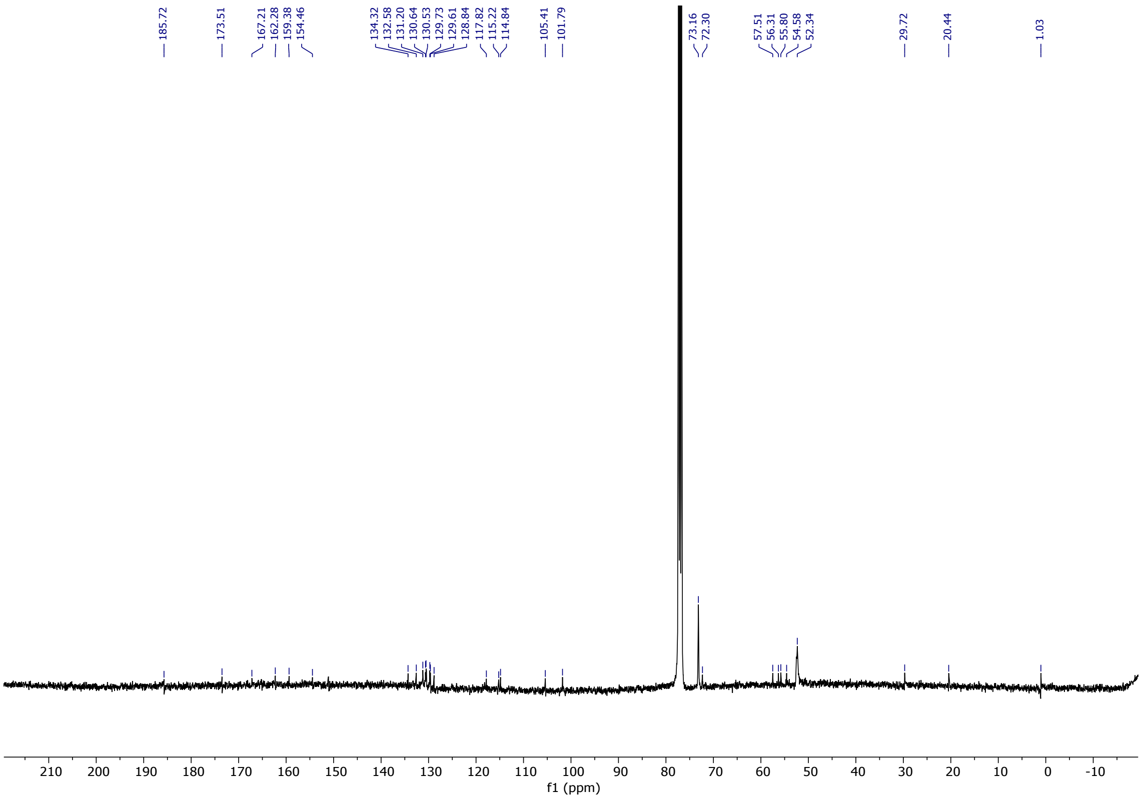

Figure S3. ^{13}C NMR spectrum of **1** in $CDCl_3$.

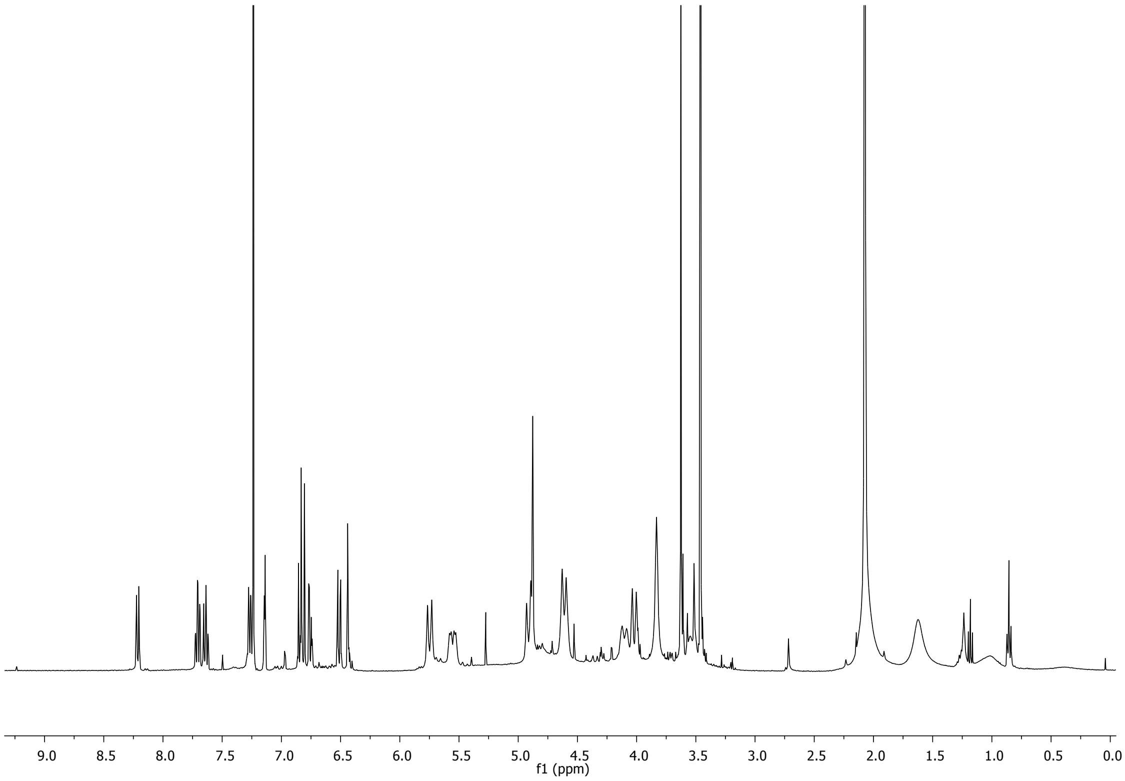

Figure S4. ^{1}H NMR spectrum of **2** in $CDCl_3$.

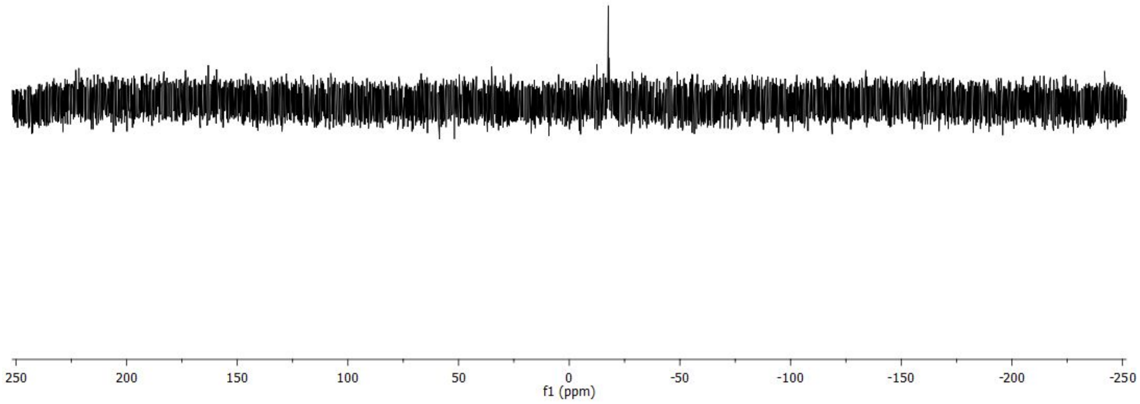

Figure S5. ^{31}P NMR spectrum of **1** in $CDCl_3$.

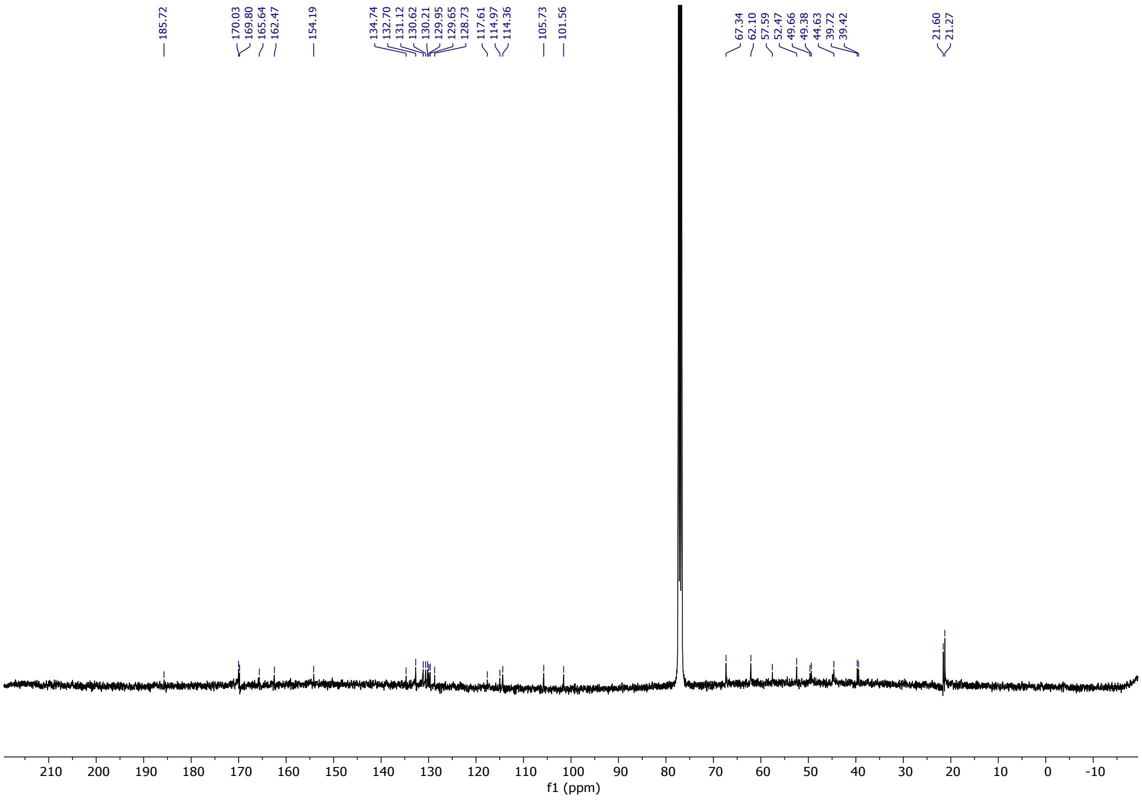

Figure S6. ^{13}C NMR spectrum of **2** in $CDCl_3$.

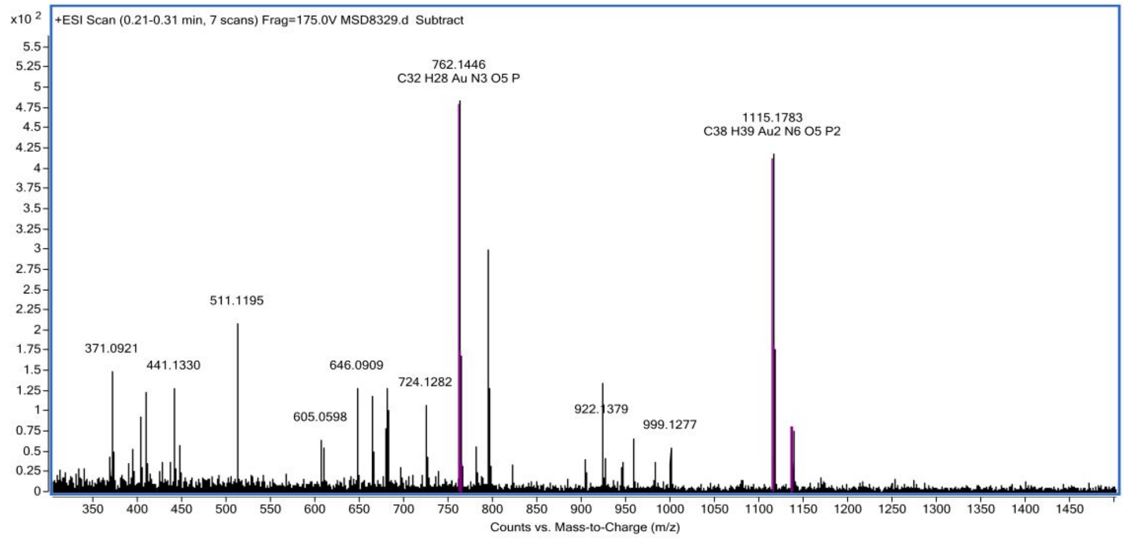

Figure S7. HR-Mass spectra of **1**.

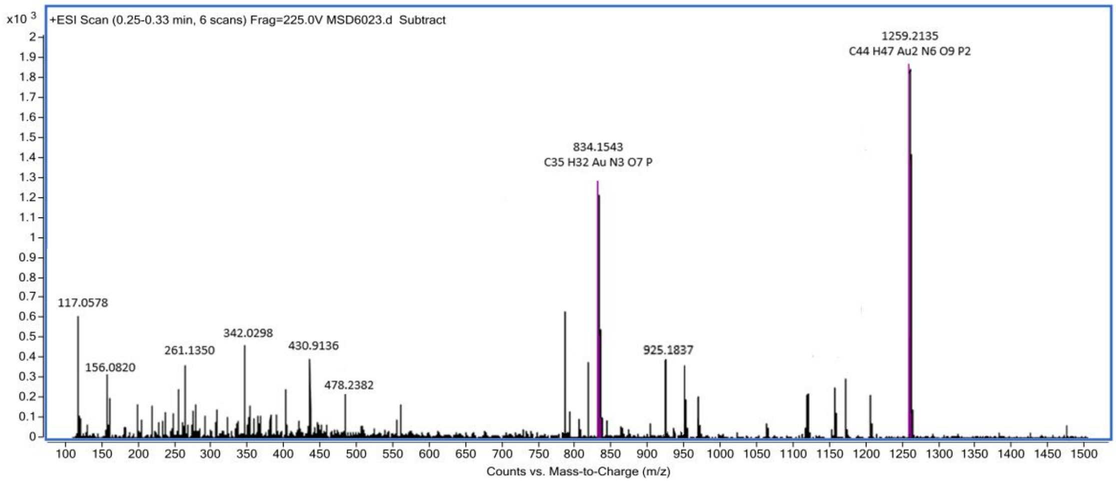

Figure S8. HR-Mass spectra of **2**.

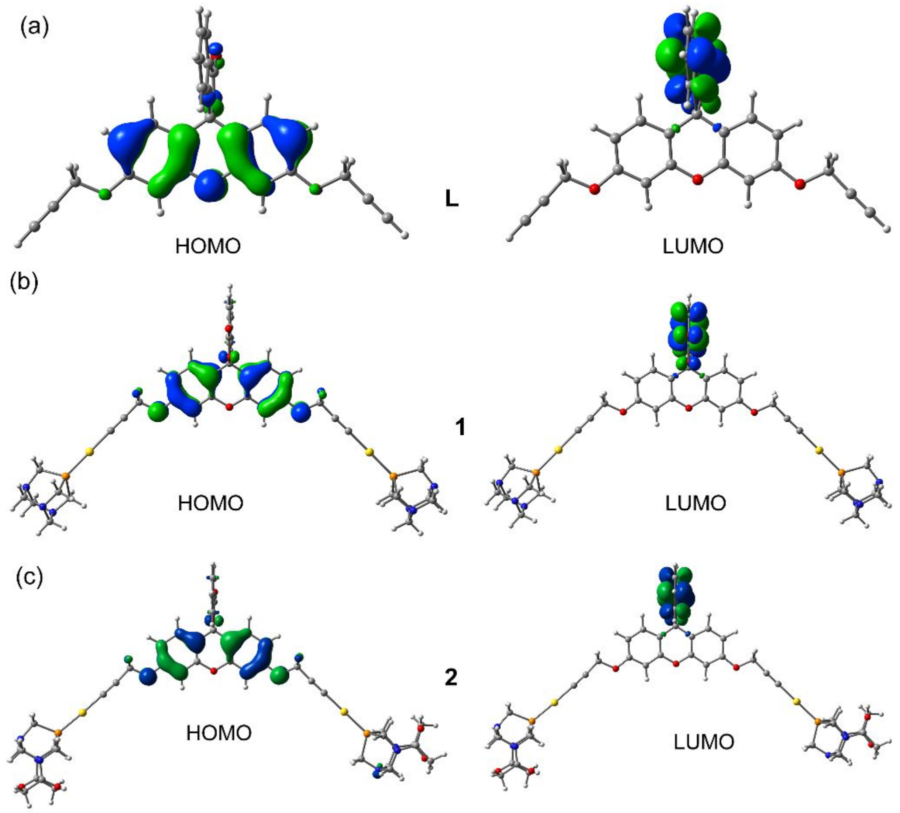

Figure S9. HOMO-LUMO plots of the **L** (a) and compounds **1** (b)and **2** (c). Isosurface 0.04 a.u. Level of theory RI-BP86-D3/def2-TZVP.

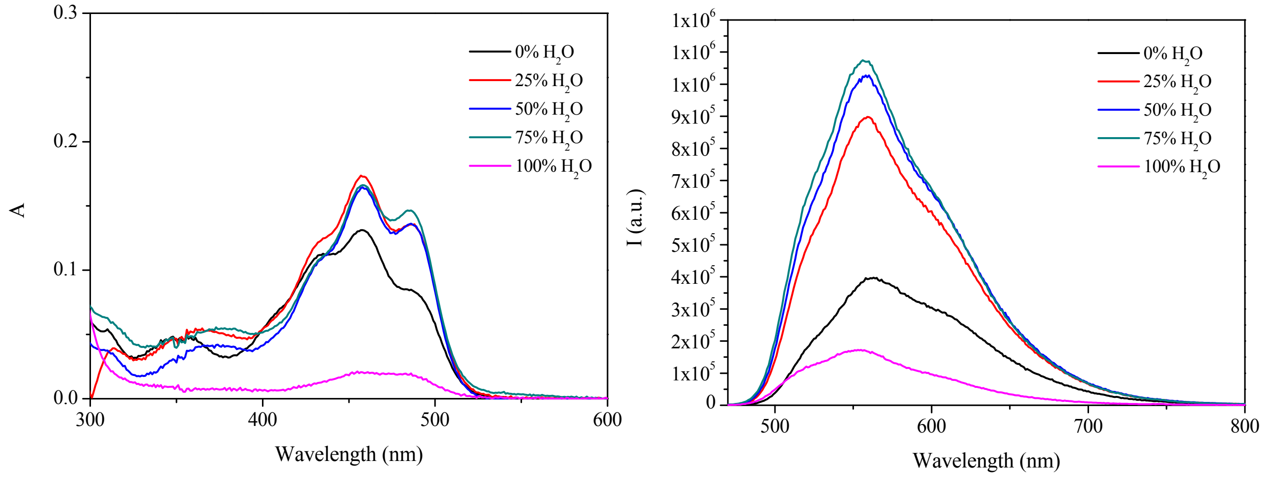

Figure S10. Absorption (left) and emission (right) spectra of **1** in acetonitrile/water mixtures.

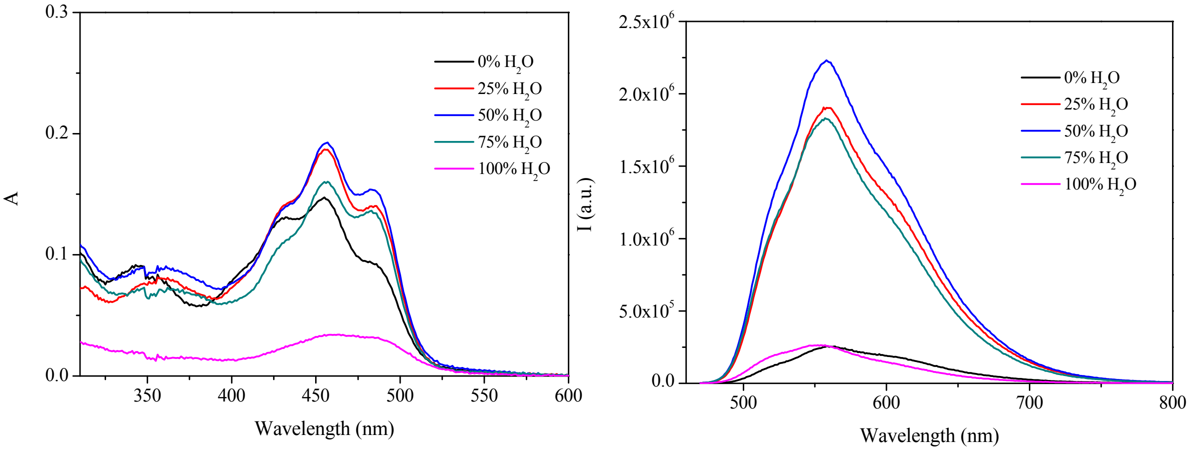

Figure S11. Absorption (left) and emission (right) spectra of **2** in acetonitrile/water mixtures.

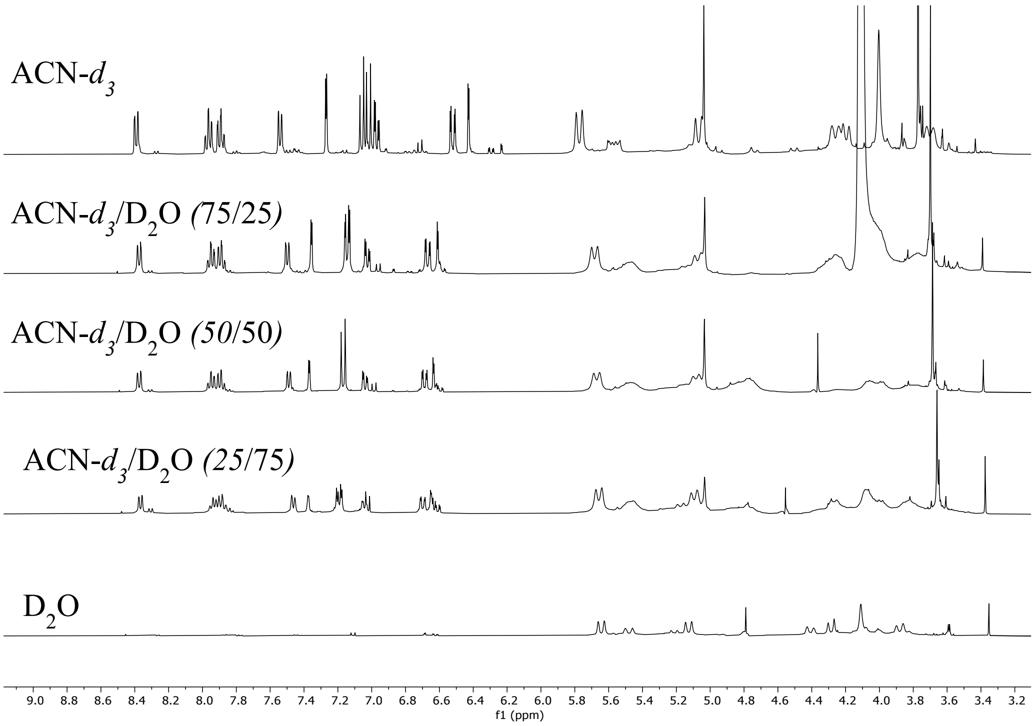

Figure S12. ^{1}H NMR spectra of **2** in ACN-d_3/D_2O mixtures.

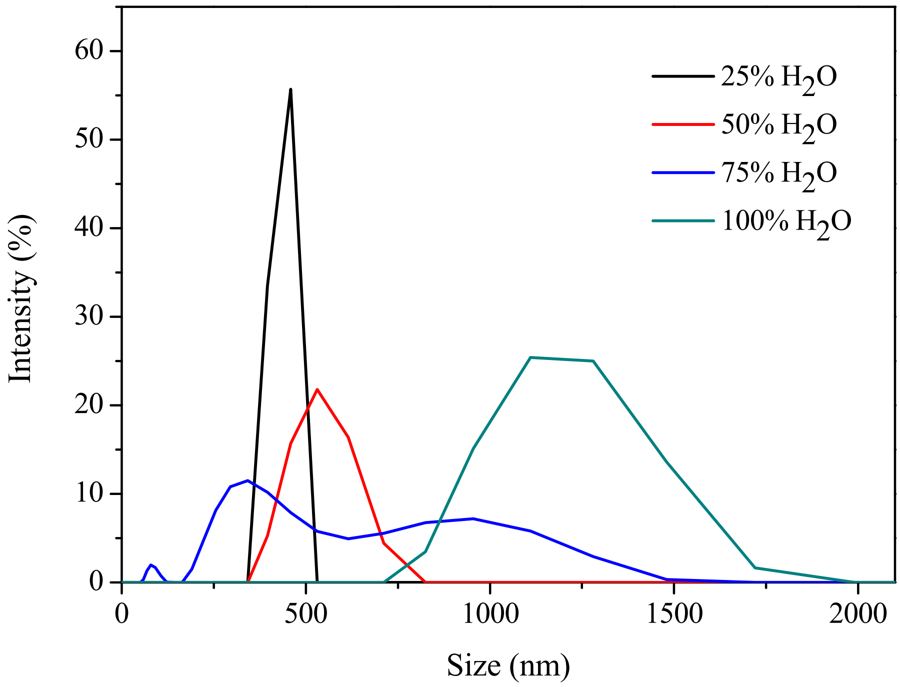

Figure S13. Size distribution obtained by Dynamic Light Scattering (DLS) for **1** at different water/acetonitrile ratios.

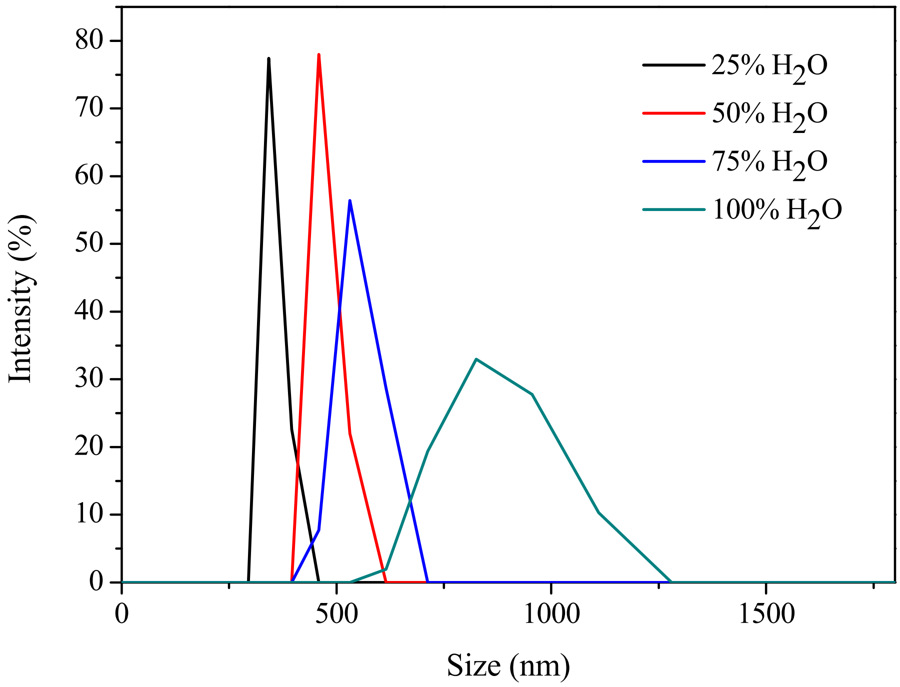

Figure S14. Size distribution obtained by Dynamic Light Scattering (DLS) for **2** at different water/acetonitrile ratios.

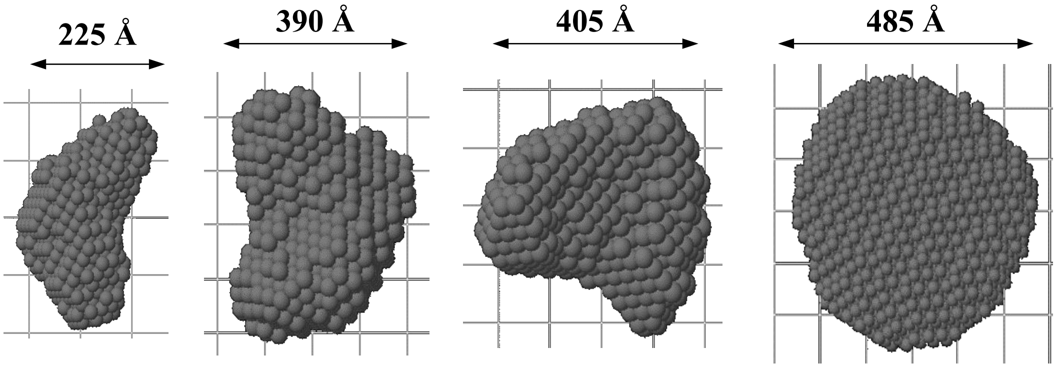

Figure S15. DAMMIN low resolution structures reconstructed from SAXS patterns for **1** at different water/acetonitrile ratios.

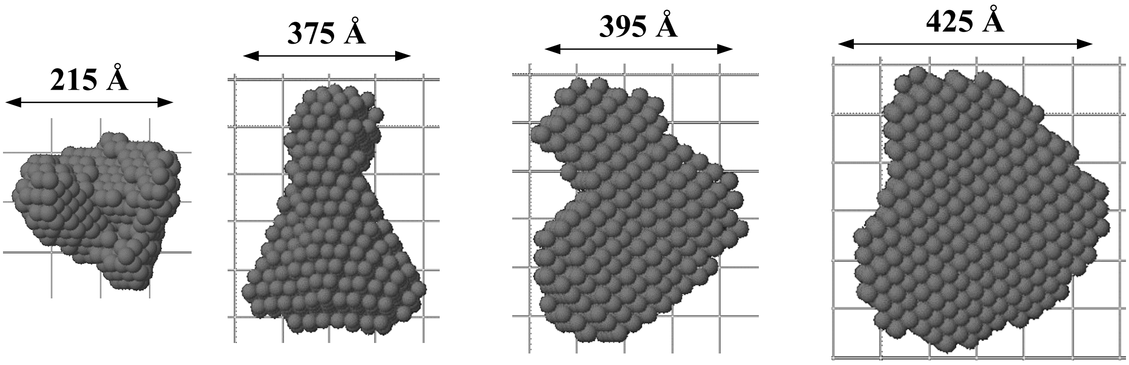

Figure S16. DAMMIN low resolution structures reconstructed from SAXS patterns for **2** at different water/acetonitrile ratios.

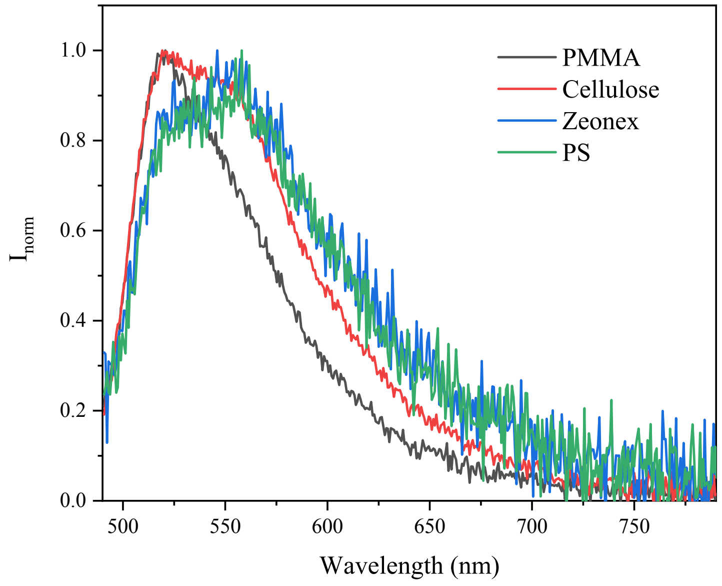

Figure S17. Emission spectra for **L** in different polymer matrixes upon excitation on the absorption maxima.

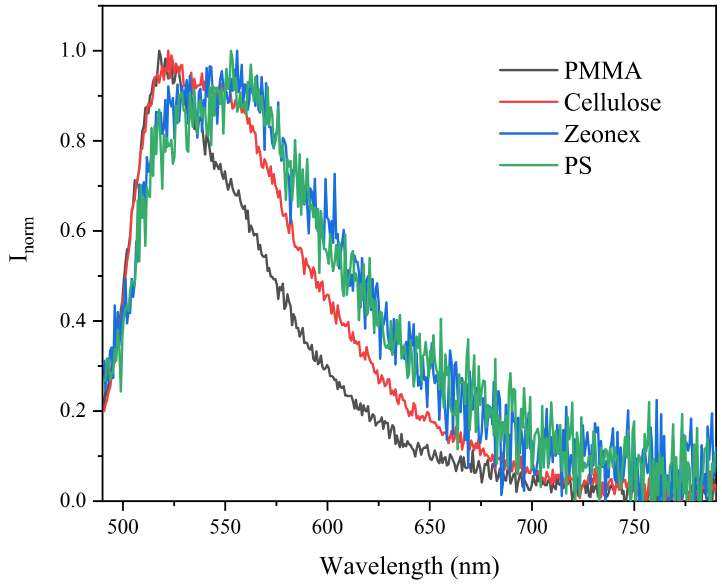

Figure S18. Emission spectra for **1** in different polymer matrixes upon excitation on the absorption maxima.

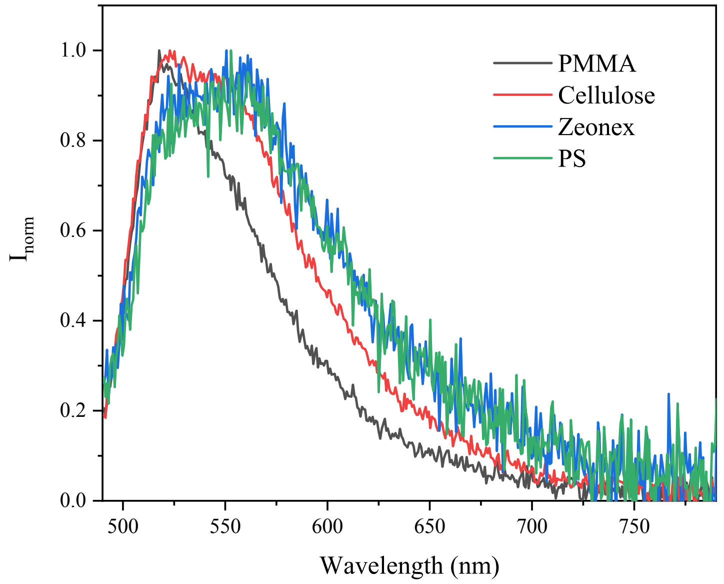

Figure S19. Emission spectra for **2** in different polymer matrixes upon excitation on the absorption maxima.

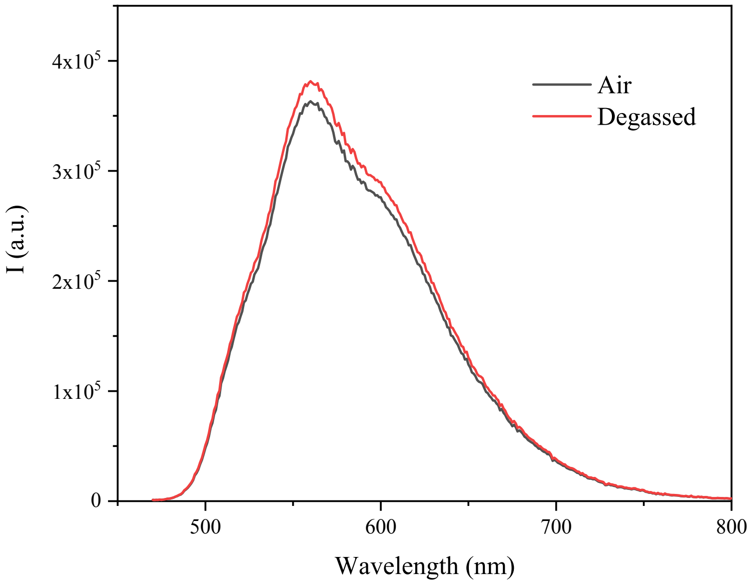

Figure S20. Emission spectra from aerated and degassed **L** acetonitrile solution.

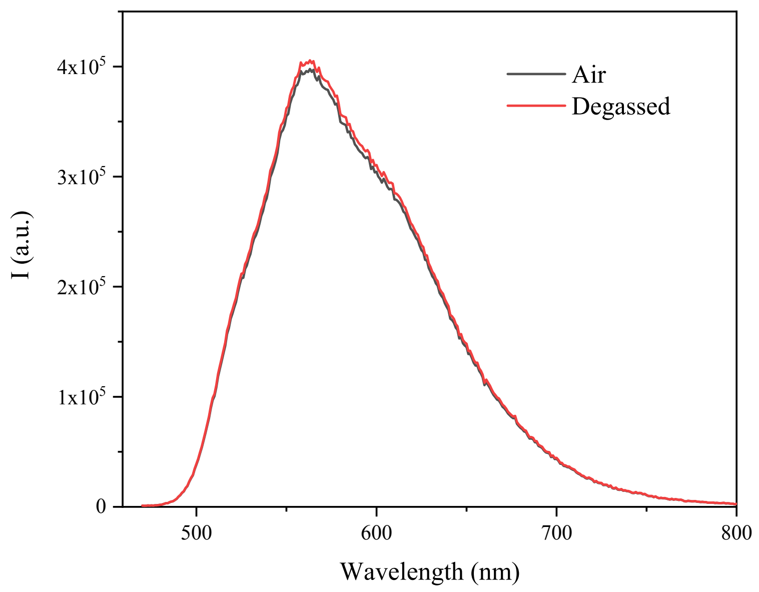

Figure S21. Emission spectra from aerated and degassed **1** acetonitrile solution.

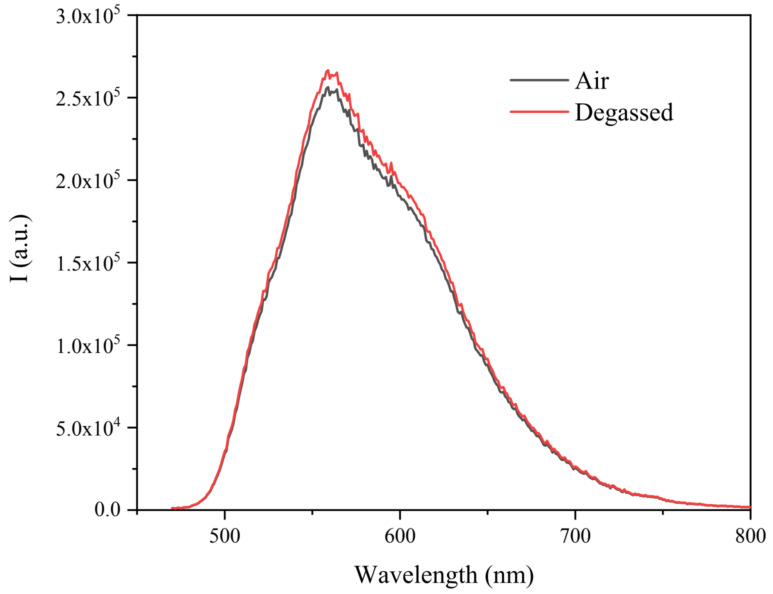

Figure S22. Emission spectra from aerated and degassed **2** acetonitrile solution.

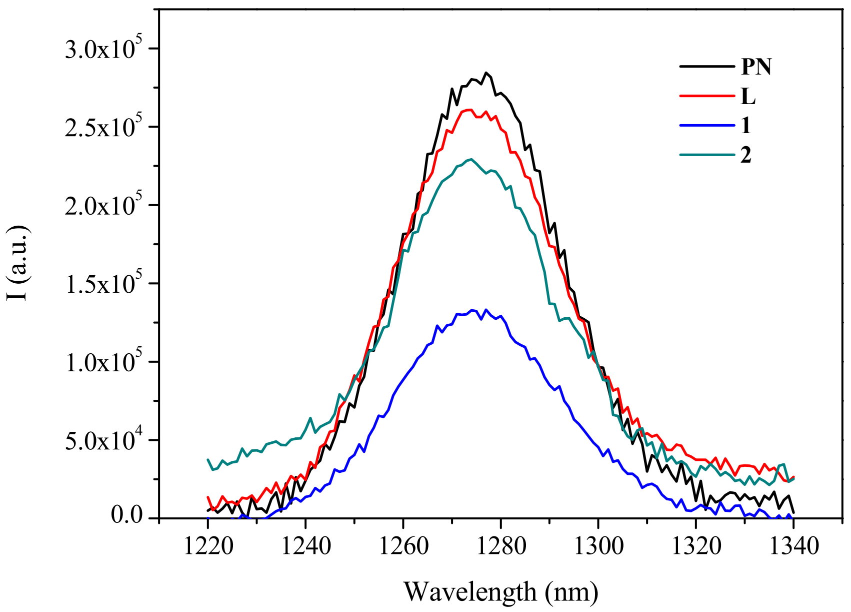

Figure S23. Singlet oxygen spectra for 1H-phenal-1-one (PN), **L** and gold(I) complexes **1** and **2** in dichloromethane upon excitation on the absorption maxima.

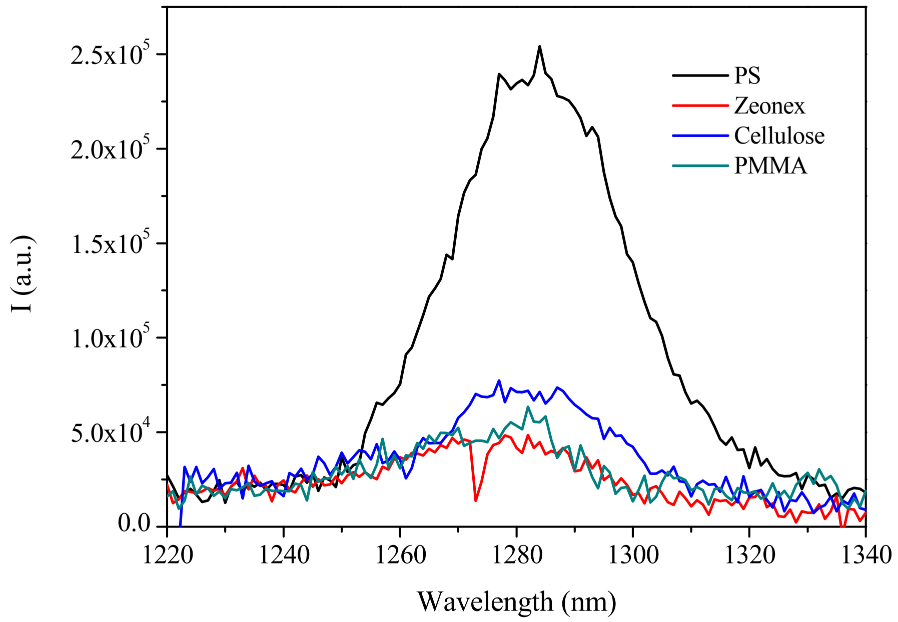

Figure S24. Singlet oxygen spectra for **L** in different polymer matrixes upon excitation on the absorption maxima.

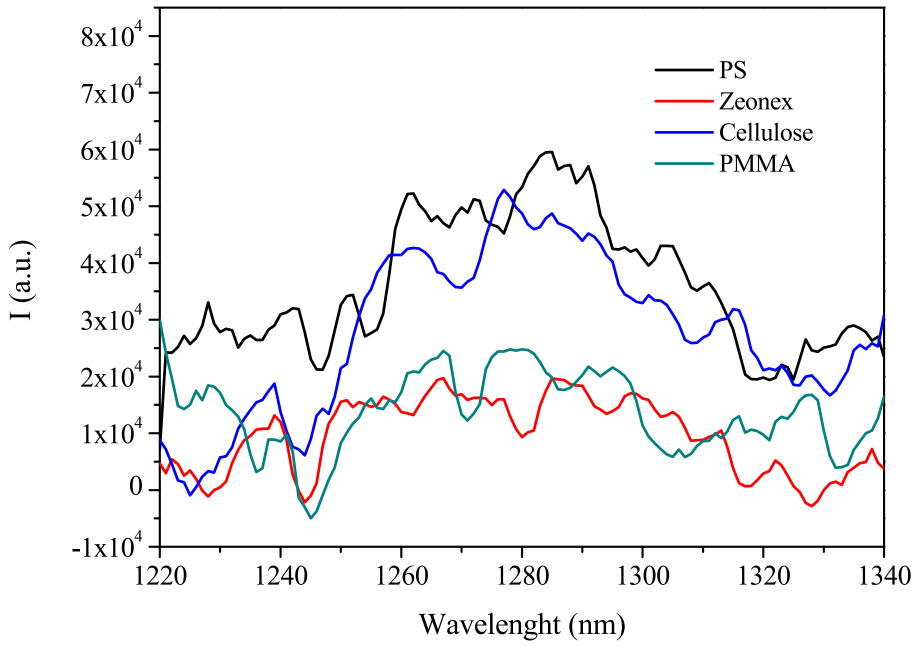

Figure S25. Singlet oxygen spectra for **1** in different polymer matrixes upon excitation on the absorption maxima.

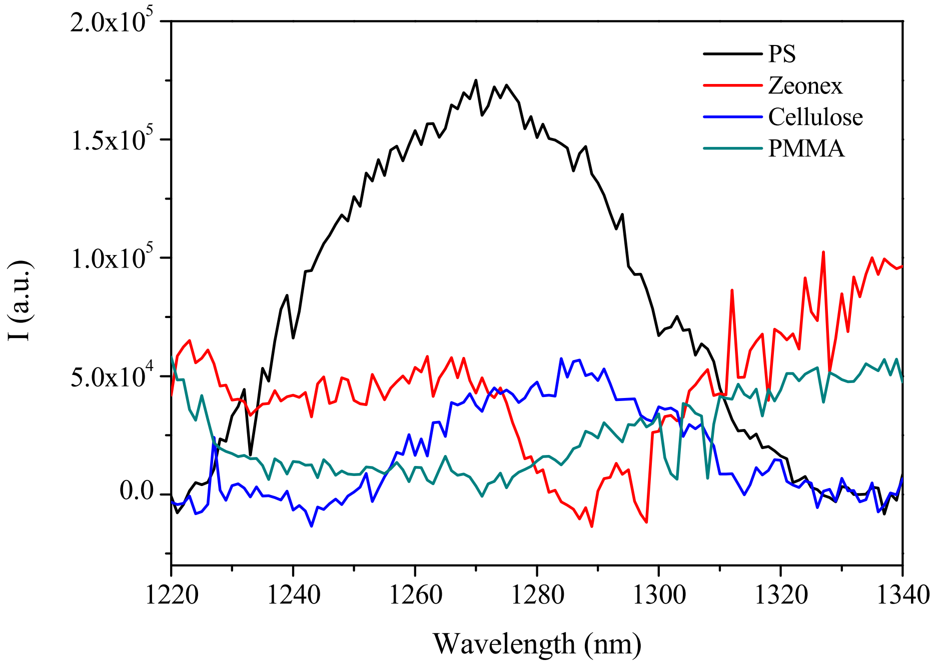

Figure S26. Singlet oxygen spectra for **2** in different polymer matrixes upon excitation on the absorption maxima.

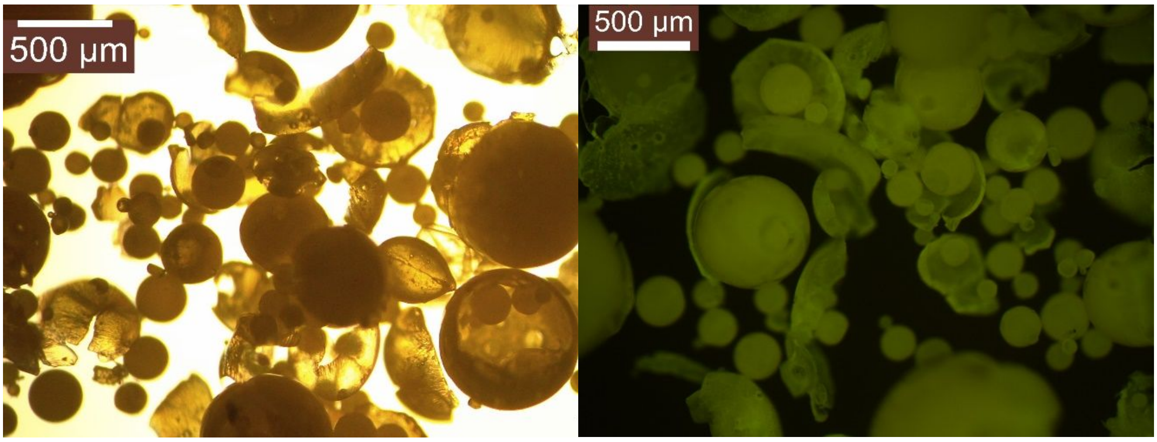

Figure S27. Optical microscopy image of the PS microspheres of **L** under light (left) and UV-light (right).

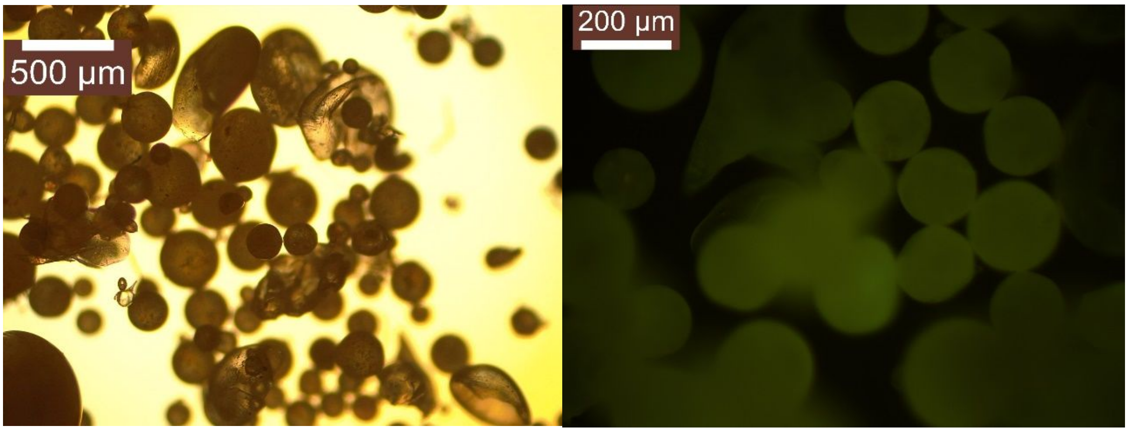

Figure S28. Optical microscopy image of the PS microspheres of **1** under light (left) and UV-light (right).

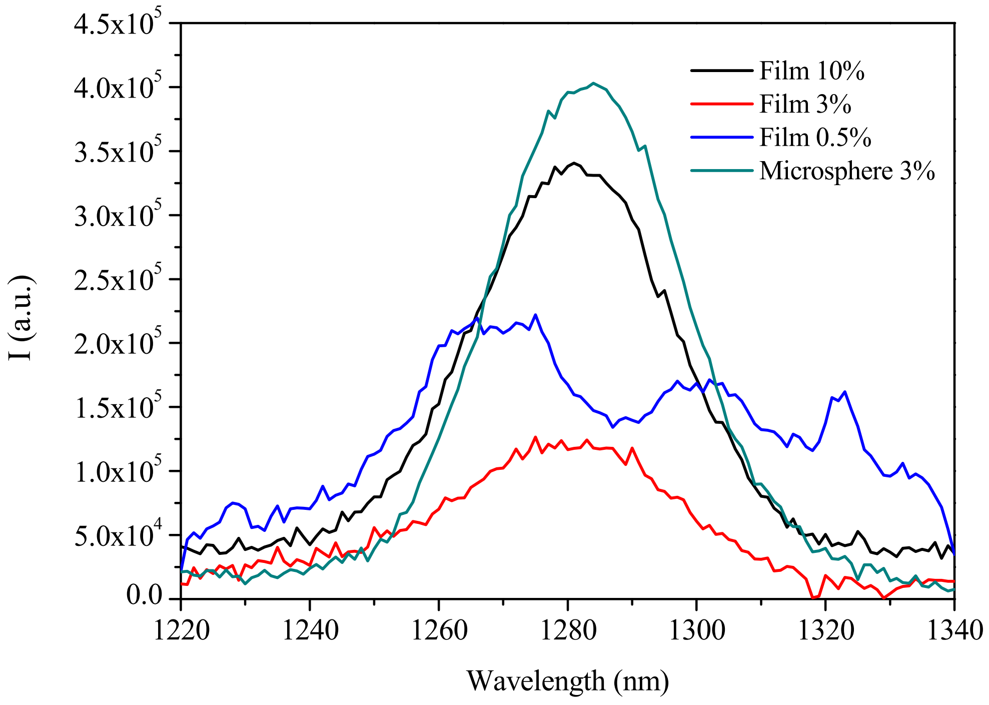

Figure S29. Singlet oxygen spectra for **L** in PS at different concentrations and shapes upon excitation on the absorption maxima.

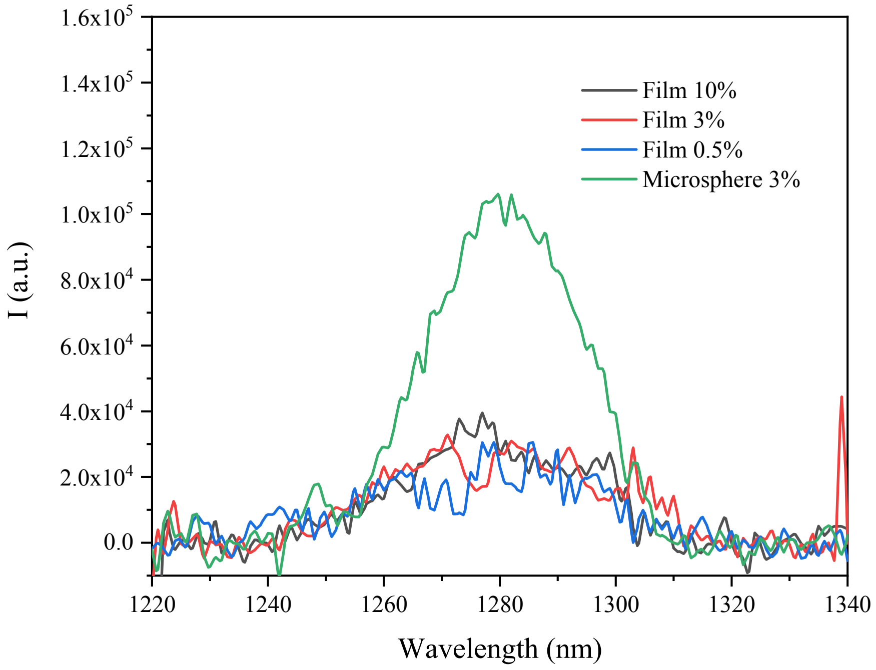

Figure S30. Singlet oxygen spectra for **1** in PS at different concentrations and shapes upon excitation on the absorption maxima.

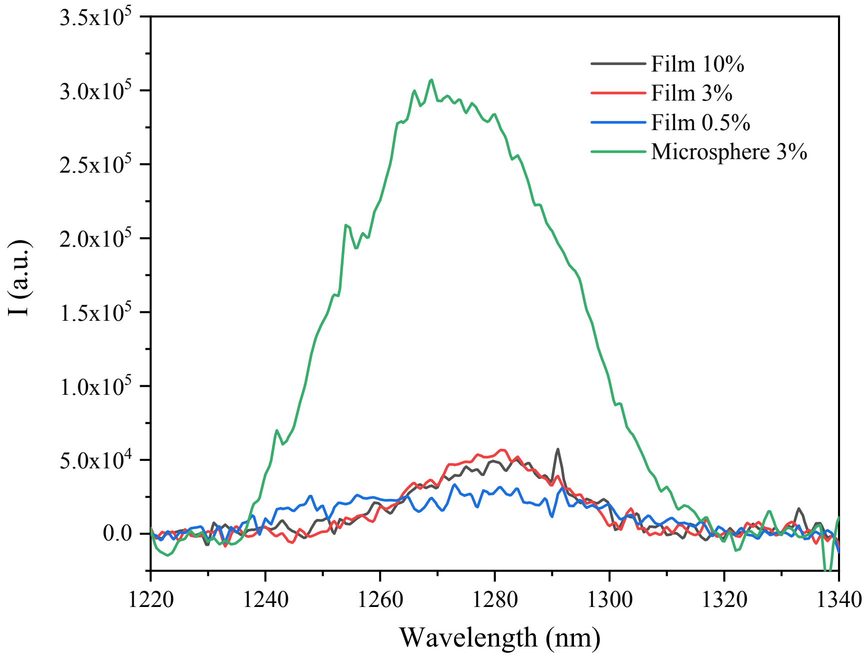

Figure S31. Singlet oxygen spectra for **2** in PS at different concentrations and shapes upon excitation on the absorption maxima.

Cartesian Coordinates:

L singlet

C	0.304324000	9.128974900	6.468784900
C	1.035232400	9.337301900	7.637023800
H	1.568405100	10.274615500	7.787827000
C	1.082450600	8.335818300	8.609636200
C	0.387244700	7.128261800	8.410885900
H	0.404858200	6.330770700	9.150228600
C	-0.335362700	6.951951900	7.235215700
H	-0.867203400	6.012689000	7.074849800
C	-0.393767800	7.933662700	6.239985300
C	-1.123232600	7.686488800	4.946163000
C	-1.185149500	8.932134600	4.102214600
C	-1.933802900	8.968501000	2.920136800
H	-2.506809300	8.084149300	2.637141100
C	-1.960365900	10.085623600	2.091213200
H	-2.554032900	10.059532000	1.180431000
C	-1.212817700	11.221720400	2.454284400
C	-0.461821500	11.216924300	3.632223500
H	0.115234500	12.094497900	3.918849900
C	-0.451911800	10.080052400	4.438739400
C	-2.474964700	7.025977000	5.136845300
C	-3.610690200	7.510443400	5.779339000
H	-3.616041200	8.498216700	6.241502400
C	-4.742087100	6.688716900	5.810267900
H	-5.647795500	7.041197300	6.305835800
C	-4.735160200	5.415563800	5.213702700
H	-5.634086600	4.799661700	5.254732200

C	-3.591383900	4.938021000	4.571886700
H	-3.557844000	3.955229700	4.100612400
C	-2.469323000	5.767362400	4.547568100
C	-1.136430200	5.527005600	3.943241700
O	1.828756300	8.634314200	9.716787100
O	0.313866300	10.172756300	5.575671500
O	-1.155284900	12.382491100	1.731720800
O	-0.361488000	6.641334600	4.178019100
O	-0.726894400	4.561045400	3.340046800
C	-1.882936800	12.422357700	0.490184500
H	-1.526792600	11.621593500	-0.184166300
H	-2.961004100	12.258075900	0.674199500
C	1.925787500	7.624512200	10.738115500
H	0.921128600	7.385704300	11.133991700
H	2.354522700	6.696859500	10.316372000
C	2.775041600	8.117856800	11.809798000
C	3.478563500	8.493065900	12.718848000
C	-1.680521300	13.720307900	-0.132008100
C	-1.532120800	14.789501900	-0.676817200
H	4.103064400	8.832099600	13.519376700
H	-1.395901100	15.737554900	-1.154739900

L triplet

C	0.2591313	9.1846318	6.4722687
C	1.0140819	9.3604541	7.6154477
H	1.5400130	10.2978684	7.7889003
C	1.0984335	8.3192762	8.5592592
C	0.4015097	7.1159977	8.3452066
H	0.4435162	6.3006269	9.0639872
C	-0.3490201	6.9595930	7.1899289
H	-0.8865725	6.0265965	7.0256708
C	-0.4357467	7.9709367	6.1963069
C	-1.2118345	7.8627004	4.9956998
C	-1.2800234	8.9977298	4.1320287
C	-1.9819139	9.0323055	2.9024305
H	-2.5139102	8.1377890	2.5806475
C	-2.0055913	10.1608433	2.0888421
H	-2.5634977	10.1265964	1.1555120
C	-1.3004996	11.3124677	2.4787360
C	-0.5683723	11.3112949	3.6781877
H	-0.0023894	12.1928717	3.9744007
C	-0.5610982	10.1792612	4.4723049
C	-1.9108386	6.6065429	4.6585412
C	-3.3166029	6.5839634	4.6138343
H	-3.8601162	7.5036815	4.8352270
C	-4.0127484	5.4133276	4.3161416
H	-5.1036774	5.4208712	4.3065323
C	-3.3155071	4.2311028	4.0420513
H	-3.8563420	3.3123779	3.8131356
C	-1.9234708	4.2378067	4.0582474
H	-1.3552091	3.3360423	3.8252004
C	-1.2145236	5.4100150	4.3614261
C	0.2743559	5.3515484	4.2922723
O	1.8763700	8.5951958	9.6441295
O	0.2049326	10.2536939	5.6134200
O	-1.2450289	12.4810974	1.7708616
O	1.0240357	6.2621552	4.7851830
O	0.8789701	4.3970281	3.7324545
C	-1.9595643	12.5294996	0.5217788
H	-1.5844883	11.7437482	-0.1599482
H	-3.0364396	12.3453182	0.6936757

C	2.0253115	7.5603330	10.6381725
H	1.0372519	7.2965062	11.0578397
H	2.4538257	6.6535270	10.1742366
C	2.9008290	8.0452187	11.6912432
C	3.6267369	8.4183714	12.5832068
C	-1.7687072	13.8392823	-0.0783724
C	-1.6279784	14.9184546	-0.6052347
H	4.2721501	8.7544162	13.3683390
H	-1.4989290	15.8759760	-1.0659424

1 singlet

C	-0.314602700	8.465713400	6.339071500
C	0.276700100	8.811436700	7.552068300
H	0.797947100	9.761908600	7.653324000
C	0.194600400	7.936126500	8.640303800
C	-0.490914200	6.712464300	8.501132800
H	-0.572506900	6.010596400	9.327517000
C	-1.072262500	6.396483700	7.277508400
H	-1.594721900	5.444519700	7.168616600
C	-1.000846200	7.252429500	6.171860000
C	-1.569264100	6.860410800	4.835754900
C	-1.605357000	8.030332900	3.891160100
C	-2.297555900	7.969600200	2.675606800
H	-2.862970400	7.066313300	2.440557800
C	-2.279166400	9.013553700	1.756352200
H	-2.832644500	8.915428700	0.825632500
C	-1.537127200	10.174879600	2.051439700
C	-0.842841000	10.264153300	3.262847800
H	-0.275527600	11.161968000	3.502599500
C	-0.884497600	9.203536900	4.164764700
C	-2.888584800	6.122063800	4.931439100
C	-4.117031600	6.572114300	5.407077700
H	-4.231693900	7.587764100	5.787583700
C	-5.194169100	5.680699600	5.379095900
H	-6.169425500	6.005462200	5.744401700
C	-5.044146400	4.371925000	4.887948700
H	-5.903597000	3.700673000	4.878680200
C	-3.808240500	3.928770600	4.414659900
H	-3.662273600	2.919342700	4.028402000
C	-2.741743800	4.827945300	4.447441200
C	-1.336187800	4.634335600	4.012570700
O	0.800977200	8.365674300	9.782136500
O	-0.186814400	9.394506400	5.333783500
O	-1.435268900	11.264122400	1.238833800
O	-0.660500700	5.808132400	4.236738800
O	-0.809590100	3.651446600	3.538284000
C	-2.147949300	11.225117300	-0.024359800
H	-1.807427500	10.349886600	-0.608869600
H	-3.229609100	11.101710300	0.172699300
C	0.753267000	7.486905600	10.935253600
H	-0.302564900	7.291677500	11.201187100
H	1.221735200	6.519127400	10.674353700
C	1.450158800	8.105502100	12.045365800
C	2.029817100	8.590689000	13.009988500
C	-1.898336500	12.449837700	-0.758407300
C	-1.705577200	13.466158300	-1.415583100
Au	2.983327200	9.402026200	14.552415700
P	4.083424900	10.330394800	16.305658100
N	4.831619700	10.208440700	18.980592700
N	4.435908000	12.468538600	18.047067700
N	6.474506800	11.208381400	17.419748600

C	4.063198600	9.469808300	17.969145300
H	3.018444500	9.361828900	18.296133700
H	4.477547900	8.458169400	17.847148000
C	3.613121600	12.040771500	16.907315400
H	3.726772500	12.750492500	16.074369100
H	2.550301300	12.034437500	17.191980000
C	5.932651500	10.606778800	16.193416200
H	6.421290000	9.639806700	16.001155200
H	6.140338100	11.258339000	15.331832700
C	5.866072700	12.525051800	17.689391700
H	5.991374800	13.167728000	16.805678700
H	6.407463700	12.981219000	18.531570300
C	6.248676300	10.341590000	18.591763800
H	6.661823700	9.342956100	18.386859400
H	6.793329600	10.775860100	19.443281900
C	4.279703200	11.559375800	19.198301100
H	3.212664200	11.476761300	19.450760600
H	4.807109600	12.005031800	20.054905600
Au	-1.332151900	15.107423100	-2.470752000
P	-0.875805300	16.975262000	-3.674274100
N	-0.555459900	19.735253800	-3.765297300
N	-1.512620400	18.668867700	-5.785127500
N	0.895241200	18.405854700	-5.268835000
C	-0.875570900	18.647413300	-2.830528400
H	-1.866812300	18.815497600	-2.383816300
H	-0.143451000	18.628970700	-2.009415300
C	-1.963129600	17.434186100	-5.128627300
H	-1.957826200	16.602424900	-5.849189200
H	-2.996777500	17.553825100	-4.771473800
C	0.776730700	17.134600500	-4.541323800
H	1.577497100	17.054568100	-3.790913800
H	0.892586100	16.290601300	-5.237297300
C	-0.141268800	18.531553400	-6.310580400
H	-0.094837900	17.654690900	-6.973114000
H	0.084043100	19.429570100	-6.905090300
C	0.783246200	19.561440600	-4.359565800
H	1.525366900	19.460177200	-3.553831200
H	1.017042900	20.468926500	-4.936174300
C	-1.542565000	19.815675600	-4.858499600
H	-2.550697800	19.905846200	-4.428059200
H	-1.329417200	20.725207900	-5.439746700

1 triplet

C	0.290978100	9.220098900	6.476811800
C	1.044572300	9.395642200	7.619997800
H	1.569436700	10.333668200	7.794031800
C	1.131644500	8.355385500	8.568295700
C	0.433389900	7.150999300	8.351013900
H	0.477138800	6.336103300	9.070003400
C	-0.315543600	6.994796300	7.195623800
H	-0.851916700	6.061274800	7.030431300
C	-0.402995000	8.005661700	6.199782900
C	-1.184503900	7.899789800	5.001578400
C	-1.256152500	9.037105500	4.141283100
C	-1.961109000	9.075652300	2.912696100
H	-2.494427500	8.181870900	2.590765600
C	-1.986444800	10.204526200	2.100701300
H	-2.546029400	10.172854000	1.168463300
C	-1.279821900	11.358656200	2.488161700
C	-0.545228000	11.351157800	3.688396800
H	0.022060500	12.231929000	3.984861500

C	-0.535673700	10.218426200	4.480459900
C	-1.885926700	6.645401100	4.665669100
C	-3.292242400	6.629376200	4.610794500
H	-3.832290800	7.553463500	4.822528700
C	-3.993096400	5.461299300	4.315719500
H	-5.084092600	5.474961200	4.298569500
C	-3.300271800	4.273362700	4.054255900
H	-3.844396300	3.355725400	3.827482900
C	-1.908300100	4.273855800	4.079188200
H	-1.341912900	3.368914900	3.854058700
C	-1.193805300	5.443476500	4.379157400
C	0.297917400	5.375543900	4.313600300
O	1.904794900	8.632805200	9.647957300
O	0.235416700	10.290741300	5.619225900
O	-1.224917700	12.523154000	1.785205600
O	1.043226600	6.289282500	4.808337300
O	0.890484400	4.413134000	3.756052800
C	-1.937492700	12.577579700	0.521591200
H	-1.552245100	11.783365300	-0.144894400
H	-3.010599800	12.378402300	0.701833700
C	2.055693800	7.598275500	10.660494600
H	1.057938400	7.341047500	11.061443000
H	2.477417300	6.693273500	10.186382000
C	2.924187000	8.080670800	11.714131400
C	3.656914000	8.452807900	12.622962600
C	-1.753999800	13.882885400	-0.080529300
C	-1.620796100	14.973642100	-0.622838100
Au	-1.382325700	16.750232400	-1.479846500
P	-1.106858900	18.779084400	-2.456612300
C	0.579655000	19.282155400	-3.097286300
C	-1.480832100	20.338035600	-1.487971000
C	-2.090007500	19.202560200	-3.993823000
N	0.562446300	20.622340100	-3.700087200
H	1.296881600	19.254128900	-2.263410700
H	0.908458300	18.540040400	-3.839782800
N	-1.247878400	21.549879700	-2.286557900
H	-2.529107700	20.302422800	-1.156428600
H	-0.849893000	20.354365200	-0.587210800
N	-1.784789500	20.551843100	-4.488813700
H	-1.864759700	18.457169500	-4.770707600
H	-3.162129300	19.121760800	-3.760014600
C	-0.360684900	20.685927900	-4.849434800
C	0.158306000	21.649363300	-2.721072900
C	-2.108966600	21.581976800	-3.483959700
H	-0.094957200	19.898557400	-5.569556400
H	-0.226444600	21.663402100	-5.336614100
H	0.813477000	21.585776400	-1.839978900
H	0.298563100	22.635355800	-3.188841300
H	-3.158953600	21.468559400	-3.176508400
H	-1.990096000	22.567086600	-3.959519500
Au	4.855858100	9.074307900	14.080930900
P	6.227098400	9.781784200	15.744861100
C	5.737740600	11.258073800	16.788991700
C	7.973821800	10.326635400	15.345173100
C	6.648055600	8.615602900	17.148802400
N	6.743590500	11.562504500	17.816207200
H	5.599399500	12.126850500	16.128449900
H	4.768009100	11.046488100	17.263522200
N	8.707648600	10.744703700	16.547402400
H	8.498692700	9.492826800	14.855883600
H	7.928889200	11.156288200	14.623889100

N	7.543053700	9.239956500	18.132780100
H	5.713870700	8.300742100	17.637020000
H	7.120751000	7.715641400	16.728889400
C	6.926071500	10.431579100	18.745439700
C	8.050419900	11.885429800	17.212782500
C	8.823807900	9.641946900	17.520386600
H	5.951113300	10.153733500	19.172304300
H	7.580721400	10.768722800	19.563018000
H	7.919981200	12.700317900	16.485790200
H	8.715428300	12.237842000	18.015361000
H	9.277156100	8.770319300	17.026300200
H	9.494833800	9.972706600	18.327199600

2 singlet

C	0.4167598	9.0966910	6.4184002
C	1.1680527	9.2976968	7.5746500
H	1.7403184	10.2155504	7.7002719
C	1.1808741	8.3195330	8.5743079
C	0.4312423	7.1382305	8.4042058
H	0.4223460	6.3568422	9.1602334
C	-0.3108977	6.9684577	7.2402850
H	-0.8851812	6.0501158	7.1064639
C	-0.3391565	7.9301218	6.2238925
C	-1.0969796	7.6947879	4.9464732
C	-1.1570825	8.9407067	4.1052399
C	-1.9641733	9.0092297	2.9631710
H	-2.5918625	8.1519282	2.7143386
C	-1.9829278	10.1238073	2.1315546
H	-2.6267033	10.1252526	1.2554766
C	-1.1620419	11.2259415	2.4437363
C	-0.3534788	11.1873876	3.5845742
H	0.2770830	12.0384806	3.8364486
C	-0.3573867	10.0562074	4.3983211
C	-2.4528529	7.0512744	5.1578399
C	-3.5674146	7.5460021	5.8292930
H	-3.5444333	8.5262492	6.3065622
C	-4.7135333	6.7454436	5.8688052
H	-5.6030002	7.1065984	6.3872136
C	-4.7424811	5.4826964	5.2509918
H	-5.6523646	4.8833197	5.2980089
C	-3.6196091	4.9944076	4.5809804
H	-3.6130747	4.0194201	4.0925967
C	-2.4822390	5.8021556	4.5499641
C	-1.1646117	5.5448420	3.9185347
O	1.9406489	8.6106700	9.6687584
O	0.4620426	10.1186560	5.5003606
O	-1.0845326	12.3728705	1.7103094
O	-0.3635001	6.6356776	4.1561138
O	-0.7896077	4.5774601	3.2933035
C	-1.8901558	12.4484752	0.5076309
H	-1.6400790	11.5982531	-0.1541171
H	-2.9597703	12.3650819	0.7780928
C	1.9757049	7.6260737	10.7315298
H	0.9461749	7.4407395	11.0916418
H	2.3714605	6.6733634	10.3330207
C	2.8074434	8.1012073	11.8203623
C	3.4910244	8.4361646	12.7804645
C	-1.6320587	13.7028332	-0.1717485
C	-1.4381221	14.7415705	-0.7914487
Au	4.5840145	8.9534531	14.3549442
Au	-1.0687001	16.3995366	-1.8172286

P	-0.6065673	18.2733727	-3.0066007
O	0.7529944	20.4919320	-7.2125196
O	-2.8475549	19.9893765	-5.8478605
N	0.7376054	20.6065361	-3.3271919
N	-1.6264627	20.6582225	-4.0413624
N	0.6217173	19.1842503	-5.3500333
C	0.2013671	17.9756068	-4.6603227
H	1.0596367	17.3120667	-4.4443508
H	-0.5089861	17.4047771	-5.2653579
C	-2.0089042	19.4476827	-3.3266945
H	-2.7904797	18.9366297	-3.9014197
H	-2.4127249	19.7001999	-2.3309644
C	0.5991585	19.5236221	-2.3525378
H	0.2333542	19.9146567	-1.3920301
H	1.5735040	19.0466857	-2.1728116
C	1.3795783	20.1735735	-4.5604306
H	2.3573081	19.7418062	-4.2871668
H	1.5434750	21.0318320	-5.2214727
C	-0.4816285	21.3941144	-3.4837360
H	-0.2620539	22.2498219	-4.1285324
H	-0.7555490	21.7704063	-2.4840252
C	0.3740478	19.4416194	-6.6909452
C	-0.3908492	18.3965926	-7.4779090
H	0.0683126	17.4000406	-7.4105280
H	-0.3984362	18.7181681	-8.5232324
H	-1.4294633	18.3378231	-7.1210603
C	-2.1324667	20.8429923	-5.3216256
C	-1.7974623	22.1427662	-6.0247503
H	-0.7607548	22.1263421	-6.3908457
H	-1.9382018	23.0182784	-5.3766997
H	-2.4596629	22.2212121	-6.8921172
P	5.8140314	9.4788981	16.1865158
O	9.9802283	10.9037471	18.4053745
O	6.9331031	13.1079463	17.5382698
N	6.7313421	8.8142939	18.6528291
N	6.0656984	11.1783049	18.3903322
N	8.4179083	9.8225521	17.1462758
C	7.6523137	9.6518247	15.9222454
H	7.9535576	8.7364184	15.3798465
H	7.8015850	10.4976304	15.2449545
C	5.3019289	10.9708344	17.1667915
H	5.3952286	11.8686113	16.5438684
H	4.2342545	10.8155164	17.4000820
C	5.8682193	8.2771149	17.6003759
H	4.8485956	8.1193264	17.9817627
H	6.2509797	7.3070042	17.2525518
C	8.1290708	8.8858494	18.2486369
H	8.4345080	7.8704842	17.9448680
H	8.7490645	9.2058456	19.0932605
C	6.1846599	10.0137976	19.2803872
H	6.8189278	10.2816201	20.1299268
H	5.1827424	9.7522734	19.6600942
C	9.3846038	10.8006876	17.3314146
C	9.6657788	11.7411050	16.1765183
H	9.8998948	11.2060145	15.2452238
H	10.5228926	12.3580250	16.4614061
H	8.8021776	12.4000930	16.0045483
C	6.8490605	12.3218232	18.4824107
C	7.5638744	12.5755894	19.7945520
H	8.4319892	11.9090615	19.8954274
H	6.9038414	12.4446004	20.6628176

H 7.9305263 13.6060805 19.7686471

2 triplet

C	0.296375900	9.228242600	6.479174300
C	1.050279900	9.403129800	7.622466500
H	1.574860000	10.341116500	7.797110600
C	1.137352400	8.362372200	8.569708300
C	0.439123600	7.158354600	8.352136700
H	0.482641100	6.343297300	9.070837000
C	-0.310465900	7.002765800	7.197070000
H	-0.847762000	6.069618700	7.031950000
C	-0.398089600	8.014128200	6.201923000
C	-1.179557400	7.908711500	5.003733800
C	-1.251511900	9.046405600	4.143662900
C	-1.956728300	9.084892000	2.915349400
H	-2.489505000	8.190906400	2.593132500
C	-1.982016300	10.214028000	2.103625500
H	-2.541537400	10.182356900	1.171514500
C	-1.274939700	11.367564600	2.490835600
C	-0.540001400	11.360342700	3.690510300
H	0.028102200	12.240704400	3.986331200
C	-0.530756800	10.227459000	4.482870600
C	-1.881445700	6.654550400	4.667494800
C	-3.287632100	6.639094500	4.612470200
H	-3.827456500	7.563284200	4.824293200
C	-3.988738400	5.471316500	4.316839600
H	-5.079611800	5.485543500	4.298934400
C	-3.296244900	4.283253000	4.055533500
H	-3.840731800	3.366034300	3.828652200
C	-1.904402200	4.282891100	4.080603500
H	-1.338677100	3.377599000	3.855407000
C	-1.189654400	5.452476900	4.380662200
C	0.301567800	5.384453900	4.314688600
O	1.911179100	8.638936700	9.650197600
O	0.240590800	10.299124300	5.621604500
O	-1.219609200	12.532281200	1.786573000
O	1.048804200	6.297561600	4.808259800
O	0.895175800	4.422703100	3.757560700
C	-1.935107600	12.585342500	0.526049200
H	-1.552701500	11.791581500	-0.142752800
H	-3.008283800	12.387563100	0.707697800
C	2.062898100	7.602440600	10.658532300
H	1.066517000	7.343214800	11.061973100
H	2.484865800	6.697973800	10.183448000
C	2.933034900	8.082510300	11.712606300
C	3.667114400	8.448964300	12.622204500
C	-1.752755400	13.890564500	-0.077689300
C	-1.623529400	14.980197100	-0.622416600
Au	-1.395649800	16.751039600	-1.489707800
P	-1.113386700	18.774950300	-2.472741900
C	-0.731511700	18.731341100	-4.297553300
C	-2.483037600	20.012306100	-2.270891000
C	0.281445800	19.878788500	-1.941925400
N	-0.412252700	20.026795900	-4.874700300
H	0.111877000	18.022963100	-4.398648500
H	-1.597035500	18.283490100	-4.794292500
N	-2.205400900	21.315106500	-2.861466500
H	-3.402124300	19.616958100	-2.719572600
H	-2.636852000	20.107119600	-1.182104800
N	0.253356200	21.101795900	-2.745962300
H	0.171374500	20.117798500	-0.873973900

H	1.241385700	19.359184100	-2.073447500
C	0.565611700	20.858236000	-4.147511900
C	-0.951138800	20.505448200	-6.060483500
C	-0.922455900	21.928523700	-2.486572200
C	-2.985292700	21.719572600	-3.937015800
H	1.551790300	20.365230200	-4.182326800
H	0.624444100	21.807312500	-4.691132600
O	-0.641271700	21.618062400	-6.490524000
C	-1.941928700	19.621708500	-6.791800900
H	-0.809723700	22.872538200	-3.026882500
H	-0.934965200	22.144152300	-1.405286500
O	-3.854768700	20.980411900	-4.400484100
C	-2.745301200	23.109125200	-4.491937000
H	-1.542071800	18.617872500	-6.993266200
H	-2.174080200	20.112628900	-7.741350900
H	-2.869856500	19.527586100	-6.209182400
H	-1.826934300	23.129482400	-5.095740800
H	-2.675170300	23.870125800	-3.702721700
H	-3.586953600	23.344334200	-5.150025400
Au	4.861613200	9.054297600	14.088056400
P	6.220401000	9.766177900	15.759243300
C	5.512159700	9.632726800	17.479391700
C	7.915875700	9.015390500	15.850920300
C	6.746580800	11.544676000	15.829603100
N	6.338267500	10.230455200	18.515222300
H	4.519399700	10.116508600	17.421624400
H	5.346929100	8.568652300	17.674518000
N	8.762538900	9.573885600	16.897225300
H	7.828917800	7.934293800	16.013963500
H	8.367723800	9.184553300	14.858100900
N	7.589889600	11.745642400	17.008791400
H	7.300200000	11.796699700	14.913125500
H	5.862123800	12.195910500	15.878708800
C	6.854883400	11.587592900	18.256618700
C	6.652729700	9.609956600	19.715698500
C	8.866465600	11.040965200	16.924507200
C	9.142433000	8.738972200	17.940547900
H	6.008387200	12.294479900	18.229571300
H	7.498405900	11.837064900	19.107067700
O	7.370481000	10.170426300	20.546049400
C	6.098576800	8.220468600	19.957413200
H	9.483402300	11.335982400	17.777578400
H	9.363239200	11.379144200	15.999665200
O	8.735831100	7.578072600	18.001086800
C	10.095421500	9.302138100	18.975769600
H	5.006574500	8.176029700	19.837885300
H	6.359057900	7.941331800	20.982280900
H	6.563970800	7.500204300	19.268570700
H	9.565441300	9.983722600	19.656286900
H	10.944102700	9.831854600	18.521959500
H	10.467003800	8.458562000	19.565101100

Cellulose

C	-5.3843058	-0.1659404	-0.0083864
C	-3.9184826	-0.3881097	-0.3701347
C	-3.1936034	0.9553985	-0.4092104
C	-4.6955305	1.9139322	1.1868322
C	-5.5329641	0.6241376	1.2987204
H	-3.4414806	-1.0065786	0.4142739
H	-5.8483185	0.4075487	-0.8373569
H	-5.1288289	2.5722232	0.4024066

H	-5.0958994	0.0218246	2.1109585
H	-3.6181676	1.5942233	-1.2094662
O	-1.8293175	0.7367632	-0.6192264
C	-1.1909503	1.5158690	-1.6376748
C	-0.8884155	2.9394312	-1.1646319
C	0.0993538	0.7473296	-1.9867375
H	-1.8353536	1.5704263	-2.5344188
C	0.1256762	3.6324816	-2.0702421
H	-0.4579198	2.8698459	-0.1465164
H	0.6461189	0.5376801	-1.0450972
C	1.3446272	2.7406878	-2.2797636
H	-0.3308786	3.7969903	-3.0647812
H	1.8520859	2.5296657	-1.3084405
O	-3.3313069	1.6062252	0.8513410
O	0.9195458	1.5172369	-2.8731518
O	-3.7915692	-0.9978666	-1.6554077
H	-4.4334753	-1.7326547	-1.6583999
O	-5.9719031	-1.4824026	0.0509770
H	-6.9336114	-1.3939281	-0.0512239
C	-7.0021216	0.9097233	1.6167939
H	-7.5835080	-0.0227025	1.6492268
H	-7.1169089	1.3874223	2.5981707
H	-7.4532200	1.5703631	0.8588873
C	-4.6003651	2.7113120	2.4856499
H	-5.5957620	3.0520083	2.8008457
H	-3.9781538	3.6041231	2.2897117
O	-4.0719946	1.9363589	3.5609215
H	-3.2247507	1.5749600	3.2409836
O	-2.1159133	3.6761157	-1.1378716
H	-1.8808353	4.5880427	-0.8879320
O	0.4562137	4.8754572	-1.4414032
H	1.1220878	5.3050599	-2.0078778
O	2.2083866	3.4016745	-3.1548631
C	3.4725844	2.7407212	-3.3150268
H	3.3391187	1.7388249	-3.7486179
H	4.0661608	3.3649459	-3.9927530
H	3.9938750	2.6523664	-2.3450794
C	-0.2155123	-0.5713362	-2.6665677
H	-1.0532637	-1.0466659	-2.1296853
H	-0.5373674	-0.3539728	-3.7031229
O	0.9668366	-1.3819902	-2.6453913
H	0.7290250	-2.2581544	-2.9898893

PMMA

C	-0.6282290	0.3813165	0.0184793
C	0.0712682	1.2373720	-1.0436146
O	1.2759615	1.2643433	-1.2133381
O	-0.7920369	2.0031332	-1.7637018
C	-0.1667514	2.8656934	-2.7412867
H	0.3793408	2.2713229	-3.4858592
H	-0.9874855	3.4177358	-3.2103247
H	0.5369411	3.5531388	-2.2528041
C	-0.1802011	0.9466240	1.3963827
H	-0.6347918	0.3105599	2.1737979
H	0.9100290	0.8149382	1.4721011
C	-0.5334783	2.4146178	1.6480605
H	-0.1640806	2.7364855	2.6316129
H	-0.0752843	3.0706340	0.8930561
H	-1.6183764	2.5875935	1.6284594
C	-0.0948934	-1.0580169	-0.1239372
H	-0.5048403	-1.6870173	0.6795527

H	-0.3937982	-1.4970660	-1.0867973
H	1.0008061	-1.0749925	-0.0632861
C	-2.1580170	0.3844754	-0.1144226
H	-2.4709868	-0.0303950	-1.0819624
H	-2.5935529	-0.2371548	0.6818210
H	-2.5810969	1.3928351	-0.0366361

Polystyrene

C	-0.7425165	2.2785222	-0.2218198
C	0.2334171	2.6314094	-1.1678769
C	0.9652984	3.8132139	-1.0412023
C	0.7340087	4.6718537	0.0382347
C	-0.2327563	4.3344482	0.9878017
C	-0.9601423	3.1475474	0.8559129
H	0.4266336	1.9710751	-2.0156426
H	1.7195125	4.0664802	-1.7880703
H	1.3052776	5.5957638	0.1378269
H	-0.4211028	4.9948018	1.8359356
H	-1.7118918	2.8873654	1.6049466
C	-1.5407088	0.9930395	-0.3612591
H	-2.2025589	0.9252469	0.5204494
C	-0.6372416	-0.2575065	-0.3501818
H	-1.2773928	-1.1466259	-0.4748894
H	0.0225095	-0.2329705	-1.2341840
C	-2.4293323	1.0231528	-1.6162392
H	-3.0485529	0.1158832	-1.6787193
H	-3.0955368	1.8971333	-1.6082165
H	-1.8183205	1.0768065	-2.5295425
C	0.2025125	-0.3929770	0.9212662
H	0.8133781	-1.3064664	0.9022237
H	0.8787106	0.4657266	1.0416680
H	-0.4392758	-0.4360102	1.8146806

Zeonex

C	-1.8934317	0.1288415	0.1358556
C	-0.3471879	0.1267309	0.1550240
C	-0.8498694	2.3223790	-0.0529081
C	-2.2408347	1.6463784	-0.0242993
H	-2.2960490	-0.4884336	-0.6799928
H	-2.2938333	-0.2716474	1.0785278
H	-2.8158943	1.8556895	-0.9378199
H	-2.8347234	2.0103545	0.8266045
C	-0.0614406	1.4310677	0.9317433
H	1.0042247	1.6840985	1.0060859
H	-0.4893117	1.4276034	1.9455112
C	0.1483935	0.4603334	-1.2749212
C	-0.1803433	1.9943657	-1.4093824
H	-0.8923231	2.1854258	-2.2262019
H	0.0923127	-0.7921108	0.5657615
H	-0.8719853	3.3970220	0.1722055
C	1.6678603	0.2929408	-1.5321130
C	2.2736006	1.7067798	-1.3727122
C	1.1564810	2.7294938	-1.6924996
H	2.1005886	-0.3848809	-0.7752966
H	2.6235543	1.8478348	-0.3398209
H	3.1548064	1.8482766	-2.0180070
H	1.2476838	3.5974194	-1.0164593
H	-0.4196860	-0.1389841	-2.0021266
C	1.9573100	-0.3250715	-2.9130185
H	3.0326164	-0.2168206	-3.1314606
H	1.4289832	0.2547208	-3.6888069

C	1.5710920	-1.8027826	-3.0155276
H	0.5012679	-1.9599988	-2.8123142
H	1.7796678	-2.2041252	-4.0175852
H	2.1351244	-2.4050805	-2.2869916
C	1.2313948	3.2710972	-3.1323570
H	2.2659038	3.5935734	-3.3371278
H	1.0209825	2.4528814	-3.8418167
C	0.2796545	4.4415655	-3.3914795
H	0.3564290	4.8022939	-4.4272403
H	-0.7690993	4.1596611	-3.2157283
H	0.5086002	5.2854466	-2.7225353

Cellulose + L

C	-4.626481175	-0.950158747	0.843008613
C	-3.395902897	-1.191636697	-0.036054067
C	-2.502820384	0.058886936	-0.021607033
C	-3.324220999	0.768128057	2.080569319
C	-4.202560644	-0.488193339	2.243140077
H	-2.818044869	-2.034446293	0.371600706
H	-5.237133776	-0.153567724	0.386540473
H	-3.913013564	1.556258151	1.587139684
H	-3.602748429	-1.279169349	2.711938629
H	-3.035299645	0.879697488	-0.533125457
O	-1.314544818	-0.244859933	-0.668897135
C	-0.701612695	0.804919495	-1.388372562
C	-0.285428867	1.981451931	-0.489504668
C	0.516841367	0.154179903	-2.065591839
H	-1.397474487	1.193362061	-2.147580553
C	0.741397419	2.878543927	-1.193951022
H	0.165538823	1.580129266	0.430464038
H	1.085054421	-0.387252411	-1.295214826
C	1.885900192	2.038288499	-1.771230561
H	0.250223431	3.384562025	-2.039783435
H	2.409210369	1.494490575	-0.958569432
O	-2.192580203	0.440308892	1.298226588
O	1.349391202	1.110576520	-2.686220367
O	-3.770521654	-1.445897948	-1.362837191
H	-4.439252295	-2.143059058	-1.334800077
O	-5.368475503	-2.146409889	0.893236474
H	-6.072274675	-2.013998770	1.540785613
C	-2.785019478	1.299972896	3.413266393
H	-3.612660512	1.620484076	4.047788732
H	-2.143848736	2.164633777	3.196859550
O	-2.075379709	0.318724214	4.118752961
H	-1.394995162	-0.018259843	3.522418553
O	-1.426364304	2.742286765	-0.180929167
H	-1.123725083	3.513615910	0.315321322
O	1.203790310	3.818279676	-0.256968204
H	1.900821859	4.335113397	-0.679235189
O	2.742206926	2.907940331	-2.423117977
C	0.085366663	-0.848389756	-3.133692131
H	-0.771030185	-1.411513528	-2.743549865
H	-0.215584413	-0.292217162	-4.030613467
O	1.177530877	-1.693684983	-3.402951036
H	0.950978156	-2.277922106	-4.133862211
C	4.013122100	2.082747773	-4.335317441
C	5.449213736	1.855930609	-4.826073082
C	6.362208849	3.018511392	-4.418430580
C	5.038548896	3.486668140	-2.495411299
C	4.009383581	2.389790135	-2.833351902
H	5.838752063	0.938100440	-4.358444095

H	3.591602143	2.958878380	-4.858442696
H	4.650802113	4.441257414	-2.883806913
H	4.248580184	1.477940320	-2.271740917
H	6.054550205	3.933594573	-4.949874417
O	7.667299360	2.663456707	-4.737655230
C	8.499352445	3.652014923	-5.307757283
C	8.848576513	4.777853009	-4.324515411
C	9.779680319	2.900981526	-5.707333715
H	8.015716090	4.087299792	-6.193431035
C	10.018236637	5.622943273	-4.848053456
H	9.157204057	4.317823729	-3.374935393
H	10.171882779	2.417856876	-4.797143483
C	11.190391520	4.737570870	-5.287066072
H	9.687905144	6.196380081	-5.728383356
H	11.615675931	4.226597829	-4.397975298
O	6.314305317	3.219053028	-3.025832220
O	10.758625622	3.778006653	-6.224062625
O	5.476602740	1.743725344	-6.222782837
H	4.779477433	1.116555311	-6.458825434
O	3.296472204	0.925607812	-4.681238578
H	2.508076517	0.853415186	-4.117339193
C	5.233865239	3.566024974	-0.969292476
H	4.262532904	3.556283352	-0.469756455
H	5.751627502	4.500876104	-0.718781050
O	5.952112967	2.460921888	-0.501670669
H	6.742732351	2.396641579	-1.062336920
O	7.725491648	5.598044508	-4.125611673
H	8.010125017	6.335552324	-3.568705925
O	10.406527002	6.488599289	-3.809719914
H	11.277144696	6.848933699	-4.022200084
O	12.124996880	5.570035951	-5.881835992
C	9.514379667	1.823898546	-6.770979146
H	8.520042202	1.400227116	-6.596171571
H	9.541441458	2.297685324	-7.754690619
O	10.508241805	0.837783643	-6.778227909
H	10.409206763	0.289464938	-5.985487722
C	13.803774619	3.977721201	-6.790212318
C	15.326409226	3.788016653	-6.899775103
C	16.022751095	5.110945288	-7.239012452
C	14.300800014	6.385012801	-6.280773319
C	13.483025785	5.153076253	-5.849351993
H	15.709083319	3.441117940	-5.928009364
H	13.402350803	4.218186316	-7.786400481
H	13.971950119	6.682938536	-7.287963785
H	13.754849931	4.867854995	-4.821841629
H	15.704645462	5.439859184	-8.243661310
O	17.393479433	4.920809230	-7.187692955
C	18.166478713	5.665508510	-8.107446342
C	18.078044138	7.181250959	-7.869407402
C	19.607933361	5.153087257	-7.925969904
H	17.821955614	5.465074779	-9.133323660
C	19.220196145	7.914971393	-8.582887571
H	18.152355335	7.372236963	-6.788096785
H	19.854989497	5.211841385	-6.854989190
C	20.566959002	7.253010968	-8.272535950
H	19.064841960	7.842249162	-9.670867198
H	20.771039574	7.307171136	-7.179729366
O	15.676557519	6.092092522	-6.286044446
O	20.531206982	5.907795494	-8.673584658
O	15.620559078	2.855485976	-7.903965977
H	15.041729213	2.096203883	-7.746293910

O	13.307832567	2.740094982	-6.343335709
H	12.345109425	2.720102450	-6.475957402
C	14.099686613	7.563007262	-5.318598016
H	13.058266251	7.886048323	-5.352715259
H	14.745974615	8.384655173	-5.653684641
O	14.380956976	7.215295826	-3.991410236
H	15.274415319	6.849261198	-3.978796864
O	16.846502658	7.652751673	-8.360889209
H	16.868682951	8.615416913	-8.287372113
O	19.193218206	9.260391025	-8.171570046
H	19.993221671	9.680384410	-8.510480316
O	21.537601011	7.941041538	-8.984939162
C	22.856576329	7.476647964	-8.751987732
H	22.945979880	6.422713634	-9.019081220
H	23.502039835	8.082114394	-9.383701705
H	23.129679954	7.609218612	-7.699381670
C	19.723233444	3.693291547	-8.361213573
H	18.797258427	3.180842390	-8.075273350
H	19.841828589	3.668666347	-9.451854997
O	20.839768699	3.120709919	-7.720584864
H	20.914614243	2.201228751	-7.996968704
O	-5.373959371	-0.245799772	2.997264346
C	-5.344253422	-0.753099535	4.317278082
H	-6.255833778	-0.397882453	4.794140594
H	-5.336419962	-1.849388553	4.316189986
H	-4.473853012	-0.395616038	4.871371770
C	9.167450099	3.730157160	-0.629611351
C	8.529941977	4.936086038	-0.856541961
H	7.757059017	4.996716811	-1.604407239
C	8.899923008	6.069868059	-0.138034969
C	9.917750406	5.976321972	0.808287573
H	10.239269577	6.834298399	1.375922906
C	10.535572419	4.759789486	1.036779481
H	11.322159093	4.690927736	1.772475431
C	10.173027220	3.627516865	0.328891178
C	10.776879773	2.268329299	0.595653348
C	10.738627784	1.450568282	-0.675775137
C	11.664427101	0.460158031	-0.952696505
H	12.471263180	0.283070591	-0.258328252
C	11.585680575	-0.289752386	-2.112854186
H	12.339985960	-1.036621983	-2.298762711
C	10.547264680	-0.064995792	-3.012412182
C	9.597067564	0.915167807	-2.736394654
H	8.787519933	1.108779790	-3.422252000
C	9.707611307	1.677630593	-1.585915412
C	10.027312196	1.569181328	1.707947590
C	8.697359589	1.217803851	1.815282071
H	7.984114090	1.434042141	1.034829187
C	8.298891917	0.579516034	2.981521055
H	7.263907851	0.294343723	3.094468042
C	9.199156524	0.304172347	4.003911571
H	8.851819043	-0.194648360	4.896197588
C	10.531596192	0.665611788	3.894999966
H	11.244677282	0.466499184	4.679295363
C	10.928873346	1.302892769	2.731877216
C	12.248719370	1.822143331	2.319048675
O	8.212737732	7.196967739	-0.444237620
O	8.784015004	2.668402948	-1.406338802
O	10.384344693	-0.718828367	-4.194817016
O	12.113048278	2.377570308	1.091665156
O	13.293030307	1.798453704	2.908443963

C	11.272907275	-1.785607039	-4.510445883
H	12.296786195	-1.404323604	-4.638238726
H	11.261473243	-2.540500676	-3.711150826
C	8.584288236	8.410277199	0.191157447
H	8.451958329	8.331794231	1.281366745
H	9.635177519	8.651809289	-0.029033104
C	7.734287276	9.473403677	-0.312364964
C	7.023057100	10.335838577	-0.737761339
C	10.831461596	-2.394699569	-5.751487412
C	10.440953037	-2.884382665	-6.770168288
H	10.117249700	-3.316190566	-7.669566406
H	6.406536837	11.103623219	-1.099354206

Cellulose + 1

C	-4.708900000	-0.440000000	0.672400000
C	-3.524400000	-0.772500000	-0.239200000
C	-2.439000000	0.304200000	-0.082000000
C	-3.158000000	0.880800000	2.096200000
C	-4.229800000	-0.227100000	2.114000000
H	-3.097000000	-1.741300000	0.059300000
H	-5.174900000	0.495500000	0.321100000
H	-3.605700000	1.807200000	1.705500000
H	-3.769800000	-1.155700000	2.476400000
H	-2.824500000	1.256000000	-0.487400000
O	-1.309000000	-0.110100000	-0.771100000
C	-0.538100000	0.902100000	-1.384500000
C	0.072100000	1.887700000	-0.375100000
C	0.557500000	0.148500000	-2.156300000
H	-1.166000000	1.478800000	-2.080700000
C	1.211500000	2.692100000	-1.015600000
H	0.472700000	1.318200000	0.476900000
H	1.046900000	-0.548400000	-1.460200000
C	2.208100000	1.765100000	-1.722900000
H	0.787200000	3.356400000	-1.785000000
H	2.670400000	1.068600000	-0.994000000
O	-2.085800000	0.469500000	1.270900000
O	1.516400000	1.026000000	-2.705500000
O	-3.920200000	-0.801400000	-1.584100000
H	-4.695200000	-1.376700000	-1.633600000
O	-5.638700000	-1.494500000	0.583900000
H	-6.314700000	-1.329700000	1.253400000
C	-2.555600000	1.155400000	3.478800000
H	-3.328200000	1.528400000	4.152900000
H	-1.780200000	1.923800000	3.362100000
O	-2.023800000	-0.005100000	4.057000000
H	-1.394700000	-0.371600000	3.422900000
O	-0.928300000	2.774500000	0.059700000
H	-0.494500000	3.431700000	0.619200000
O	1.825300000	3.443200000	0.000300000
H	2.591000000	3.887000000	-0.384700000
O	3.160100000	2.574900000	-2.314200000
C	-0.036000000	-0.660900000	-3.307200000
H	-0.941200000	-1.159000000	-2.940600000
H	-0.297900000	0.029800000	-4.119000000
O	0.934200000	-1.590300000	-3.725300000
H	0.602200000	-2.073500000	-4.488800000
C	4.241800000	1.509300000	-4.244500000
C	5.623000000	1.289500000	-4.880400000
C	6.532800000	2.496000000	-4.639100000
C	5.442200000	3.050200000	-2.611600000
C	4.363600000	1.959800000	-2.778900000

H	6.094200000	0.412700000	-4.411800000
H	3.725000000	2.314000000	-4.795500000
H	5.031100000	3.989700000	-3.011700000
H	4.613900000	1.093100000	-2.152100000
H	6.116800000	3.383500000	-5.144100000
O	7.799500000	2.191400000	-5.115900000
C	8.533400000	3.238800000	-5.714800000
C	8.841100000	4.395000000	-4.748700000
C	9.824400000	2.566100000	-6.207800000
H	7.969900000	3.650300000	-6.565900000
C	9.976100000	5.283300000	-5.283600000
H	9.149500000	3.985000000	-3.776700000
H	10.278600000	2.036000000	-5.355500000
C	11.167000000	4.446500000	-5.762900000
H	9.608100000	5.853600000	-6.152300000
H	11.621200000	3.917800000	-4.900400000
O	6.649300000	2.729100000	-3.255600000
O	10.741300000	3.507700000	-6.725400000
O	5.484400000	1.092800000	-6.262300000
H	4.811900000	0.405600000	-6.365500000
O	3.567800000	0.289200000	-4.410600000
H	2.715600000	0.319700000	-3.942700000
C	5.807200000	3.214600000	-1.127100000
H	4.903800000	3.330600000	-0.525300000
H	6.437300000	4.104800000	-1.013300000
O	6.473600000	2.076500000	-0.654900000
H	7.188200000	1.908400000	-1.286500000
O	7.680100000	5.175500000	-4.590600000
H	7.922900000	5.920700000	-4.025900000
O	10.347500000	6.152100000	-4.244600000
H	11.225900000	6.506900000	-4.431000000
O	12.068700000	5.320800000	-6.350400000
C	9.533600000	1.552800000	-7.319900000
H	8.751200000	0.873900000	-6.964500000
H	9.183500000	2.088500000	-8.205200000
O	10.689100000	0.856900000	-7.709700000
H	11.021500000	0.355500000	-6.947100000
C	13.782800000	3.858700000	-7.399800000
C	15.309300000	3.699700000	-7.502800000
C	15.990800000	5.054700000	-7.721700000
C	14.228000000	6.217100000	-6.689900000
C	13.436600000	4.937800000	-6.358300000
H	15.684300000	3.279800000	-6.557500000
H	13.392600000	4.189800000	-8.375500000
H	13.902100000	6.581500000	-7.676100000
H	13.722500000	4.573900000	-5.360300000
H	15.691300000	5.457500000	-8.705200000
O	17.362500000	4.877600000	-7.651300000
C	18.151200000	5.729600000	-8.457900000
C	17.989800000	7.214000000	-8.094000000
C	19.599600000	5.251600000	-8.239300000
H	17.868400000	5.610300000	-9.515000000
C	19.145400000	8.049300000	-8.659000000
H	17.985200000	7.308400000	-6.997700000
H	19.781500000	5.215800000	-7.154300000
C	20.494200000	7.404500000	-8.323800000
H	19.061500000	8.076300000	-9.757000000
H	20.625700000	7.357000000	-7.219600000
O	15.610200000	5.953900000	-6.703200000
O	20.534600000	6.104900000	-8.854100000
O	15.628200000	2.854500000	-8.575200000

H	15.080900000	2.064400000	-8.462500000
O	13.298400000	2.578300000	-7.086000000
H	12.328500000	2.579200000	-7.169500000
C	13.993600000	7.317300000	-5.646400000
H	12.948600000	7.629100000	-5.676000000
H	14.633700000	8.170100000	-5.908200000
O	14.254800000	6.876200000	-4.344200000
H	15.156300000	6.530900000	-4.334700000
O	16.775400000	7.687000000	-8.626100000
H	16.749800000	8.637300000	-8.456400000
O	19.040700000	9.347500000	-8.126300000
H	19.846500000	9.821900000	-8.365200000
O	21.481800000	8.189600000	-8.899400000
C	22.799500000	7.749100000	-8.617400000
H	22.947900000	6.728400000	-8.972700000
H	23.461800000	8.432100000	-9.143800000
H	22.995600000	7.791100000	-7.540400000
C	19.797400000	3.844600000	-8.800200000
H	18.882500000	3.272700000	-8.606800000
H	19.965500000	3.925900000	-9.881700000
O	20.908000000	3.259300000	-8.161300000
H	21.029000000	2.369000000	-8.507900000
O	-5.354000000	0.106500000	2.904200000
C	-5.417200000	-0.555800000	4.152500000
H	-6.264100000	-0.120100000	4.679200000
H	-5.585100000	-1.630800000	4.018000000
H	-4.505500000	-0.412400000	4.736000000
C	9.267900000	4.210000000	-0.222100000
C	8.721100000	5.474100000	-0.371600000
H	8.260100000	5.750100000	-1.305700000
C	8.776000000	6.401500000	0.667100000
C	9.402900000	6.040100000	1.860500000
H	9.471700000	6.727800000	2.687400000
C	9.949300000	4.779300000	1.995100000
H	10.437500000	4.510200000	2.920200000
C	9.892900000	3.849200000	0.969100000
C	10.457400000	2.461400000	1.155900000
C	10.543700000	1.728500000	-0.160200000
C	11.260700000	0.547200000	-0.275200000
H	11.801500000	0.178100000	0.583800000
C	11.316000000	-0.156100000	-1.463600000
H	11.901700000	-1.059900000	-1.513400000
C	10.604600000	0.306800000	-2.569800000
C	9.852600000	1.473400000	-2.454400000
H	9.261400000	1.804800000	-3.292100000
C	9.850500000	2.197700000	-1.274000000
C	9.635000000	1.682500000	2.154300000
C	8.308600000	1.303400000	2.116700000
H	7.672400000	1.552200000	1.280600000
C	7.821200000	0.588500000	3.201600000
H	6.787400000	0.276800000	3.203200000
C	8.631400000	0.265800000	4.284300000
H	8.215500000	-0.291600000	5.110600000
C	9.961200000	0.650300000	4.317500000
H	10.605600000	0.411300000	5.148900000
C	10.447900000	1.363400000	3.235200000
C	11.793400000	1.915300000	2.966000000
O	8.200800000	7.594300000	0.419900000
O	9.147900000	3.366500000	-1.286300000
O	10.582100000	-0.264300000	-3.790200000
O	11.759200000	2.534700000	1.768300000

O	12.781900000	1.859100000	3.645800000
C	11.309000000	-1.470600000	-4.025400000
H	12.375300000	-1.323300000	-3.794000000
H	10.913600000	-2.281200000	-3.395100000
C	8.242400000	8.593400000	1.435900000
H	7.717300000	8.233800000	2.335400000
H	9.288400000	8.815600000	1.699400000
C	7.600600000	9.806300000	0.949800000
C	7.074400000	10.818800000	0.564700000
C	11.139200000	-1.783700000	-5.437600000
C	10.964000000	-2.002000000	-6.610200000
Au	6.263600000	12.486000000	-0.040500000
P	5.362600000	14.451500000	-0.717400000
N	4.418800000	16.967600000	-0.049800000
N	3.365200000	15.907900000	-1.964100000
N	5.614300000	16.802900000	-2.157100000
C	4.949800000	15.760900000	0.545200000
H	4.214500000	15.352200000	1.249500000
H	5.859800000	16.005700000	1.107200000
C	3.744300000	14.549600000	-1.643100000
H	3.833400000	13.970900000	-2.570900000
H	2.958800000	14.091200000	-1.029500000
C	6.316200000	15.572900000	-1.863100000
H	7.281600000	15.810000000	-1.398700000
H	6.511800000	15.035900000	-2.799600000
C	4.345600000	16.554000000	-2.822200000
H	4.518400000	15.929700000	-3.702900000
H	3.935000000	17.516600000	-3.141500000
C	5.372700000	17.586500000	-0.956300000
H	6.319300000	17.739600000	-0.431200000
H	4.971300000	18.558100000	-1.259900000
C	3.180300000	16.714300000	-0.768200000
H	2.475300000	16.210700000	-0.101300000
H	2.759900000	17.678200000	-1.070400000
Au	10.583100000	-1.944500000	-8.528700000
P	10.113400000	-1.510900000	-10.702200000
N	9.116400000	0.545600000	-12.261700000
N	8.796800000	-1.702300000	-13.128500000
N	10.999300000	-0.677200000	-13.188200000
C	9.332300000	0.165200000	-10.881900000
H	8.372800000	0.164900000	-10.350700000
H	9.989500000	0.894600000	-10.394400000
C	8.960400000	-2.404400000	-11.874000000
H	9.355000000	-3.408200000	-12.073500000
H	7.981200000	-2.511300000	-11.391300000
C	11.480500000	-1.226900000	-11.938900000
H	12.205500000	-0.534300000	-11.494200000
H	11.991400000	-2.177200000	-12.138100000
C	10.057200000	-1.570400000	-13.842200000
H	10.513800000	-2.558300000	-13.948300000
H	9.842200000	-1.168000000	-14.837000000
C	10.367200000	0.619400000	-12.998200000
H	11.057200000	1.279400000	-12.466300000
H	10.156800000	1.040800000	-13.986200000
C	8.222400000	-0.378800000	-12.938400000
H	7.297600000	-0.469500000	-12.362500000
H	7.989900000	0.032500000	-13.925700000

Cellulose + 2

C	-4.673546093	-0.565813743	0.763694021
C	-3.473857854	-0.859194557	-0.141429890

C	-2.445013492	0.275605462	-0.014193952
C	-3.192369644	0.870946776	2.149204439
C	-4.205470445	-0.290013169	2.198218086
H	-2.996429056	-1.796441017	0.180953429
H	-5.188814909	0.334209084	0.389051269
H	-3.687800649	1.762275384	1.735059526
H	-3.698278188	-1.183632051	2.584634353
H	-2.878497263	1.195737350	-0.443824096
O	-1.296339346	-0.099375127	-0.694884859
C	-0.576261261	0.932632266	-1.335853575
C	0.001250248	1.961356044	-0.350007022
C	0.540136686	0.210851744	-2.108401325
H	-1.235769186	1.470045303	-2.034199220
C	1.107044551	2.797379760	-1.009801365
H	0.427099262	1.424637269	0.510965428
H	1.063038996	-0.456882005	-1.408263553
C	2.130511704	1.894675414	-1.709761766
H	0.654106178	3.434735783	-1.785561896
H	2.619422944	1.224688406	-0.973615510
O	-2.100356065	0.494381363	1.333149892
O	1.458854630	1.118786798	-2.677275755
O	-3.868062062	-0.943127305	-1.484461418
H	-4.611535810	-1.559714233	-1.517924720
O	-5.545969551	-1.670170476	0.704666323
H	-6.229943901	-1.523972328	1.370316355
C	-2.604633226	1.212841634	3.523042929
H	-3.395392289	1.562698296	4.188464498
H	-1.870361224	2.017335587	3.384958452
O	-2.012738462	0.097279444	4.130595050
H	-1.366471124	-0.253161305	3.504743818
O	-1.032445486	2.815710883	0.071039347
H	-0.626989798	3.493838320	0.626567612
O	1.700482014	3.584221801	-0.008893410
H	2.444102376	4.054048525	-0.405780715
O	3.053223018	2.724971849	-2.320227198
C	-0.033008621	-0.637214235	-3.241672645
H	-0.922669709	-1.153448087	-2.861971438
H	-0.317179592	0.029942337	-4.065676439
O	0.960784790	-1.547548884	-3.645733998
H	0.639264773	-2.053122585	-4.399196687
C	4.144020716	1.782793233	-4.300993989
C	5.528974656	1.550133785	-4.922632513
C	6.473541895	2.710425504	-4.599085198
C	5.352071254	3.191467301	-2.568225604
C	4.257969288	2.130412220	-2.808431774
H	5.961302945	0.633565211	-4.494149141
H	3.671288808	2.644184696	-4.805012098
H	4.967473462	4.156574912	-2.932531373
H	4.493645856	1.222693974	-2.237568075
H	6.102263437	3.635397908	-5.070757572
O	7.740361814	2.381984304	-5.062209444
C	8.518529335	3.423496434	-5.612461298
C	8.875212351	4.518055210	-4.593296974
C	9.781451478	2.722648946	-6.138273284
H	7.974000109	3.898103503	-6.442841801
C	10.038423748	5.388655157	-5.095459812
H	9.174833736	4.047885134	-3.646474078
H	10.217719988	2.141440301	-5.310354711
C	11.195191667	4.536460358	-5.628818089
H	9.683776900	6.014842554	-5.930607004
H	11.647516052	3.960758346	-4.796067603

O	6.564984724	2.874205009	-3.204541871
O	10.730214661	3.650064230	-6.622713673
O	5.412942412	1.431621319	-6.316197868
H	4.705369716	0.789670350	-6.466710057
O	3.419815269	0.609072542	-4.562870300
H	2.608192886	0.600402169	-4.026742815
C	5.688914733	3.281642954	-1.070104068
H	4.773328142	3.372602073	-0.482447093
H	6.318830728	4.163141441	-0.900432246
O	6.342307011	2.120539956	-0.639438126
H	7.077764565	1.989039020	-1.255734481
O	7.745665545	5.332116660	-4.386891942
H	8.018703653	6.035724256	-3.783814953
O	10.452080429	6.189783994	-4.018722457
H	11.335014141	6.533964380	-4.204271231
O	12.110631426	5.409280004	-6.196852350
C	9.449081190	1.769088186	-7.290883560
H	8.630255004	1.117761140	-6.966563097
H	9.135248202	2.358630523	-8.155494664
O	10.569357837	1.030645941	-7.704478396
H	10.883328642	0.495928395	-6.956454997
C	13.771219991	3.939843060	-7.320340231
C	15.291828043	3.747474936	-7.451186173
C	15.998702741	5.092894807	-7.648905547
C	14.282219270	6.265411970	-6.551897157
C	13.468405925	4.994794154	-6.241629762
H	15.672270621	3.296494641	-6.522627035
H	13.375670320	4.310418452	-8.279580566
H	13.946102888	6.664693602	-7.521079178
H	13.763920607	4.595483378	-5.260131153
H	15.687864317	5.527058335	-8.615161354
O	17.367675878	4.887875138	-7.610618884
C	18.155882591	5.743165120	-8.414756599
C	18.014565373	7.224637811	-8.030929435
C	19.602475492	5.250312705	-8.217632939
H	17.860491053	5.639523410	-9.470057531
C	19.168112716	8.053891258	-8.608516245
H	18.031004043	7.306682195	-6.933696106
H	19.797507568	5.204954269	-7.135283240
C	20.516475297	7.395766015	-8.298379413
H	19.067526560	8.088531998	-9.704928751
H	20.664600371	7.339529874	-7.196732260
O	15.657434184	5.972691428	-6.599543171
O	20.537403019	6.099493298	-8.838157001
O	15.573378942	2.921386869	-8.548622124
H	15.009914996	2.141511021	-8.444660756
O	13.257639504	2.662927818	-7.039046936
H	12.287054611	2.693945995	-7.105478292
C	14.090119110	7.340062068	-5.473644594
H	13.052263155	7.676136422	-5.477850117
H	14.745943530	8.185052244	-5.721133572
O	14.360112459	6.855681602	-4.188633362
H	15.254501576	6.492456371	-4.201785371
O	16.795813903	7.717385515	-8.534750548
H	16.790141448	8.667844002	-8.363672359
O	19.081941570	9.349564900	-8.066308257
H	19.887680419	9.819057226	-8.315075414
O	21.501223460	8.176694613	-8.884116182
C	22.819767685	7.724056387	-8.625990475
H	22.954319620	6.704373718	-8.989676027
H	23.478891240	8.404973577	-9.159034964

H	23.033501298	7.758086498	-7.552075928
C	19.782026550	3.845773130	-8.790874760
H	18.863605596	3.280598618	-8.593766203
H	19.941438251	3.933868621	-9.873135406
O	20.892564621	3.245931912	-8.165757833
H	21.007603832	2.359835906	-8.525045256
O	-5.345205919	0.006553442	2.980876892
C	-5.374798501	-0.624465860	4.246525523
H	-6.243421966	-0.219058506	4.761951453
H	-5.486839775	-1.709933213	4.141070060
H	-4.472050500	-0.418522739	4.825277097
C	9.229015204	4.167698378	-0.153244382
C	8.688566344	5.436685492	-0.278957841
H	8.175732700	5.712077130	-1.185765130
C	8.815454001	6.368968585	0.749050152
C	9.504951706	6.006372194	1.906700647
H	9.628901972	6.697174913	2.724502129
C	10.042323032	4.739194238	2.018728411
H	10.578700527	4.468649408	2.916155560
C	9.916772438	3.805731435	1.002678063
C	10.464795338	2.407671648	1.160056421
C	10.541162623	1.707952722	-0.175155087
C	11.297414036	0.558603448	-0.343834049
H	11.883908014	0.191171869	0.485120230
C	11.333564554	-0.114052361	-1.550769835
H	11.950351972	-0.993301506	-1.644315325
C	10.564382037	0.346651055	-2.618088917
C	9.774318722	1.481403026	-2.448876313
H	9.143813284	1.813389512	-3.257650047
C	9.789591718	2.175668633	-1.250954887
C	9.625387390	1.611719857	2.131558724
C	8.293130619	1.255643183	2.079894393
H	7.659875231	1.543291587	1.254123001
C	7.793039564	0.510562518	3.138544968
H	6.754324613	0.216060851	3.127974131
C	8.596666007	0.136315505	4.209320858
H	8.171177786	-0.444105532	5.014461753
C	9.932264285	0.498752195	4.257372399
H	10.571869944	0.220010692	5.080061554
C	10.431540050	1.242275276	3.201563979
C	11.785282730	1.782988743	2.954435993
O	8.245004627	7.569616062	0.526439476
O	9.046454145	3.319523607	-1.205928663
O	10.520056296	-0.197379434	-3.850957683
O	11.762465356	2.445444957	1.778567890
O	12.771833125	1.689770502	3.632606308
C	11.274012900	-1.375987028	-4.135730990
H	12.343797739	-1.199398713	-3.944928033
H	10.929385584	-2.209637002	-3.506152696
C	8.374528198	8.577430768	1.525859377
H	7.892579061	8.243774962	2.458764542
H	9.440094975	8.771843626	1.725081725
C	7.741451992	9.804322290	1.064688825
C	7.224569651	10.829325926	0.700798786
C	11.055683069	-1.666689086	-5.545643214
C	10.832545840	-1.856220112	-6.715001308
Au	6.432281725	12.519755049	0.139215938
Au	10.379350895	-1.740652637	-8.613821053
P	5.579935584	14.520702977	-0.495843412
O	2.565271234	17.538469672	-2.826952122
O	2.216454320	16.384562880	0.797036421

N	6.004549605	17.031183207	-1.447332644
N	4.348555802	16.880341229	0.314779413
N	4.027156912	15.918456867	-2.325082117
C	4.386998525	14.585476057	-1.926463658
H	4.879900877	14.053300143	-2.758187216
H	3.504959121	14.012796569	-1.631078660
C	4.760046374	15.577502928	0.782752534
H	3.872294366	15.061381483	1.168457755
H	5.486970062	15.682675900	1.604475693
C	6.736953978	15.836329805	-1.096783542
H	7.462015223	16.064637918	-0.306881355
H	7.287791815	15.475969580	-1.972655642
C	5.139921861	16.824094372	-2.579027907
H	5.752101184	16.423138585	-3.397332682
H	4.705894369	17.780120589	-2.883593870
C	5.385701871	17.676076375	-0.313519994
H	4.962741825	18.618699694	-0.658095583
H	6.179319848	17.878944528	0.419846661
C	2.753254063	16.380656518	-2.507131823
C	1.621433962	15.411859193	-2.292303449
H	1.753418599	14.500687277	-2.870795046
H	0.703906211	15.903821829	-2.604278749
H	1.535390901	15.171042279	-1.233611696
C	3.025961387	17.215562174	0.436086228
C	2.643828598	18.640146363	0.136779375
H	2.652159307	18.812946453	-0.938191416
H	3.307047322	19.345590652	0.630566125
H	1.627924072	18.788579950	0.493602368
P	9.836437646	-1.237900468	-10.753572812
O	10.358314057	-0.676242255	-15.549158840
O	7.872367191	-3.050545721	-13.829854954
N	9.305764915	0.906569789	-12.333401644
N	7.965133262	-1.059134132	-12.804119462
N	10.729894985	-0.787513363	-13.343432295
C	11.076608891	-1.470256381	-12.127210510
H	12.033443637	-1.088686790	-11.732351946
H	11.179897899	-2.544135948	-12.295916428
C	8.230078276	-1.716548511	-11.545058956
H	8.207343068	-2.798837100	-11.720104934
H	7.445789234	-1.462884015	-10.813736253
C	9.596014528	0.580379944	-10.955249202
H	8.766587193	0.897452624	-10.313751553
H	10.496130201	1.107912473	-10.623738477
C	10.419079838	0.629798109	-13.201495511
H	11.295485149	1.150994724	-12.795808993
H	10.200966119	1.006301537	-14.204528056
C	8.035369815	0.390976502	-12.782896710
H	7.855826967	0.776644619	-13.785604736
H	7.266343584	0.777890284	-12.099076721
C	10.686289275	-1.346573654	-14.588386886
C	11.049076929	-2.802888827	-14.713918246
H	12.016995815	-3.018276350	-14.267728344
H	11.082370547	-3.043626755	-15.773244710
H	10.280096853	-3.418016810	-14.248824375
C	7.727449861	-1.845447763	-13.898144507
C	7.258415997	-1.160906077	-15.154580883
H	8.085922442	-0.632272971	-15.624756199
H	6.443500466	-0.470154331	-14.954182198
H	6.917738184	-1.932421970	-15.840227891

PMMA + L

C	0.689999720	0.391860134	-1.188969350
C	0.214771970	1.575593277	-2.024533344
O	0.439007891	1.738625828	-3.193714554
O	-0.492159341	2.452341604	-1.293393590
C	-0.973748153	3.588301899	-1.995884423
H	-1.659523333	3.280802873	-2.787967881
H	-1.487709511	4.205915703	-1.263403483
H	-0.143316524	4.135076253	-2.447315810
C	1.497775576	0.936043109	0.023646797
H	0.904767784	1.742335646	0.460436447
H	1.563870249	0.136841801	0.764838259
C	2.939398013	1.468909709	-0.184295600
C	1.489410076	-0.552962870	-2.080617326
H	1.811427328	-1.424142919	-1.515059705
H	0.880578909	-0.873380425	-2.920253929
H	2.361270896	-0.063376185	-2.499880986
C	-0.520926519	-0.332123424	-0.543163653
H	-0.107216812	-1.117385275	0.093496753
H	-1.020015270	0.395813283	0.100748700
C	3.354488932	2.093254598	1.171192469
H	3.339873066	1.289408124	1.909521968
H	2.585781784	2.817150390	1.449996260
C	-1.625884914	-0.995423059	-1.407744709
C	4.724570736	2.814741948	1.295654726
C	4.870487946	3.088644482	2.817185663
H	4.832067280	2.115222277	3.310838490
H	3.989130721	3.657479715	3.123695455
C	6.103621714	3.829482731	3.395021412
C	5.863988996	3.826371735	4.927510638
H	5.907712579	2.787585415	5.261553367
H	4.849911558	4.191525737	5.101915560
C	6.796269603	4.639972235	5.843170761
C	6.382656631	4.345602569	7.298323725
H	6.558524124	3.297343898	7.528464907
H	6.968029173	4.951606181	7.986469835
H	5.328654875	4.566202088	7.450339804
C	3.022320700	2.484889624	-1.318008143
H	2.717060895	2.050082859	-2.263792739
H	2.379238430	3.334743873	-1.098833759
H	4.042334227	2.833202393	-1.447254702
C	5.884227399	1.981846271	0.761124449
H	5.978399070	1.056552779	1.322758273
H	5.748061934	1.747080218	-0.289436022
H	6.812686667	2.539636435	0.834920079
C	6.234022461	5.246061587	2.844240180
H	6.500593040	5.218412021	1.792298088
H	5.299022687	5.786471402	2.971843739
H	7.028123956	5.783124575	3.355751657
C	8.274616767	4.297914443	5.672885901
H	8.428670294	3.230570484	5.811150791
H	8.638135116	4.585498922	4.691608705
H	8.862814176	4.836068921	6.412384517
C	3.833829007	0.269081858	-0.469724778
O	4.451626289	0.090174186	-1.484883233
O	3.834414867	-0.601483256	0.544544120
C	4.674614997	4.135766467	0.541910115
O	5.483294539	4.502984422	-0.273140368
O	3.618987185	4.880535435	0.889729704
C	7.384615166	3.054981178	3.085404824
O	8.333342819	3.510203006	2.500327148
O	7.337959317	1.811615128	3.566199768

C	6.601728969	6.142919907	5.683077714
O	7.482456325	6.961297603	5.708374418
O	5.304082203	6.475818061	5.595480516
C	4.646476571	-1.755145671	0.371039172
H	4.559500769	-2.328439292	1.290701810
H	4.297741463	-2.338218519	-0.484417975
H	5.681465465	-1.461490476	0.191465065
C	3.500423789	6.135523408	0.234497368
H	3.412594729	5.998988348	-0.845804457
H	2.604300825	6.603514332	0.633979623
H	4.377176504	6.754171193	0.436843401
C	8.483088253	0.992202655	3.344898232
H	9.390090308	1.596065106	3.398776403
H	8.477418516	0.236086770	4.126710583
H	8.430646795	0.514925177	2.364410376
C	5.028894885	7.865032810	5.502985287
H	3.946525660	7.960645585	5.466535481
H	5.431873680	8.391344588	6.370487021
H	5.482255223	8.281311792	4.600834684
C	-2.568528220	-1.652010030	-0.364381109
H	-1.985206953	-2.427155234	0.136938711
H	-2.815560333	-0.888777954	0.376674478
C	-3.908223203	-2.300002116	-0.798275969
C	-4.453738949	-2.958708246	0.496143799
H	-3.663469455	-3.600130393	0.890974769
H	-4.629809669	-2.160084372	1.220305668
C	-5.732612583	-3.814140316	0.437792063
C	-2.343855498	0.022449002	-2.287148823
H	-2.745043465	0.828325884	-1.676706235
H	-1.658186545	0.432259952	-3.022620279
H	-3.151522091	-0.446713888	-2.839812391
C	-3.731513845	-3.319259596	-1.918341243
H	-3.385024997	-2.837597663	-2.827563493
H	-3.015177351	-4.080430483	-1.619594209
H	-4.680895463	-3.794454836	-2.151139571
C	-6.060584602	-4.245796303	1.880245595
H	-6.295855190	-3.372559456	2.484443816
H	-6.921117685	-4.911202311	1.888063135
H	-5.215976975	-4.764547523	2.327409024
C	-6.937940961	-3.077808316	-0.142219180
H	-7.099694312	-2.146311026	0.395110845
H	-6.801100284	-2.865434394	-1.197637947
H	-7.826877695	-3.696529289	-0.045410403
C	-1.030252350	-2.085866473	-2.293613182
O	-1.137355522	-2.139221186	-3.489840028
O	-0.376005079	-3.010963898	-1.577381972
C	-4.874878886	-1.213774281	-1.254381762
O	-5.434229173	-1.177588901	-2.318063509
O	-5.048820898	-0.273171973	-0.313298139
C	-5.525615071	-5.118139451	-0.324180620
O	-6.325474428	-5.614500353	-1.072070884
O	-4.366545190	-5.711410297	-0.000509203
C	-5.936889029	0.780265743	-0.655235714
H	-6.930356015	0.383433357	-0.873642705
H	-5.969961516	1.444373407	0.204885219
H	-5.573007189	1.310513113	-1.537640150
C	0.226735147	-4.060298610	-2.323492853
H	0.536476223	-4.812223033	-1.601833582
H	-0.487520488	-4.481974640	-3.032401017
H	1.087864985	-3.688214035	-2.882012751
C	-4.113388949	-6.955769242	-0.635333692

H	-3.169682610	-7.317367277	-0.234688001
H	-4.918018341	-7.662172390	-0.422263586
H	-4.044841537	-6.820011671	-1.716757410
C	5.220418940	-1.258127138	-3.747713350
C	4.286014138	-2.232117228	-4.070465466
H	3.431038985	-1.969659568	-4.671055908
C	4.424248026	-3.533194212	-3.600503148
C	5.531536495	-3.867067056	-2.824306095
H	5.682896365	-4.868155883	-2.453550643
C	6.455715142	-2.888817628	-2.505103043
H	7.301121497	-3.147184796	-1.883288245
C	6.314563507	-1.582674200	-2.943614566
C	7.291303042	-0.522976065	-2.499228498
C	6.834071515	0.851667777	-2.921564985
C	7.489409789	1.979235221	-2.450077138
H	8.351422134	1.859937030	-1.809611742
C	7.052598394	3.252395368	-2.756785020
H	7.583226448	4.096250120	-2.349951178
C	5.922342567	3.415711706	-3.555962854
C	5.278252904	2.291287259	-4.064453750
H	4.401975130	2.410139869	-4.679926711
C	5.720397640	1.017994948	-3.741941454
C	8.703201017	-0.803714450	-2.938655790
C	9.236231635	-0.918601564	-4.205756352
H	8.615013211	-0.813377553	-5.081974656
C	10.595677805	-1.173126549	-4.311981600
H	11.040631681	-1.269194179	-5.291182435
C	11.397924353	-1.305699577	-3.184314551
H	12.452620665	-1.502167129	-3.304914902
C	10.862789462	-1.188420316	-1.912732121
H	11.469286869	-1.286701324	-1.026129673
C	9.505145921	-0.936621497	-1.809454483
C	8.638177138	-0.770026333	-0.625817705
O	3.419597219	-4.386050553	-3.936767475
O	5.004538652	-0.021507058	-4.250189091
O	5.373188475	4.607320655	-3.889075350
O	7.383575772	-0.556554841	-1.052061327
O	8.924414950	-0.818013316	0.543520492
C	5.833586042	5.770869893	-3.211573913
H	5.919634343	5.572737674	-2.134854093
H	6.813083245	6.082174968	-3.605481420
C	3.518082539	-5.741835316	-3.535402146
H	4.421785271	-6.205607616	-3.959708383
H	3.558410558	-5.819513485	-2.437248956
C	2.347697731	-6.451631077	-4.018990731
C	1.373840139	-7.015535796	-4.423623123
C	4.871220799	6.836020727	-3.419245724
C	4.052356371	7.693564669	-3.580498554
H	0.531709953	-7.525979601	-4.784832015
H	3.352034049	8.460038558	-3.729874433

PMMA + 1

C	0.752400000	0.538600000	-1.178900000
C	0.306600000	1.746100000	-1.994600000
O	0.442400000	1.879000000	-3.179600000
O	-0.286600000	2.680600000	-1.227200000
C	-0.718600000	3.842400000	-1.913600000
H	-1.450700000	3.577300000	-2.680300000
H	-1.165600000	4.491300000	-1.163300000
H	0.128500000	4.340000000	-2.394800000
C	1.554900000	1.030600000	0.058200000

H	0.972300000	1.833700000	0.514100000
H	1.602100000	0.208300000	0.775600000
C	3.005200000	1.548900000	-0.131200000
C	1.541200000	-0.401900000	-2.086200000
H	1.769600000	-1.329800000	-1.566900000
H	0.964000000	-0.617600000	-2.979400000
H	2.468100000	0.047200000	-2.422600000
C	-0.480400000	-0.188500000	-0.574300000
H	-0.083300000	-1.052800000	-0.037100000
H	-0.929200000	0.486700000	0.159000000
C	3.413100000	2.204900000	1.210100000
H	3.376000000	1.425000000	1.972900000
H	2.656800000	2.952600000	1.458300000
C	-1.641400000	-0.704700000	-1.467700000
C	4.796600000	2.905200000	1.317400000
C	4.969800000	3.172000000	2.836700000
H	4.931700000	2.196800000	3.327200000
H	4.098300000	3.747100000	3.159500000
C	6.218300000	3.900700000	3.396800000
C	6.007500000	3.887500000	4.933600000
H	6.053700000	2.845900000	5.258900000
H	4.997900000	4.254500000	5.129200000
C	6.958900000	4.690600000	5.838900000
C	6.568800000	4.387700000	7.299000000
H	6.742200000	3.336600000	7.517900000
H	7.169800000	4.984900000	7.981500000
H	5.518900000	4.613400000	7.470900000
C	3.118100000	2.542100000	-1.282600000
H	2.798200000	2.110200000	-2.224400000
H	2.508900000	3.420300000	-1.078500000
H	4.148300000	2.854500000	-1.416700000
C	5.942400000	2.062500000	0.766900000
H	6.040500000	1.138400000	1.329700000
H	5.792800000	1.825200000	-0.281600000
H	6.874500000	2.616500000	0.826700000
C	6.346900000	5.320300000	2.853500000
H	6.589800000	5.297800000	1.795700000
H	5.418200000	5.865700000	3.003300000
H	7.155600000	5.848400000	3.351400000
C	8.432900000	4.343600000	5.642000000
H	8.583300000	3.273700000	5.763700000
H	8.782400000	4.641700000	4.658800000
H	9.035200000	4.869100000	6.379300000
C	3.887100000	0.327800000	-0.363400000
O	4.479000000	0.077500000	-1.378000000
O	3.906500000	-0.480600000	0.703000000
C	4.751500000	4.229800000	0.563700000
O	5.545400000	4.578400000	-0.272400000
O	3.723400000	4.994000000	0.949100000
C	7.491000000	3.123100000	3.062000000
O	8.441100000	3.583900000	2.484500000
O	7.440600000	1.869700000	3.518600000
C	6.768900000	6.195300000	5.693200000
O	7.652800000	7.010300000	5.717500000
O	5.471600000	6.535000000	5.624200000
C	4.710900000	-1.645900000	0.577400000
H	4.717500000	-2.117700000	1.557000000
H	4.287500000	-2.318200000	-0.171900000
H	5.721300000	-1.373300000	0.268300000
C	3.611300000	6.260000000	0.310500000
H	3.361100000	6.142200000	-0.748600000

H	2.818600000	6.792000000	0.831000000
H	4.553500000	6.805500000	0.388200000
C	8.579900000	1.052200000	3.263900000
H	9.494300000	1.630800000	3.403600000
H	8.532300000	0.230600000	3.974800000
H	8.559400000	0.665200000	2.243100000
C	5.203400000	7.925800000	5.539200000
H	4.121200000	8.027300000	5.510500000
H	5.615300000	8.446400000	6.406100000
H	5.652500000	8.342900000	4.635400000
C	-2.501600000	-1.570700000	-0.513100000
H	-1.868500000	-2.405100000	-0.206400000
H	-2.720500000	-0.975000000	0.376000000
C	-3.848300000	-2.171700000	-1.001200000
C	-4.191500000	-3.261300000	0.046400000
H	-3.334800000	-3.937400000	0.093800000
H	-4.286300000	-2.767900000	1.016400000
C	-5.442400000	-4.138800000	-0.148300000
C	-2.424800000	0.454100000	-2.078300000
H	-2.679700000	1.182400000	-1.310900000
H	-1.831300000	0.933800000	-2.850600000
H	-3.337800000	0.107700000	-2.551100000
C	-3.773100000	-2.766600000	-2.403300000
H	-3.525100000	-2.009700000	-3.140200000
H	-3.022500000	-3.551400000	-2.433100000
H	-4.738600000	-3.179300000	-2.686700000
C	-5.539800000	-5.069900000	1.074000000
H	-5.709700000	-4.485300000	1.975500000
H	-6.367800000	-5.767000000	0.955500000
H	-4.614300000	-5.630700000	1.191500000
C	-6.737200000	-3.337200000	-0.263400000
H	-6.836900000	-2.666100000	0.586300000
H	-6.759900000	-2.760500000	-1.182700000
H	-7.589700000	-4.012300000	-0.280100000
C	-1.101400000	-1.603200000	-2.580800000
O	-1.218700000	-1.385600000	-3.758100000
O	-0.507700000	-2.690300000	-2.081800000
C	-4.908200000	-1.077000000	-0.980100000
O	-5.558600000	-0.714400000	-1.924700000
O	-5.043800000	-0.533300000	0.239600000
C	-5.321600000	-5.061900000	-1.357200000
O	-6.213200000	-5.302200000	-2.126800000
O	-4.119300000	-5.658800000	-1.429200000
C	-6.003300000	0.507400000	0.344700000
H	-6.990500000	0.144400000	0.051700000
H	-6.003000000	0.820400000	1.385900000
H	-5.731400000	1.340200000	-0.307000000
C	0.053200000	-3.601400000	-3.020400000
H	0.216500000	-4.538700000	-2.490800000
H	-0.624800000	-3.736700000	-3.865000000
H	1.007100000	-3.220400000	-3.387200000
C	-3.901100000	-6.458800000	-2.584100000
H	-2.951200000	-6.968800000	-2.428500000
H	-4.716400000	-7.172900000	-2.715300000
H	-3.846300000	-5.823900000	-3.470700000
C	5.117600000	-1.648900000	-3.458200000
C	4.160200000	-2.652400000	-3.496200000
H	3.242600000	-2.479700000	-4.033700000
C	4.347500000	-3.846500000	-2.805500000
C	5.563400000	-4.064500000	-2.155700000
H	5.782200000	-5.001400000	-1.668700000

C	6.507000000	-3.055900000	-2.114400000
H	7.427300000	-3.218500000	-1.570900000
C	6.289900000	-1.830800000	-2.724100000
C	7.255000000	-0.691300000	-2.510000000
C	6.686500000	0.604500000	-3.035400000
C	7.275300000	1.815200000	-2.700100000
H	8.178600000	1.816400000	-2.106700000
C	6.715800000	3.019800000	-3.075100000
H	7.191900000	3.937300000	-2.769800000
C	5.531300000	3.030100000	-3.813500000
C	4.965900000	1.818500000	-4.204600000
H	4.048200000	1.818100000	-4.769800000
C	5.521900000	0.617000000	-3.799900000
C	8.633600000	-0.965200000	-3.044600000
C	9.060000000	-1.193500000	-4.336700000
H	8.361000000	-1.202800000	-5.159000000
C	10.415100000	-1.408200000	-4.539800000
H	10.777700000	-1.590000000	-5.540700000
C	11.317200000	-1.392700000	-3.482000000
H	12.365600000	-1.561900000	-3.677500000
C	10.889100000	-1.162500000	-2.185700000
H	11.574100000	-1.145100000	-1.352400000
C	9.535600000	-0.950200000	-1.985300000
C	8.767800000	-0.690600000	-0.750100000
O	3.297400000	-4.702500000	-2.800400000
O	4.840800000	-0.514000000	-4.142400000
O	4.862200000	4.139300000	-4.185900000
O	7.475400000	-0.558000000	-1.080400000
O	9.158300000	-0.614800000	0.387700000
C	5.026900000	5.342100000	-3.428000000
H	5.418600000	5.121400000	-2.428000000
H	5.727100000	6.009900000	-3.951600000
C	3.339100000	-5.822500000	-1.920700000
H	3.908400000	-6.641100000	-2.389100000
H	3.832600000	-5.543600000	-0.977300000
C	1.992300000	-6.277700000	-1.598900000
C	0.893100000	-6.648900000	-1.272400000
C	3.723100000	5.978200000	-3.294900000
C	2.625600000	6.458500000	-3.161200000
Au	-0.779900000	-7.380300000	-0.574400000
P	-2.514500000	-8.456300000	0.447800000
N	-3.027500000	-10.954600000	1.552600000
N	-4.969500000	-9.639000000	0.939900000
N	-3.668700000	-9.040900000	2.900300000
C	-2.085000000	-10.252700000	0.710200000
H	-2.037200000	-10.749800000	-0.266800000
H	-1.088000000	-10.304400000	1.165400000
C	-4.309000000	-8.763400000	0.000600000
H	-4.841300000	-7.807500000	-0.034300000
H	-4.345200000	-9.217500000	-0.997300000
C	-2.807100000	-8.077900000	2.251800000
H	-1.834200000	-8.065900000	2.759900000
H	-3.250700000	-7.079700000	2.347200000
C	-4.985700000	-9.087000000	2.284300000
H	-5.402600000	-8.076900000	2.254100000
H	-5.627500000	-9.720500000	2.904000000
C	-3.092300000	-10.375900000	2.884500000
H	-2.085100000	-10.336900000	3.308400000
H	-3.716900000	-11.023300000	3.507200000
C	-4.362000000	-10.958900000	0.975800000
H	-4.312900000	-11.360400000	-0.040000000

H	-4.995200000	-11.609400000	1.586600000
Au	0.818700000	7.189200000	-3.103400000
P	-1.296400000	8.005600000	-3.171100000
N	-4.019400000	7.790300000	-2.706200000
N	-3.376600000	8.783000000	-4.825600000
N	-3.125300000	10.047600000	-2.768600000
C	-2.769000000	7.113400000	-2.444600000
H	-2.809000000	6.103100000	-2.870000000
H	-2.630000000	7.023800000	-1.359900000
C	-2.033300000	8.249000000	-4.865300000
H	-1.389000000	8.932700000	-5.432200000
H	-2.042700000	7.282300000	-5.383900000
C	-1.745800000	9.696100000	-2.515100000
H	-1.561400000	9.717700000	-1.433700000
H	-1.091300000	10.439500000	-2.987000000
C	-3.418700000	10.090800000	-4.192800000
H	-2.701700000	10.752200000	-4.686500000
H	-4.426300000	10.498100000	-4.319200000
C	-4.044900000	9.123300000	-2.125500000
H	-3.800700000	9.056100000	-1.061700000
H	-5.058200000	9.521500000	-2.234700000
C	-4.290700000	7.891000000	-4.132200000
H	-4.230500000	6.895500000	-4.579700000
H	-5.306000000	8.278800000	-4.257600000

PMMA + 2

C	0.764501721	0.649877751	-1.221440023
C	0.259284827	1.854057377	-2.006510523
O	0.346800721	2.003949399	-3.193944054
O	-0.326002631	2.768462685	-1.207365915
C	-0.809729361	3.926652890	-1.863595850
H	-1.535338811	3.650323149	-2.632403455
H	-1.278820532	4.537324911	-1.095111114
H	0.013773101	4.470141652	-2.336900711
C	1.581190393	1.151397750	0.003010328
H	1.003483099	1.957858639	0.458687674
H	1.634157891	0.335299056	0.726834641
C	3.031154916	1.663497358	-0.199125989
C	1.548991966	-0.256482154	-2.167211006
H	1.781954762	-1.203237496	-1.685771306
H	0.961020774	-0.439449352	-3.061057274
H	2.473433370	0.202588821	-2.496470856
C	-0.422660208	-0.126140101	-0.588469501
H	0.024815430	-0.987722937	-0.087745058
H	-0.861264954	0.518196631	0.177961673
C	3.445444540	2.322896433	1.138167114
H	3.386924761	1.550781826	1.907441658
H	2.703359004	3.088498967	1.374675748
C	-1.606766818	-0.663040808	-1.437668837
C	4.842572322	2.994199206	1.250826820
C	5.008136117	3.262127965	2.770723532
H	4.951688742	2.288323883	3.262252703
H	4.142519439	3.850453172	3.085336679
C	6.261701093	3.973114501	3.340932258
C	6.038616452	3.959433770	4.875971460
H	6.070314249	2.916764595	5.199427941
H	5.031776473	4.337603204	5.064478850
C	6.992280170	4.749601770	5.789923240
C	6.588534522	4.447409380	7.246433034
H	6.749796366	3.394129725	7.464151617
H	7.190493970	5.036957370	7.934631048

H	5.539671876	4.683147744	7.411286043
C	3.143180825	2.653888583	-1.353328855
H	2.784996441	2.235889239	-2.287581730
H	2.563950479	3.548298688	-1.132883023
H	4.178348512	2.937925103	-1.511892939
C	5.974851972	2.126456571	0.711594276
H	6.050933951	1.202596067	1.278052487
H	5.827225654	1.889906530	-0.337422661
H	6.917333625	2.662159148	0.775945332
C	6.412147585	5.391520219	2.800501689
H	6.660720547	5.366835208	1.743956554
H	5.489792896	5.948772857	2.945678475
H	7.224830855	5.908509264	3.303666989
C	8.463407917	4.386449384	5.601886817
H	8.601557023	3.315204668	5.726398701
H	8.821750548	4.678899241	4.620216285
H	9.067162222	4.906793185	6.341657176
C	3.900586107	0.432346485	-0.424124535
O	4.486414690	0.164022876	-1.437417158
O	3.911246475	-0.364335763	0.651535779
C	4.831603856	4.314481898	0.489070475
O	5.635081106	4.637003415	-0.348170620
O	3.822024420	5.107112153	0.866758574
C	7.525672283	3.178789582	3.012783342
O	8.483259116	3.625901118	2.436989319
O	7.457237200	1.927108696	3.471896592
C	6.819754709	6.256558695	5.646884507
O	7.712195248	7.061834838	5.679585860
O	5.526561885	6.610663484	5.569329138
C	4.694737995	-1.544570729	0.540114632
H	4.690022664	-2.006237031	1.524639530
H	4.260597740	-2.216480050	-0.203240881
H	5.711004581	-1.294285516	0.231664650
C	3.747790015	6.372241045	0.221518632
H	3.510422277	6.254591846	-0.840670099
H	2.960451592	6.924401831	0.729086144
H	4.701081404	6.896366077	0.310660342
C	8.587050004	1.094058397	3.224748216
H	9.507949273	1.663979845	3.356739216
H	8.531497005	0.280786101	3.944620316
H	8.560540164	0.696073834	2.208453495
C	5.274309831	8.004688284	5.489999151
H	4.193424974	8.118864315	5.461820757
H	5.692174753	8.517076239	6.358940804
H	5.728248062	8.420499570	4.587927512
C	-2.348680153	-1.620534828	-0.472217001
H	-1.652486333	-2.432834339	-0.255427982
H	-2.531272226	-1.078652812	0.458519630
C	-3.693779269	-2.274426707	-0.885886334
C	-3.909697608	-3.390218206	0.166667170
H	-3.034714617	-4.043530465	0.117865163
H	-3.920275240	-2.913306877	1.149506484
C	-5.151677396	-4.300069931	0.092547146
C	-2.487552550	0.479270156	-1.935812020
H	-2.754061788	1.139345246	-1.112764602
H	-1.958460601	1.045266261	-2.696558605
H	-3.395518814	0.102299618	-2.395692253
C	-3.676667117	-2.850999999	-2.297057989
H	-3.487355966	-2.078017667	-3.035033877
H	-2.907615473	-3.615074935	-2.374026100
H	-4.644998272	-3.284964484	-2.534233249

C	-5.072122526	-5.245273848	1.302648485
H	-5.099786256	-4.667121723	2.224350572
H	-5.906930848	-5.943338410	1.312348630
H	-4.135698328	-5.802631944	1.264656174
C	-6.472673309	-3.535651884	0.144728078
H	-6.487303309	-2.875370003	1.008156889
H	-6.623753854	-2.954179627	-0.759390207
H	-7.299587514	-4.237206137	0.225624638
C	-1.104169087	-1.469511727	-2.634284154
O	-1.290824057	-1.177176408	-3.786422596
O	-0.450266618	-2.567472436	-2.248033592
C	-4.800810387	-1.233226919	-0.781888013
O	-5.530493148	-0.896333916	-1.676569868
O	-4.875617427	-0.704153540	0.449466933
C	-5.144435988	-5.182338691	-1.149379836
O	-6.085183998	-5.351122267	-1.876967088
O	-3.970885414	-5.825078741	-1.330504517
C	-5.878908729	0.282569297	0.634440125
H	-6.867904711	-0.143971498	0.454777895
H	-5.789532633	0.621158027	1.663655020
H	-5.726410485	1.112199734	-0.058603939
C	0.064406338	-3.389557108	-3.287740069
H	0.550796885	-4.231377389	-2.800568447
H	-0.746035481	-3.728918994	-3.935527412
H	0.780396139	-2.829896681	-3.890175694
C	-3.873515833	-6.556483271	-2.546505240
H	-2.957209046	-7.140846421	-2.484482496
H	-4.746914877	-7.198242000	-2.677055791
H	-3.820403617	-5.866035631	-3.390561809
C	5.123239292	-1.627420366	-3.456971903
C	4.130358646	-2.596497223	-3.468692057
H	3.218972156	-2.409347415	-4.011037673
C	4.270955153	-3.771042190	-2.735901419
C	5.476013304	-4.015347992	-2.075980416
H	5.656749860	-4.944011161	-1.558402109
C	6.456485182	-3.041538118	-2.062995077
H	7.368340132	-3.220090233	-1.510656513
C	6.283938549	-1.827986939	-2.709028509
C	7.281457865	-0.713147124	-2.514099174
C	6.747363257	0.589056889	-3.059339657
C	7.357657237	1.790284876	-2.728654870
H	8.253202811	1.778703878	-2.123854783
C	6.828175341	3.002225141	-3.122658198
H	7.319866176	3.912688142	-2.820931029
C	5.653779397	3.029685560	-3.876537331
C	5.067890812	1.826375179	-4.262926740
H	4.158111604	1.839680017	-4.840422009
C	5.593044839	0.617830260	-3.838710070
C	8.651958882	-1.031951506	-3.044620763
C	9.071348824	-1.292011442	-4.332989986
H	8.371837043	-1.296691945	-5.154825235
C	10.420360963	-1.544711575	-4.532756168
H	10.777568600	-1.751946840	-5.530660135
C	11.323245999	-1.534928072	-3.475606228
H	12.366804929	-1.733878640	-3.668545013
C	10.902092776	-1.272723499	-2.183016404
H	11.587753344	-1.258968874	-1.350314780
C	9.554653837	-1.022727425	-1.985811213
C	8.794933743	-0.723570928	-0.754686082
O	3.187369353	-4.585087324	-2.698888439
O	4.891103900	-0.503037747	-4.174693404

O	5.014116907	4.149181172	-4.269761226
O	7.505529018	-0.563450908	-1.087206760
O	9.186981766	-0.639078346	0.381660495
C	5.186103242	5.353121688	-3.515925558
H	5.575380012	5.134809093	-2.514588617
H	5.889933653	6.014401684	-4.042447327
C	3.162948471	-5.630631949	-1.731984292
H	3.691794400	-6.513414760	-2.124824995
H	3.662147607	-5.302480316	-0.807707159
C	1.790774836	-5.990233696	-1.395790466
C	0.668836133	-6.273617930	-1.059365773
C	3.886983176	5.998377471	-3.384361025
C	2.793039647	6.485828819	-3.248588992
Au	-1.027872940	-6.973689509	-0.386693364
Au	1.004674360	7.257255296	-3.183563731
P	-1.077623976	8.153351077	-3.205267100
O	-4.222806261	11.840533525	-2.654557419
O	-2.356135275	10.915388602	-5.852160426
N	-3.751645696	8.116071926	-2.713472199
N	-3.125398324	9.062534480	-4.851873516
N	-2.667492575	10.262290315	-2.334575521
C	-1.316974079	9.771149365	-2.312220821
H	-0.984253656	9.601673034	-1.273382714
H	-0.633409995	10.486690063	-2.775398597
C	-1.797812612	8.494352837	-4.873415979
H	-1.141963504	9.186702991	-5.415450136
H	-1.806721258	7.530927965	-5.407699557
C	-2.579267002	7.291066532	-2.538787841
H	-2.710892240	6.340591477	-3.068277900
H	-2.444016296	7.075222026	-1.473214573
C	-3.694252006	9.315372708	-1.920354179
H	-3.511958051	9.017614186	-0.879118940
H	-4.651117164	9.839984290	-1.985819102
C	-4.111546401	8.313478877	-4.098657489
H	-5.065212896	8.838884948	-4.122038757
H	-4.231953150	7.318362138	-4.549413524
C	-3.046121136	11.535468183	-2.660652554
C	-1.962526638	12.514352324	-3.024705092
H	-1.186448102	12.560525831	-2.264546311
H	-2.423519906	13.493337983	-3.125329863
H	-1.526288172	12.243175359	-3.985161917
C	-3.310642730	10.282099555	-5.447441038
C	-4.726949209	10.768052287	-5.599515643
H	-5.108246437	11.120855419	-4.642624371
H	-5.377111823	9.991614587	-5.993916719
H	-4.711939008	11.610421794	-6.286225754
P	-2.694197868	-8.200536220	0.604944197
O	-4.707662846	-10.100990323	4.626401252
O	-6.532146256	-8.276297164	1.751330309
N	-2.905451728	-10.810891826	1.415789309
N	-5.007587004	-9.710589278	0.951383117
N	-3.303234236	-9.166205149	3.154859331
C	-2.708836082	-8.055116818	2.469203329
H	-1.642517090	-7.965670136	2.744072748
H	-3.207417498	-7.123955870	2.738740199
C	-4.438381627	-8.663869324	0.140625967
H	-5.096518004	-7.793824018	0.194411900
H	-4.378890311	-9.001422484	-0.905902131
C	-2.101025818	-9.960187319	0.569884714
H	-2.136412553	-10.330825729	-0.461497800
H	-1.059471676	-9.998498602	0.907587966

C	-2.766720120	-10.474879076	2.807224402
H	-1.696967084	-10.502993505	3.053150982
H	-3.299225281	-11.210147436	3.415856012
C	-4.266091080	-10.956801405	0.957525315
H	-4.762076055	-11.676864163	1.607039472
H	-4.225912351	-11.356048957	-0.066350952
C	-4.274517160	-9.087648981	4.112952262
C	-4.778661504	-7.718961496	4.487690625
H	-3.967194706	-7.056307312	4.779932429
H	-5.459901323	-7.838805217	5.326135066
H	-5.330893465	-7.285436689	3.655405898
C	-6.129740627	-9.421716622	1.678956158
C	-6.840881696	-10.571692928	2.338891524
H	-6.288330657	-10.903645550	3.216406743
H	-6.977469253	-11.403553086	1.652692670
H	-7.812035823	-10.211822851	2.668655276

Polystyrene + L

C	0.134064502	2.497369703	0.165102631
C	1.379025722	2.376459767	-0.441717772
C	2.322363386	3.380866592	-0.330460218
C	2.034586706	4.535234891	0.376112332
C	0.799162307	4.666572901	0.985946616
C	-0.136744776	3.652603005	0.888055478
H	1.619135001	1.487160444	-1.005900928
H	3.287592034	3.264645467	-0.801716419
H	2.765565691	5.326325942	0.448209125
H	0.561444934	5.564038724	1.539075384
H	-1.096629704	3.756355224	1.375036919
C	-0.915954001	1.420081188	0.027318564
H	-1.782781892	1.703629386	0.634744262
C	-0.392858547	0.065936840	0.527281430
H	-1.207359203	-0.660774314	0.518529990
H	0.377448513	-0.292785971	-0.159131001
C	-1.361001523	1.310625740	-1.439403714
H	-2.138481164	0.549730421	-1.526856705
H	-0.508208921	0.986260436	-2.039977150
C	0.212251205	0.175172017	1.932136658
H	0.832445153	1.077603546	1.949399098
C	1.139645009	-1.006212549	2.251414397
H	0.555354869	-1.915938913	2.399838017
H	1.816196713	-1.176261402	1.411931468
C	1.943506759	-0.698422641	3.520995887
H	1.222967954	-0.397508328	4.288913327
C	2.683480867	-1.932108055	4.051002594
H	1.986337843	-2.772247369	4.072416395
H	3.506585696	-2.198640294	3.385309877
C	3.231295534	-1.692399847	5.465137504
H	3.640341668	-2.630743345	5.850152341
H	4.042789856	-0.963387930	5.416916055
C	-1.861562569	2.658231723	-1.977371751
H	-1.163694496	3.422821483	-1.621943430
C	-1.833861599	2.710904318	-3.511324488
H	-2.642195057	2.108029722	-3.929230284
H	-0.890037354	2.290088743	-3.863399291
C	-1.963185049	4.153474115	-4.021854645
H	-2.951148824	4.528366132	-3.731229553
C	-1.859403560	4.199013382	-5.547867170
H	-2.637992496	3.587800905	-5.999325950
H	-1.971427637	5.220839686	-5.903799716
H	-0.892937492	3.826009915	-5.878913906

C	-0.841386142	0.308763570	3.003418884
C	-1.727964209	-0.727141164	3.272239164
C	-0.911750016	1.456878466	3.783329796
C	-2.637906775	-0.632146135	4.309525309
H	-1.704866950	-1.625290535	2.671073979
C	-1.823207846	1.556363810	4.819897182
H	-0.238621952	2.277034837	3.573225758
C	-2.681104478	0.506532120	5.094566546
H	-3.311606736	-1.453694071	4.508463255
H	-1.863639209	2.448286394	5.426435315
H	-3.375948432	0.581282031	5.917919437
C	2.879778927	0.457857117	3.274986325
C	3.874010984	0.391606259	2.306109359
C	2.741656179	1.635111753	3.998503700
C	4.694502465	1.476871141	2.056785406
H	4.009132071	-0.514947738	1.733185710
C	3.569634802	2.717410400	3.762623294
H	1.974617772	1.701920206	4.756314577
C	4.543271101	2.646018324	2.782062888
H	5.455728813	1.409761647	1.292455359
H	3.446581238	3.617458968	4.344639491
H	5.180770783	3.495610942	2.584604638
C	-3.232108163	2.992531279	-1.448423104
C	-3.425559611	4.127592803	-0.671296595
C	-4.330352479	2.189037415	-1.731452878
C	-4.681188016	4.452560261	-0.188895651
H	-2.580647607	4.763625176	-0.448047331
C	-5.586962383	2.510719162	-1.251465453
H	-4.206859927	1.301282059	-2.335680089
C	-5.767042539	3.644661405	-0.478064947
H	-4.812829810	5.339928726	0.413775791
H	-6.429922243	1.875048453	-1.482550923
H	-6.748606410	3.896942960	-0.104069689
C	-0.917575255	5.041510538	-3.390525934
C	-1.294461921	6.167679162	-2.670378880
C	0.437153863	4.745078442	-3.490098906
C	-0.348609925	6.964374196	-2.048953419
H	-2.343537394	6.416887538	-2.588255341
C	1.383635811	5.538634162	-2.869593245
H	0.759543420	3.878802965	-4.048901163
C	0.995215346	6.648953142	-2.140366701
H	-0.663514130	7.831718917	-1.486445315
H	2.430472431	5.283952516	-2.946526675
H	1.736938179	7.259779922	-1.647394379
C	2.157237494	-1.183034753	6.387167089
C	2.258590702	0.073290942	6.970451384
C	1.011236404	-1.933757436	6.624575201
C	1.227728047	0.582746940	7.740866920
H	3.150576619	0.662465405	6.807881825
C	-0.021478614	-1.424913909	7.390089508
H	0.922101631	-2.921905806	6.196169721
C	0.077709623	-0.159349196	7.942175459
H	1.317557005	1.568596301	8.174155668
H	-0.911712018	-2.015260161	7.551195229
H	-0.738947351	0.245833547	8.519344120
C	1.040943965	5.236957128	6.467592263
C	0.246983179	4.137630106	6.748998042
H	0.252511491	3.289176113	6.085397841
C	-0.572760443	4.129437948	7.874523481
C	-0.571341979	5.230506517	8.728334028
H	-1.190001681	5.260599483	9.610584139

C	0.243762780	6.311603267	8.449381444
H	0.243074464	7.161395985	9.115320497
C	1.051447617	6.336431694	7.324718952
C	1.978098991	7.497610619	7.050761717
C	2.401015973	7.506875447	5.600830900
C	2.936223551	8.641815016	5.014565803
H	3.009398327	9.548912217	5.595521429
C	3.366998655	8.641028819	3.700785215
H	3.774084769	9.549329513	3.286698624
C	3.274412578	7.473239191	2.947117885
C	2.745343156	6.324494511	3.527828638
H	2.662763068	5.420775688	2.947543435
C	2.304992518	6.342216190	4.840974140
C	3.183403429	7.438191843	7.959023883
C	4.165349114	6.477448329	8.080761048
H	4.157706726	5.587981063	7.469155077
C	5.163362336	6.696011015	9.019260844
H	5.946184575	5.961816308	9.138382906
C	5.175659941	7.839721522	9.809066596
H	5.967844928	7.976475946	10.529673269
C	4.187878625	8.802507918	9.684372940
H	4.180883554	9.695660646	10.288953386
C	3.192989601	8.582174372	8.747129917
C	2.015578265	9.395639917	8.373122217
O	-1.330029729	3.018351763	8.033342581
O	1.780749617	5.179680104	5.324358880
O	3.657957711	7.343628837	1.653841252
O	1.347700727	8.738993727	7.398786987
O	1.656731790	10.453423127	8.810607913
C	4.104389378	8.493492812	0.953325135
H	3.353801401	9.296735168	1.011225605
H	5.054913738	8.858547302	1.372655322
C	-2.244198552	2.963163651	9.115661292
H	-1.708525225	2.987339686	10.077605994
H	-2.944900544	3.811290407	9.074092880
C	-2.994563766	1.724211902	9.018302161
C	-3.597349536	0.696316399	8.912458322
C	4.300946494	8.128641723	-0.438002721
C	4.438224908	7.796381053	-1.578938722
H	4.563825902	7.529072407	-2.585703274
H	-4.134595633	-0.201803366	8.839731479

Polystyrene + 1

C	0.083600000	2.409100000	0.163900000
C	1.309400000	2.344300000	-0.488900000
C	2.240000000	3.357900000	-0.353000000
C	1.962100000	4.463400000	0.431900000
C	0.752400000	4.530600000	1.101000000
C	-0.172600000	3.509400000	0.972800000
H	1.542600000	1.494700000	-1.114800000
H	3.188300000	3.287900000	-0.866400000
H	2.680900000	5.265400000	0.514800000
H	0.523300000	5.384200000	1.722500000
H	-1.113200000	3.565700000	1.503500000
C	-0.959800000	1.329400000	-0.010100000
H	-1.835700000	1.596100000	0.592300000
C	-0.430500000	-0.028100000	0.472500000
H	-1.211400000	-0.781600000	0.357100000
H	0.408100000	-0.322800000	-0.162400000
C	-1.390300000	1.229900000	-1.481700000
H	-2.196400000	0.499400000	-1.573000000

H	-0.545400000	0.867000000	-2.071500000
C	0.054000000	0.039600000	1.924800000
H	0.611600000	0.976000000	2.031200000
C	1.029800000	-1.100400000	2.262000000
H	0.478500000	-2.027700000	2.434000000
H	1.706600000	-1.263600000	1.421400000
C	1.838600000	-0.737500000	3.514200000
H	1.120700000	-0.454200000	4.291600000
C	2.638900000	-1.932200000	4.045500000
H	1.961000000	-2.782100000	4.152900000
H	3.416500000	-2.215700000	3.333400000
C	3.286800000	-1.620700000	5.402600000
H	3.710600000	-2.542300000	5.812200000
H	4.102100000	-0.909800000	5.255300000
C	-1.834000000	2.588300000	-2.040800000
H	-1.089400000	3.323300000	-1.720200000
C	-1.842100000	2.589300000	-3.575600000
H	-2.657600000	1.969500000	-3.952600000
H	-0.906200000	2.154400000	-3.932800000
C	-1.989300000	4.009000000	-4.143500000
H	-2.988100000	4.376400000	-3.883000000
C	-1.867200000	3.991000000	-5.669000000
H	-2.636000000	3.353600000	-6.100000000
H	-1.986700000	4.994600000	-6.073200000
H	-0.896100000	3.606000000	-5.974800000
C	-1.072900000	0.042700000	2.927300000
C	-2.018300000	-0.975300000	2.951800000
C	-1.144700000	1.031700000	3.902100000
C	-3.008300000	-0.996900000	3.918100000
H	-1.986100000	-1.762400000	2.210800000
C	-2.110200000	0.994100000	4.892900000
H	-0.427800000	1.839800000	3.879500000
C	-3.050600000	-0.021600000	4.899200000
H	-3.754400000	-1.782000000	3.898300000
H	-2.131300000	1.747000000	5.666300000
H	-3.810100000	-0.046900000	5.668200000
C	2.718300000	0.455100000	3.229100000
C	3.704900000	0.412800000	2.251500000
C	2.524200000	1.645800000	3.917300000
C	4.461400000	1.533700000	1.959500000
H	3.885200000	-0.502800000	1.705600000
C	3.283000000	2.766400000	3.634000000
H	1.770400000	1.693100000	4.689700000
C	4.249300000	2.717300000	2.644900000
H	5.219200000	1.483500000	1.190400000
H	3.109500000	3.677300000	4.184800000
H	4.833300000	3.594900000	2.410200000
C	-3.169800000	3.005800000	-1.483200000
C	-3.271500000	4.145800000	-0.695900000
C	-4.321900000	2.272300000	-1.740000000
C	-4.491000000	4.542500000	-0.176200000
H	-2.383100000	4.726500000	-0.491200000
C	-5.543100000	2.667500000	-1.224400000
H	-4.269400000	1.381300000	-2.349800000
C	-5.631700000	3.804300000	-0.439500000
H	-4.550500000	5.430100000	0.436900000
H	-6.429500000	2.086200000	-1.435200000
H	-6.585000000	4.112200000	-0.035400000
C	-0.976800000	4.940700000	-3.522100000
C	-1.397900000	6.095400000	-2.874800000
C	0.382800000	4.644700000	-3.525300000

C	-0.496200000	6.910100000	-2.211500000
H	-2.449900000	6.344400000	-2.869600000
C	1.285300000	5.453800000	-2.858000000
H	0.736700000	3.753600000	-4.025300000
C	0.847800000	6.582300000	-2.184200000
H	-0.847000000	7.793300000	-1.698200000
H	2.334900000	5.194600000	-2.836100000
H	1.552100000	7.192500000	-1.639500000
C	2.290200000	-1.037500000	6.366700000
C	2.458800000	0.245700000	6.869900000
C	1.145800000	-1.744700000	6.720000000
C	1.492000000	0.826900000	7.672900000
H	3.350000000	0.801500000	6.613500000
C	0.180800000	-1.164100000	7.521000000
H	1.006700000	-2.751700000	6.350000000
C	0.341500000	0.128000000	7.990600000
H	1.630200000	1.835200000	8.036500000
H	-0.718200000	-1.710800000	7.778200000
H	-0.436200000	0.579600000	8.586000000
C	0.996900000	5.413300000	6.414700000
C	0.141100000	4.350800000	6.651200000
H	0.055500000	3.560100000	5.924300000
C	-0.624400000	4.299600000	7.814100000
C	-0.513600000	5.332900000	8.744800000
H	-1.099800000	5.334200000	9.649700000
C	0.357300000	6.377900000	8.505900000
H	0.439700000	7.174800000	9.230600000
C	1.120700000	6.439100000	7.349800000
C	2.087400000	7.577000000	7.124300000
C	2.601200000	7.582800000	5.704600000
C	3.328900000	8.658400000	5.217900000
H	3.512000000	9.503000000	5.866300000
C	3.814000000	8.680700000	3.924900000
H	4.378800000	9.537000000	3.592700000
C	3.571200000	7.599300000	3.077000000
C	2.861300000	6.504900000	3.564900000
H	2.673300000	5.663300000	2.919500000
C	2.377500000	6.496200000	4.862200000
C	3.224700000	7.527000000	8.113700000
C	4.190200000	6.562900000	8.317000000
H	4.215300000	5.663700000	7.720200000
C	5.126100000	6.791600000	9.314900000
H	5.894500000	6.054900000	9.497900000
C	5.095300000	7.949100000	10.084000000
H	5.840200000	8.094200000	10.852200000
C	4.124600000	8.915200000	9.878100000
H	4.084500000	9.819500000	10.464800000
C	3.191700000	8.684200000	8.881700000
C	2.046800000	9.499300000	8.416400000
O	-1.426800000	3.225800000	7.926600000
O	1.687500000	5.383000000	5.241000000
O	3.961000000	7.504900000	1.791700000
O	1.441000000	8.833900000	7.413700000
O	1.671500000	10.567400000	8.816700000
C	4.537700000	8.638500000	1.144400000
H	3.977500000	9.549500000	1.403200000
H	5.585100000	8.760000000	1.462500000
C	-2.206100000	3.034900000	9.107700000
H	-1.571000000	3.118600000	10.002900000
H	-3.004000000	3.791300000	9.162100000
C	-2.761000000	1.692000000	9.012700000

C	-3.153600000	0.561200000	8.875100000
C	4.452700000	8.419400000	-0.292800000
C	4.343500000	8.221500000	-1.476000000
Au	-3.504200000	-1.284100000	8.349400000
P	-3.690400000	-3.364800000	7.473700000
N	-3.841100000	-6.130900000	7.502300000
N	-2.539300000	-5.151100000	5.701400000
N	-4.964400000	-5.021200000	5.656900000
C	-3.796400000	-4.991100000	8.391900000
H	-2.923300000	-5.082200000	9.050200000
H	-4.697300000	-4.988200000	9.018200000
C	-2.306800000	-3.871800000	6.335300000
H	-2.182000000	-3.096300000	5.568800000
H	-1.378400000	-3.926700000	6.917100000
C	-5.081000000	-3.723600000	6.285300000
H	-6.033400000	-3.671300000	6.828000000
H	-5.088000000	-2.945800000	5.512500000
C	-3.739300000	-5.137000000	4.881500000
H	-3.678900000	-4.304100000	4.176300000
H	-3.780500000	-6.076900000	4.322200000
C	-5.008000000	-6.095900000	6.635200000
H	-5.907300000	-5.987300000	7.247700000
H	-5.057000000	-7.046100000	6.094600000
C	-2.646000000	-6.222200000	6.678500000
H	-1.763400000	-6.208400000	7.324100000
H	-2.678800000	-7.173700000	6.138800000
Au	3.900100000	7.863500000	-3.342200000
P	3.117600000	7.423800000	-5.420100000
N	2.346000000	5.651800000	-7.399200000
N	3.001000000	7.865400000	-8.153800000
N	0.871600000	7.546400000	-7.029700000
C	3.016900000	5.697200000	-6.119000000
H	4.033400000	5.298900000	-6.230700000
H	2.472700000	5.067300000	-5.406900000
C	3.762900000	8.227900000	-6.978600000
H	3.731000000	9.317100000	-6.850800000
H	4.809600000	7.932300000	-7.123800000
C	1.329700000	7.864000000	-5.695200000
H	0.719100000	7.322900000	-4.962300000
H	1.204300000	8.939500000	-5.516800000
C	1.610800000	8.279000000	-8.044200000
H	1.571700000	9.347500000	-7.815400000
H	1.128200000	8.102900000	-9.010500000
C	0.973000000	6.122100000	-7.301100000
H	0.464000000	5.575000000	-6.504200000
H	0.475900000	5.920300000	-8.255100000
C	3.046000000	6.433800000	-8.405000000
H	4.089700000	6.110200000	-8.449200000
H	2.572300000	6.244600000	-9.373200000

Polystyrene + 2

C	0.014347474	2.550986165	0.017592192
C	1.203193625	2.596388825	-0.702213660
C	2.066416126	3.669787692	-0.579740608
C	1.757632091	4.724999425	0.261108534
C	0.580771249	4.686597804	0.989162848
C	-0.278711036	3.607903726	0.870476820
H	1.459848510	1.785642182	-1.369201783
H	2.989260423	3.686367501	-1.141704212
H	2.432295514	5.563821945	0.346691378
H	0.329612375	5.500476129	1.654201742

H	-1.194181555	3.583026788	1.445668873
C	-0.942880939	1.390273422	-0.116820072
H	-1.812852773	1.586019181	0.519981674
C	-0.279573356	0.086041843	0.346652979
H	-0.990140746	-0.735574827	0.239200154
H	0.570952645	-0.126362296	-0.305260581
C	-1.423711954	1.229285771	-1.567391179
H	-2.197125964	0.459526188	-1.604037282
H	-0.586246527	0.885261130	-2.179066952
C	0.224575045	0.168594062	1.790639641
H	0.799959927	1.095620621	1.888772839
C	1.173291150	-0.997998498	2.110525786
H	0.602516587	-1.927344927	2.171045723
H	1.903094872	-1.105111909	1.306158088
C	1.891510489	-0.747096035	3.442441653
H	1.127557639	-0.432450999	4.160663599
C	2.536000685	-2.023181839	3.998708787
H	1.810651273	-2.837033673	3.929258297
H	3.406900425	-2.303304411	3.403520501
C	2.959519538	-1.846864071	5.464405894
H	3.269472880	-2.816822843	5.863817216
H	3.816320838	-1.172132864	5.511308205
C	-1.951496884	2.543155123	-2.157336773
H	-1.211029546	3.315554527	-1.927798492
C	-2.065405057	2.458455569	-3.686138402
H	-2.891091159	1.804212847	-3.971493647
H	-1.146367015	2.017877442	-4.079552379
C	-2.274307246	3.838865262	-4.327423686
H	-3.265260884	4.204092713	-4.037280810
C	-2.222473746	3.740475939	-5.853718321
H	-3.089979085	3.202120001	-6.228860882
H	-2.207437356	4.735226115	-6.292487519
H	-1.329048146	3.208097423	-6.172429321
C	-0.898502149	0.189441143	2.798410739
C	-1.829542139	-0.840464528	2.853715744
C	-0.984569111	1.205271906	3.743913445
C	-2.811255333	-0.858410098	3.828033436
H	-1.791189516	-1.643784971	2.131111421
C	-1.949997193	1.178433839	4.735526306
H	-0.277886189	2.022352772	3.697154199
C	-2.866087098	0.142246055	4.781913028
H	-3.536180817	-1.661259560	3.837873435
H	-1.993685578	1.954657290	5.484116612
H	-3.614367390	0.122131959	5.561562644
C	2.889783215	0.374022856	3.296984136
C	3.962408857	0.276671921	2.418649996
C	2.736441395	1.547588874	4.023781000
C	4.846181589	1.328843567	2.259383437
H	4.110441270	-0.628571782	1.846154248
C	3.623911187	2.598018607	3.874973963
H	1.912417121	1.638423100	4.716480082
C	4.677430439	2.496238461	2.983862477
H	5.669265522	1.238144070	1.564728528
H	3.484881316	3.499671829	4.450735733
H	5.361298836	3.321959958	2.853867256
C	-3.257110646	2.950226881	-1.526512999
C	-3.338785002	4.126679977	-0.792153346
C	-4.400528772	2.172136052	-1.661703870
C	-4.531040187	4.517186346	-0.208714401
H	-2.456220320	4.740355302	-0.679018632
C	-5.594373850	2.560211257	-1.080763879

H	-4.362969983	1.251686014	-2.227327147
C	-5.663938358	3.735070529	-0.352022031
H	-4.575789096	5.435080252	0.359621949
H	-6.474687681	1.943970639	-1.197343475
H	-6.596197009	4.038434081	0.101679858
C	-1.253776829	4.821220048	-3.810832079
C	-1.670127520	5.979128463	-3.167459782
C	0.110839614	4.571158738	-3.905307251
C	-0.755435542	6.840782114	-2.589074089
H	-2.726777147	6.194562186	-3.098977899
C	1.028090653	5.429223573	-3.326577350
H	0.459180849	3.679010482	-4.406393560
C	0.597079761	6.557584375	-2.648782217
H	-1.099219744	7.725631758	-2.073266812
H	2.086973612	5.206013860	-3.370239257
H	1.314401946	7.203861501	-2.163615296
C	1.837465928	-1.279828955	6.290026424
C	1.968667680	-0.041225992	6.904112255
C	0.619762819	-1.945107409	6.390617564
C	0.902484135	0.538882182	7.569398786
H	2.913243480	0.480912960	6.842959855
C	-0.445418853	-1.362134840	7.050235402
H	0.505447304	-2.917699357	5.930409841
C	-0.315181397	-0.113732246	7.633397719
H	1.016066958	1.512560014	8.023201925
H	-1.402147404	-1.871157746	7.102022800
H	-1.161196869	0.344605236	8.121031656
C	0.889268351	5.184501478	6.354325780
C	-0.042977952	4.182448016	6.562145315
H	-0.138870239	3.388534011	5.840748661
C	-0.868288817	4.199175977	7.684372535
C	-0.739505725	5.239684648	8.604110146
H	-1.372630598	5.297218081	9.474904249
C	0.213978019	6.218472343	8.400575208
H	0.313538361	7.018307000	9.119650631
C	1.038449498	6.210266446	7.285857709
C	2.105082564	7.262695633	7.101340697
C	2.647384063	7.242537261	5.691966342
C	3.449049295	8.272983559	5.225564621
H	3.672731625	9.101606443	5.881497865
C	3.957456282	8.269761558	3.941589402
H	4.583548949	9.089073061	3.626534901
C	3.659185472	7.212310342	3.081269592
C	2.880219147	6.157266939	3.551172021
H	2.652265982	5.330683429	2.898461125
C	2.378209965	6.172075067	4.841854088
C	3.214813532	7.091855637	8.108879949
C	4.083381854	6.039603371	8.311557573
H	4.034948660	5.150129278	7.701709218
C	5.020140966	6.166653684	9.326562022
H	5.714129661	5.359624126	9.509618489
C	5.084283057	7.311366176	10.112488716
H	5.826898036	7.376091780	10.893650107
C	4.210815272	8.366203885	9.906883570
H	4.245625011	9.262184708	10.506494247
C	3.276770757	8.236597301	8.893433466
C	2.220590879	9.160858775	8.422769273
O	-1.741713070	3.177807900	7.770256143
O	1.623503141	5.097593157	5.210293212
O	4.056865585	7.110641100	1.798647918
O	1.571457537	8.567241773	7.401658329

O	1.939746063	10.254143667	8.831948642
C	4.686923343	8.223166321	1.165678998
H	4.206316812	9.162113815	1.477351088
H	5.752241257	8.257050828	1.442173114
C	-2.601561016	3.063155340	8.903592325
H	-2.022025552	3.153615867	9.834823366
H	-3.367835340	3.852759275	8.879173942
C	-3.206027904	1.740398540	8.830256693
C	-3.648378752	0.624472205	8.723464306
C	4.527408871	8.070922246	-0.273541637
C	4.346982376	7.942987986	-1.457409565
Au	-3.987609461	-1.262834803	8.364593899
Au	3.793525146	7.738602889	-3.316221826
P	2.987084331	7.590028359	-5.427582877
O	1.438276149	5.657758330	-9.586641669
O	-0.844996792	6.836651897	-6.722121513
N	3.113766803	8.559162687	-7.969959348
N	0.869198966	8.229102515	-7.114065012
N	2.676175077	6.175072126	-7.793361659
C	3.090186939	6.019040693	-6.426244119
H	4.143927781	5.694284553	-6.381661982
H	2.477502354	5.276423646	-5.912201852
C	1.252995973	8.158725240	-5.723033896
H	0.555992270	7.486388949	-5.207951611
H	1.179554566	9.155820410	-5.258188016
C	3.759796617	8.707777860	-6.686223494
H	3.668812202	9.745637833	-6.344947028
H	4.825759055	8.474403281	-6.786400060
C	3.324090853	7.250195851	-8.531255248
H	4.406212957	7.068696488	-8.559606559
H	2.922974490	7.223050405	-9.547822778
C	1.742680925	9.009911391	-7.970628873
H	1.380890562	8.965021352	-8.996845184
H	1.743723876	10.055687458	-7.629981531
C	1.730421030	5.422091658	-8.429977540
C	1.079774222	4.316362479	-7.644401899
H	1.816576231	3.638186829	-7.219815738
H	0.435242473	3.764709929	-8.323956345
H	0.463445936	4.739941905	-6.852442383
C	-0.267706033	7.571454037	-7.498849326
C	-0.768961071	7.815263375	-8.898128706
H	-0.122852266	7.318401261	-9.619866655
H	-0.828978355	8.876835074	-9.123839034
H	-1.759966412	7.374985295	-8.972161011
P	-4.059707303	-3.446931592	7.760697973
O	-2.445822915	-8.034959880	7.912500779
O	-5.756235896	-6.753451592	9.394561282
N	-3.673842058	-5.240907280	5.757148478
N	-5.370636679	-5.870864199	7.370154680
N	-2.590800704	-5.801121980	7.860127539
C	-2.696437852	-4.501696978	8.465411932
H	-1.762629655	-3.931892099	8.322548390
H	-2.883864020	-4.584450273	9.538614294
C	-5.547738677	-4.543547330	7.911934391
H	-5.822846190	-4.645182040	8.968722386
H	-6.366713908	-4.027125309	7.385519232
C	-3.758899276	-3.815414666	5.973293753
H	-4.584635975	-3.401807275	5.382298640
H	-2.829393871	-3.337632145	5.644430120
C	-2.524773687	-5.815803635	6.405166046
H	-1.641691080	-5.251516949	6.078525239

H	-2.422857208	-6.861651547	6.103439473
C	-4.919712246	-5.930889092	5.992557256
H	-4.783607825	-6.970999352	5.699572047
H	-5.677079265	-5.465995572	5.344916033
C	-2.512372118	-6.989340799	8.530040731
C	-2.519872447	-6.944486029	10.034737579
H	-1.762986557	-6.266614683	10.421887890
H	-2.318343233	-7.949807568	10.395107941
H	-3.504252143	-6.645542022	10.392329035
C	-5.571244151	-6.935417351	8.207302215
C	-5.583651977	-8.308013581	7.589388381
H	-4.570779825	-8.614091085	7.332921206
H	-6.215971593	-8.343680267	6.705984360
H	-5.965999217	-8.999647390	8.335611518

Zeonex + L

C	-2.086621703	0.924317589	0.097729394
C	-0.694909602	0.833877470	-0.545326633
C	-1.380535582	2.884219955	-1.110682544
C	-2.569448231	2.330153441	-0.314459383
H	-2.752699034	0.136314328	-0.253173744
H	-2.011029619	0.838333397	1.182499703
H	-3.478312563	2.296421502	-0.915032967
H	-2.769558417	2.950494522	0.559823454
C	-0.202850146	2.267628024	-0.360372552
H	0.760048895	2.468715377	-0.817905618
H	-0.169220288	2.565049654	0.687364511
C	-0.911393370	0.716077079	-2.064528259
C	-1.332266698	2.153797211	-2.459131937
H	-2.315804128	2.171099660	-2.931384842
H	-0.078036103	0.050319439	-0.112454723
H	-1.385226633	3.969816244	-1.203062074
C	0.294254954	0.365032396	-2.957123025
C	0.897905658	1.708349629	-3.376036866
C	-0.273811089	2.697940732	-3.438701898
H	1.638364528	2.029877455	-2.642632426
H	1.408805442	1.628531700	-4.336035977
H	0.056112436	3.692361018	-3.122402754
H	-1.710397753	-0.004903942	-2.253721595
C	-0.827766406	2.823029245	-4.862657080
H	0.002970450	2.990240606	-5.552482638
H	-1.310047937	1.885318009	-5.146698506
C	-1.814786781	3.975355575	-5.009145485
H	-2.188112596	4.028506750	-6.029507687
H	-2.664521413	3.854541422	-4.341531532
H	-1.330151560	4.920318489	-4.772073535
C	3.693536137	-5.002447285	-5.526124681
C	2.309194982	-5.397194199	-4.993276642
C	2.088071713	-3.215633013	-5.422393057
C	3.543743463	-3.495257205	-5.814708073
H	4.485876265	-5.214782487	-4.807856954
H	3.907929720	-5.552791974	-6.440532662
H	4.244398093	-2.887850825	-5.240849335
H	3.701064149	-3.285550548	-6.872568526
C	1.414084023	-4.513963630	-5.858705151
H	0.358376325	-4.567365540	-5.609222382
H	1.546748650	-4.712565207	-6.921124061
C	2.171968162	-4.786888933	-3.588184126
C	1.993978273	-3.278576804	-3.891236009
H	2.792402176	-2.682631393	-3.445342268
H	2.130974500	-6.468033740	-5.047372649

H	1.689985255	-2.293176003	-5.845151976
C	0.962455662	-5.200680921	-2.725387207
C	-0.112398094	-4.141269206	-2.988324138
C	0.636666434	-2.841648968	-3.310129197
H	-0.732370675	-4.444736302	-3.833306793
H	-0.772815052	-4.023721928	-2.128001862
H	0.077507107	-2.264469804	-4.050973398
H	3.088895648	-4.977394337	-3.025013494
C	0.811004334	-1.976383451	-2.056330163
H	-0.149823900	-1.891550741	-1.545525725
H	1.501154205	-2.484113652	-1.378295965
C	1.338479519	-0.569328817	-2.349470507
H	1.722290004	-0.126856252	-1.428063284
H	2.176581939	-0.635758731	-3.047090645
H	-0.105891651	-0.111930936	-3.857779992
H	1.267452859	-5.120806666	-1.676200592
C	-1.506904480	-10.790867689	0.757726612
C	0.025577341	-10.698936221	0.820782818
C	-0.602778517	-8.574147465	0.535818908
C	-1.940568829	-9.323126508	0.567930504
H	-1.844613225	-11.426276200	-0.060583441
H	-1.902746661	-11.201867691	1.687482070
H	-2.513421483	-9.178373917	-0.347312635
H	-2.550551500	-8.981134090	1.405286421
C	0.206634565	-9.372343142	1.554868954
H	1.239426675	-9.052025009	1.642732242
H	-0.251141997	-9.375595664	2.543864348
C	0.517196948	-10.388702170	-0.603017953
C	0.089647110	-8.912481405	-0.791875218
H	-0.617650549	-8.807457242	-1.615277636
H	0.482057940	-11.573702397	1.277855228
H	-0.696246846	-7.504003679	0.719634498
C	2.033818254	-10.437599860	-0.882517911
C	2.532634727	-9.001235193	-0.695822665
C	1.360067771	-8.094180555	-1.087919261
H	2.820269392	-8.835997206	0.343296733
H	3.413295991	-8.806629613	-1.308914720
H	1.372352542	-7.177726025	-0.491067558
H	0.002807780	-11.045750064	-1.307625076
C	1.434856565	-7.707722349	-2.569195275
H	2.442548932	-7.360946750	-2.800931997
H	1.250039639	-8.601306801	-3.167845180
C	0.435819431	-6.614561452	-2.960119692
H	0.169775799	-6.722319896	-4.012623784
H	-0.480934135	-6.737735646	-2.376776602
C	7.281044058	-15.022635887	-1.719806911
C	5.940286695	-15.554908043	-2.246573583
C	5.706293377	-13.339139863	-2.402831953
C	7.123880147	-13.493647476	-1.839410289
H	7.474422439	-15.342433654	-0.695761493
H	8.100162238	-15.380334095	-2.344097992
H	7.240691110	-12.987091903	-0.881288817
H	7.854206422	-13.078986796	-2.534980339
C	5.637296788	-14.539171888	-3.344149976
H	4.668435224	-14.680883835	-3.811392055
H	6.405176943	-14.504958892	-4.114753959
C	4.876246324	-15.250463328	-1.178532605
C	4.715547470	-13.712924942	-1.291299595
H	4.995065533	-13.208982443	-0.364051786
H	5.991604010	-16.598065040	-2.549398844
H	5.522534720	-12.360094840	-2.842376900

C	3.464223208	-15.844204258	-1.366148842
C	2.649527673	-14.758696623	-2.076702257
C	3.236483467	-13.422419301	-1.608019902
H	2.741381727	-14.865369292	-3.158129723
H	1.589069188	-14.840236177	-1.835157841
H	3.153066080	-12.673397320	-2.399992334
H	5.256892213	-15.547080351	-0.198008065
C	2.502104938	-12.894837294	-0.368696290
H	1.425384027	-12.980680100	-0.522727154
H	2.762671388	-13.525004172	0.484762238
C	2.838805346	-11.434794096	-0.050712934
H	2.669278416	-11.237789447	1.009248764
H	3.900067389	-11.259876351	-0.243677399
H	2.157602768	-10.698549219	-1.938972639
H	3.037290912	-15.973924654	-0.366275361
C	-0.365593609	-21.476755932	-0.368251729
C	0.862942730	-21.402979818	0.550217584
C	0.550045880	-19.272798542	-0.048535018
C	-0.565777986	-20.009708654	-0.800547679
H	-0.203822148	-22.141839684	-1.216156879
H	-1.231576595	-21.844427271	0.183586462
H	-0.479739754	-19.880337690	-1.879299863
H	-1.545158867	-19.638811382	-0.496430915
C	0.614549801	-20.069543244	1.252191831
H	1.417638177	-19.760213143	1.913023679
H	-0.326079580	-20.055481696	1.801848288
C	2.078094857	-21.116552363	-0.348260019
C	1.884706748	-19.625969167	-0.718109799
H	1.813995448	-19.484188830	-1.797877445
H	0.970450877	-22.277824583	1.187034887
H	0.379633059	-18.200611749	0.044230691
C	3.484687669	-21.228215341	0.270315418
C	3.836086928	-19.820587582	0.759176965
C	3.095615195	-18.846796158	-0.168652929
H	3.509291664	-19.693694089	1.792003846
H	4.913287886	-19.652817365	0.738990665
H	2.753384888	-17.980446438	0.403186102
H	2.029504536	-21.766117662	-1.225730718
C	4.000492421	-18.343657633	-1.299216957
H	4.951113856	-18.013763429	-0.876928443
H	4.210557235	-19.176164785	-1.975008076
C	3.385530142	-17.186349861	-2.091476768
H	3.880499363	-17.101731619	-3.060318763
H	2.332482170	-17.402988732	-2.286636135
C	3.635456656	-22.282743264	1.358945929
H	3.033329666	-22.034371750	2.229555049
H	4.675645582	-22.342957864	1.673285784
H	4.172471284	-21.485853860	-0.543172703
C	3.318835587	-10.959781405	-5.934599581
C	4.371118290	-11.791813190	-5.588290944
H	4.221014920	-12.565705038	-4.855668454
C	5.624958752	-11.628729639	-6.169367147
C	5.812214500	-10.623384211	-7.116063448
H	6.767486447	-10.466678101	-7.590180167
C	4.750926545	-9.811701872	-7.467284941
H	4.898598235	-9.036206759	-8.204149395
C	3.500127750	-9.961037121	-6.889470001
C	2.345924638	-9.090951263	-7.320890610
C	1.177497429	-9.232652538	-6.376014407
C	0.107545933	-8.354839160	-6.444515887
H	0.139105965	-7.543253358	-7.156251872

C	-0.988037398	-8.484224098	-5.612920464
H	-1.794402402	-7.774394363	-5.700536632
C	-1.029974103	-9.524089231	-4.685681502
C	0.035555314	-10.416688477	-4.614948053
H	0.013326312	-11.219613074	-3.897200384
C	1.134347727	-10.271813140	-5.448274052
C	1.928837926	-9.403007127	-8.737506488
C	1.447429439	-10.571436411	-9.289676594
H	1.316553461	-11.459671199	-8.690446011
C	1.138896305	-10.562407475	-10.642147496
H	0.760242155	-11.462046690	-11.103920525
C	1.307128817	-9.419213709	-11.414820638
H	1.056647971	-9.448682202	-12.464499016
C	1.790302360	-8.247136636	-10.857391074
H	1.927553446	-7.350741291	-11.441330733
C	2.097662634	-8.258943747	-9.507625566
C	2.625094239	-7.197292243	-8.622830937
O	6.578515762	-12.489947997	-5.745758872
O	2.136715672	-11.180202412	-5.294271956
O	-2.038842760	-9.749869687	-3.812809466
O	2.762062773	-7.714641134	-7.385490618
O	2.903568967	-6.060429404	-8.889900718
C	-3.123187376	-8.834982653	-3.777648189
H	-2.760933667	-7.818349717	-3.560088560
H	-3.656584155	-8.829138236	-4.740553437
C	7.908592484	-12.322435323	-6.208159701
H	7.969924889	-12.512519327	-7.291042983
H	8.263867428	-11.301343388	-6.001693201
C	8.757696496	-13.269308133	-5.508125010
C	9.436630490	-14.053555127	-4.912608790
C	-4.037094072	-9.250857396	-2.729927099
C	-4.771342822	-9.608292501	-1.855907306
H	3.332643042	-23.260974022	0.991482716
H	10.048406331	-14.736645566	-4.402968637
H	-5.426682885	-9.912410885	-1.095355288

Zeonex + 1

C	-2.054802057	0.914604750	0.116638422
C	-0.669710786	0.814271115	-0.539019449
C	-1.350185331	2.865684348	-1.106649832
C	-2.534726496	2.320851249	-0.297510846
H	-2.727770722	0.128161029	-0.224338519
H	-1.969414645	0.833362957	1.201170889
H	-3.448874340	2.288577306	-0.890122290
H	-2.724451608	2.946104907	0.575746696
C	-0.168781066	2.246275433	-0.364549062
H	0.790803316	2.440519156	-0.831821351
H	-0.123944161	2.548085058	0.681643495
C	-0.901253457	0.691296131	-2.055569061
C	-1.317579855	2.129619344	-2.452471633
H	-2.305138985	2.150388006	-2.916093289
H	-0.052817224	0.029120170	-0.109125141
H	-1.350411523	3.951004288	-1.203263971
C	0.294315685	0.329906691	-2.957397260
C	0.901959260	1.668153734	-3.386585140
C	-0.264923348	2.663828150	-3.443737513
H	1.650247610	1.988622031	-2.660596892
H	1.404546974	1.581734074	-4.350347133
H	0.072811009	3.658200000	-3.135270959
H	-1.705747255	-0.026126635	-2.234223587

C	-0.830662952	2.784253747	-4.863447548
H	-0.005265443	2.944016777	-5.561367488
H	-1.319639520	1.847369968	-5.138302421
C	-1.813735765	3.940312831	-5.007370024
H	-2.196497353	3.988978280	-6.024439028
H	-2.657696944	3.827357062	-4.331073362
H	-1.322628924	4.884508627	-4.780596939
C	3.664042422	-5.072621612	-5.509029999
C	2.272699682	-5.452178562	-4.983133386
C	2.078661173	-3.267637207	-5.410722415
C	3.532110828	-3.563977033	-5.798551507
H	4.450330410	-5.293422996	-4.786535886
H	3.877631414	-5.625182097	-6.422013695
H	4.238376223	-2.963829997	-5.223496761
H	3.694050331	-3.357406637	-6.856361780
C	1.391275996	-4.557493025	-5.850617514
H	0.334233302	-4.600009433	-5.605009413
H	1.525629757	-4.756585970	-6.912567442
C	2.137940247	-4.841959358	-3.577684357
C	1.977867659	-3.331431948	-3.880192892
H	2.780767544	-2.744477932	-3.429837595
H	2.080887661	-6.520362803	-5.040471953
H	1.692849064	-2.339813996	-5.833710408
C	0.922681249	-5.241968083	-2.716486116
C	-0.140446334	-4.172123046	-2.983704710
C	0.622213268	-2.880495663	-3.305774674
H	-0.760990831	-4.471239527	-3.829504663
H	-0.802261607	-4.047397133	-2.125398485
H	0.071570009	-2.300345805	-4.050768125
H	3.051551872	-5.043218236	-3.012593066
C	0.800867043	-2.011955793	-2.054829874
H	-0.159356003	-1.919258581	-1.544175989
H	1.487947658	-2.521106078	-1.374708747
C	1.337113754	-0.609647818	-2.355468878
H	1.732598088	-0.166780667	-1.439042302
H	2.168357809	-0.685153613	-3.060298928
H	-0.116776690	-0.148008415	-3.852490795
H	1.226988316	-5.161396523	-1.666853855
C	-1.552350482	-10.754844286	0.815730776
C	-0.015070119	-10.738562421	0.835565909
C	-0.551285543	-8.577529687	0.659444610
C	-1.921711414	-9.261156385	0.718517607
H	-1.945509805	-11.323313384	-0.026385275
H	-1.940214215	-11.201817391	1.733899972
H	-2.525823463	-9.045282769	-0.161330209
H	-2.479003489	-8.931865529	1.599048922
C	0.253652186	-9.454180165	1.616227547
H	1.303060531	-9.187363802	1.680539779
H	-0.169810050	-9.476683793	2.621043988
C	0.456947317	-10.389862381	-0.585060336
C	0.060648305	-8.898152414	-0.712447467
H	-0.690927118	-8.753887995	-1.488945559
H	0.409398365	-11.654653440	1.241087452
H	-0.583746146	-7.512164959	0.886175761
C	1.969917523	-10.462112780	-0.888685738
C	2.498076106	-9.036017602	-0.699056753
C	1.333659362	-8.108255050	-1.062456375
H	2.809056682	-8.882505633	0.335379663
H	3.369709388	-8.852566866	-1.328149986
H	1.384709747	-7.183258236	-0.480908188
H	-0.085302162	-11.008503356	-1.302925468

C	1.370039762	-7.752620071	-2.552915557
H	2.377513018	-7.432017889	-2.821345917
H	1.149726682	-8.655328261	-3.124190924
C	0.382465866	-6.651369263	-2.946916769
H	0.118576730	-6.762265492	-3.999960066
H	-0.537516905	-6.764355901	-2.367118960
C	7.314931873	-15.027747808	-1.417729989
C	6.032897901	-15.564451166	-2.070170029
C	5.812681083	-13.349204559	-2.254358435
C	7.179523419	-13.500676507	-1.575588466
H	7.401307752	-15.334125376	-0.372780461
H	8.196478575	-15.392202760	-1.956441285
H	7.228381362	-12.976069796	-0.620887392
H	7.964283019	-13.109347614	-2.224029721
C	5.831016776	-14.551555072	-3.193805495
H	4.910491551	-14.693035897	-3.749298354
H	6.666544562	-14.515109764	-3.891257391
C	4.881037132	-15.258202577	-1.097344295
C	4.735465147	-13.720055107	-1.223841868
H	4.945503634	-13.216937328	-0.277646621
H	6.113106102	-16.608427231	-2.366901645
H	5.666243011	-12.374974828	-2.716199264
C	3.488277859	-15.848080261	-1.400168188
C	2.742840519	-14.760655338	-2.179936305
C	3.283404659	-13.429146463	-1.646690486
H	2.939105226	-14.859279764	-3.247697608
H	1.664261087	-14.843042884	-2.041036083
H	3.253870694	-12.664948898	-2.427381567
H	5.176006418	-15.555708139	-0.087026487
C	2.457585690	-12.929400414	-0.453467024
H	1.395196898	-13.006527121	-0.690161481
H	2.651358550	-13.581816201	0.401330939
C	2.774546723	-11.477774185	-0.078829164
H	2.593102054	-11.317789779	0.985293387
H	3.835732945	-11.285746413	-0.254833335
H	2.075184589	-10.717889987	-1.947985362
H	2.977926118	-15.968788584	-0.438742878
C	-0.350638168	-21.450699150	-0.413341726
C	0.860096110	-21.373115726	0.528072814
C	0.561235754	-19.245859980	-0.088034960
C	-0.541957367	-19.985483419	-0.855851939
H	-0.172682370	-22.119447422	-1.255007130
H	-1.227110145	-21.816079895	0.123314834
H	-0.437481100	-19.861015079	-1.933376920
H	-1.526074071	-19.612480616	-0.570244268
C	0.599656880	-20.035733812	1.218018017
H	1.389370857	-19.723698862	1.893515420
H	-0.351752469	-20.018170467	1.748815284
C	2.092525186	-21.092853734	-0.348368117
C	1.906893035	-19.604654355	-0.732327130
H	1.854144209	-19.469668637	-1.813837713
H	0.954494672	-22.244693780	1.171568769
H	0.390859161	-18.172998629	-0.004572659
C	3.486124453	-21.200166101	0.299518615
C	3.825926772	-19.789231541	0.786642197
C	3.109779549	-18.823716500	-0.168895494
H	3.472722847	-19.653895435	1.809674169
H	4.903750551	-19.622448816	0.793205209
H	2.761090793	-17.947728693	0.383742600
H	2.061486008	-21.748651076	-1.221886225
C	4.044956858	-18.343407059	-1.284897514

H	4.985535937	-18.013466880	-0.840056898
H	4.268541209	-19.188909315	-1.940164597
C	3.466768784	-17.195890419	-2.118422542
H	4.021125434	-17.112626631	-3.054849001
H	2.430070230	-17.423967699	-2.377182890
C	3.615795545	-22.248485470	1.396829542
H	2.997530503	-21.995013705	2.254570693
H	4.649978751	-22.307854226	1.730917438
H	4.190839670	-21.461526927	-0.498180529
C	3.310479436	-10.896364346	-5.976246136
C	4.368466166	-11.716306436	-5.621271330
H	4.243507237	-12.441817848	-4.835189928
C	5.600805614	-11.608618204	-6.261998705
C	5.754905150	-10.665971150	-7.279292949
H	6.691242774	-10.556166564	-7.802122497
C	4.690789976	-9.859732697	-7.630388860
H	4.816210896	-9.130097030	-8.417316351
C	3.463017808	-9.954908171	-6.992408255
C	2.315036035	-9.070856246	-7.411805479
C	1.145292357	-9.209323508	-6.468646654
C	0.051668032	-8.363597825	-6.578858901
H	0.061594246	-7.590150631	-7.333125826
C	-1.038483999	-8.475401545	-5.739221033
H	-1.864674972	-7.793623520	-5.860510430
C	-1.055415852	-9.465112400	-4.755036262
C	0.033225892	-10.328050336	-4.647646143
H	0.030630042	-11.095568899	-3.891386724
C	1.127408331	-10.199375239	-5.488419281
C	1.899978004	-9.343199951	-8.835646816
C	1.406720488	-10.492062063	-9.418213160
H	1.262626377	-11.392306443	-8.840321764
C	1.104167896	-10.447934311	-10.771144785
H	0.716821237	-11.332075835	-11.255501301
C	1.289056362	-9.289368492	-11.516788717
H	1.042822315	-9.291284062	-12.568063508
C	1.783746342	-8.136898884	-10.929808897
H	1.934630390	-7.228568903	-11.491820470
C	2.085444818	-8.184283251	-9.579628502
C	2.622781957	-7.149534816	-8.669355304
O	6.554893802	-12.449018959	-5.825190920
O	2.150142474	-11.075030859	-5.284989268
O	-2.044138490	-9.663966550	-3.865589984
O	2.749071064	-7.694343777	-7.445430613
O	2.914843089	-6.009458332	-8.912035559
C	-3.153968690	-8.768729514	-3.848014965
H	-2.798364212	-7.730015159	-3.763165248
H	-3.737991538	-8.872292252	-4.775978797
C	7.873237100	-12.355737386	-6.359987669
H	7.871720345	-12.634512713	-7.425529926
H	8.251662442	-11.326796622	-6.260011384
C	8.711934510	-13.268196545	-5.596734787
C	9.371174640	-14.029721977	-4.936669808
C	-3.975644143	-9.097632591	-2.692563059
C	-4.632504944	-9.386208630	-1.725086576
Au	10.239887545	-15.256809862	-3.695685797
P	10.992557498	-16.652431687	-2.076669220
N	12.764394191	-18.357413188	-0.800442076
N	10.406007850	-18.862769003	-0.517568391
N	11.390355473	-16.970749859	0.643632042
C	12.629754670	-17.553566532	-1.995063393
H	12.723028852	-18.199991806	-2.876557677

H	13.440754139	-16.815417266	-2.025734239
C	9.932621784	-18.130615426	-1.671260661
H	8.908799785	-17.784617952	-1.483909639
H	9.913115636	-18.800334030	-2.540258506
C	11.059280366	-15.968276607	-0.344980995
H	11.808481090	-15.167405973	-0.310799944
H	10.082537425	-15.531007366	-0.105791254
C	10.403654625	-18.037923209	0.679824744
H	9.409559541	-17.602494218	0.811997747
H	10.629953643	-18.680986716	1.535837015
C	12.703177173	-17.548062416	0.406277953
H	13.441374897	-16.744446488	0.337459624
H	12.951746604	-18.189316390	1.257394930
C	11.744285924	-19.390967710	-0.726335739
H	11.758683502	-19.976230432	-1.649833925
H	11.986283066	-20.046511988	0.115723452
Au	-5.512717285	-9.842350968	-0.045898151
P	-6.316013909	-10.346539535	2.009158335
N	-5.626726431	-11.337056113	4.500656895
N	-7.931044886	-11.684467844	3.816269759
N	-7.184126662	-9.473883397	4.489242375
C	-5.086685600	-11.115844593	3.177207811
H	-4.753518947	-12.072890433	2.757068681
H	-4.214199844	-10.454599111	3.247654846
C	-7.721132952	-11.516899316	2.395298710
H	-8.640605029	-11.130552258	1.938140935
H	-7.500035030	-12.492872866	1.945585866
C	-6.864166173	-8.988678062	3.164681462
H	-6.059409830	-8.246337709	3.236342058
H	-7.746365186	-8.494313873	2.739272935
C	-8.279587899	-10.429530842	4.463061919
H	-9.132708788	-9.983408386	3.944792123
H	-8.564186311	-10.648113042	5.496765648
C	-6.034061944	-10.091340195	5.130111238
H	-5.195162809	-9.390328083	5.114006041
H	-6.299855812	-10.306681045	6.169522337
C	-6.761418104	-12.245615302	4.473754673
H	-6.470519209	-13.167519851	3.963009898
H	-7.033531585	-12.480198665	5.507346339
H	3.319026270	-23.228453784	1.029139241

Zeonex + 2

C	-2.212513587	1.027065448	-0.000021793
C	-0.802592300	0.938183835	-0.602113233
C	-1.467537061	2.993211635	-1.175035444
C	-2.681570532	2.435082346	-0.420533597
H	-2.868460633	0.241053043	-0.373693816
H	-2.169029119	0.936770721	1.086301415
H	-3.570205484	2.404321166	-1.050835502
H	-2.910388024	3.051092859	0.449873636
C	-0.314138170	2.369905583	-0.393211549
H	0.662291970	2.572050604	-0.820605833
H	-0.311403859	2.660809866	0.656985752
C	-0.974600482	0.830322092	-2.127823902
C	-1.376544118	2.272276573	-2.526225181
H	-2.343028734	2.296646010	-3.032171135
H	-0.199885338	0.150801266	-0.156472605
H	-1.468381814	4.079497912	-1.259800215
C	0.254763965	0.478165393	-2.986607688
C	0.879536556	1.820620625	-3.376237068
C	-0.282471936	2.819977459	-3.464028358

H	1.601091003	2.130275946	-2.619110986
H	1.417159382	1.745041495	-4.321819894
H	0.044552772	3.807562997	-3.123859993
H	-1.771084976	0.114451470	-2.344592535
C	-0.788783625	2.968922120	-4.903236914
H	0.065068222	3.140415090	-5.563108944
H	-1.267383218	2.038712323	-5.216438639
C	-1.763147146	4.130046988	-5.064663416
H	-2.101474707	4.201702626	-6.096026359
H	-2.635692454	4.004489681	-4.428026884
H	-1.280938102	5.067954896	-4.796393527
C	3.663102703	-4.899871133	-5.516982397
C	2.260096395	-5.279259502	-5.023141823
C	2.084568819	-3.088301582	-5.425318029
C	3.545527961	-3.385723916	-5.783324169
H	4.435063333	-5.137959169	-4.784674833
H	3.889166420	-5.438244701	-6.435449223
H	4.240181456	-2.799557790	-5.180376699
H	3.734835720	-3.160827605	-6.832808648
C	1.401296913	-4.369066034	-5.897329282
H	0.338958392	-4.409320783	-5.675312197
H	1.557669281	-4.555560881	-6.958642465
C	2.100903917	-4.686511166	-3.612733109
C	1.948336885	-3.171911234	-3.898562053
H	2.740369753	-2.591314721	-3.421571367
H	2.064771251	-6.345726718	-5.097910535
H	1.712465701	-2.153184386	-5.844469834
C	0.870015261	-5.093997314	-2.778242797
C	-0.189060203	-4.023820883	-3.059366181
C	0.579196247	-2.728147113	-3.350723382
H	-0.789525310	-4.315739308	-3.921969803
H	-0.870442500	-3.908495401	-2.215127653
H	0.045106979	-2.140958087	-4.102151099
H	3.003812187	-4.896961270	-3.033990740
C	0.726632899	-1.873024040	-2.086538086
H	-0.246776062	-1.784016098	-1.600716783
H	1.394490718	-2.391017955	-1.393980922
C	1.273645468	-0.468581202	-2.355624975
H	1.635437896	-0.034631053	-1.421135830
H	2.130222873	-0.538285947	-3.030045710
H	-0.121538369	0.011046196	-3.902446559
H	1.153817794	-5.017897814	-1.722628073
C	-1.633719377	-10.487303443	0.841419260
C	-0.096443112	-10.491704395	0.855340224
C	-0.604546210	-8.330178315	0.614693137
C	-1.983750008	-8.992512895	0.701721011
H	-2.038505982	-11.075199516	0.018303317
H	-2.022264979	-10.900595154	1.775349587
H	-2.589602196	-8.796303083	-0.181389409
H	-2.530670121	-8.630422487	1.575433641
C	0.193455579	-9.187345943	1.594521983
H	1.246690221	-8.933063103	1.643907185
H	-0.224492785	-9.171960597	2.601762595
C	0.374939689	-10.193812878	-0.576860176
C	-0.001471414	-8.701325583	-0.748270994
H	-0.752737338	-8.571338746	-1.527445224
H	0.316968369	-11.400803578	1.287042308
H	-0.621704822	-7.258001027	0.808205648
C	1.885843841	-10.297217329	-0.881220313
C	2.434321755	-8.872260006	-0.744432184
C	1.280308638	-7.937002355	-1.121913942

H	2.760324568	-8.690720429	0.280898308
H	3.300652697	-8.721522706	-1.389238079
H	1.347207396	-7.002457684	-0.557634421
H	-0.178335686	-10.826000912	-1.274169579
C	1.313468798	-7.607091179	-2.618245855
H	2.322760521	-7.301126507	-2.896824521
H	1.081016978	-8.516653208	-3.173702616
C	0.335588747	-6.502579728	-3.026215349
H	0.093424808	-6.608876275	-4.084875876
H	-0.596116811	-6.617792635	-2.465731734
C	7.297913068	-14.720865329	-1.357393581
C	6.021160810	-15.304285323	-1.978206800
C	5.712574211	-13.100382696	-2.156899269
C	7.097662884	-13.200347245	-1.506226325
H	7.417731049	-15.028988850	-0.317470609
H	8.179845528	-15.049077073	-1.918805219
H	7.143829703	-12.677114430	-0.550679163
H	7.853223702	-12.775238625	-2.167733462
C	5.751509033	-14.302038624	-3.096619385
H	4.819628868	-14.480009142	-3.622428149
H	6.563878766	-14.239523677	-3.819277772
C	4.888291041	-15.040306642	-0.971700792
C	4.667612146	-13.513076624	-1.109874431
H	4.860295528	-12.993313796	-0.169275312
H	6.127768403	-16.344255485	-2.278464839
H	5.519409474	-12.131164516	-2.613212460
C	3.517626355	-15.699408122	-1.220973431
C	2.698283416	-14.663911700	-1.998151458
C	3.202039259	-13.291869406	-1.531067777
H	2.855591829	-14.788343495	-3.069994119
H	1.630204773	-14.784473608	-1.813269308
H	3.143859118	-12.569156299	-2.349243981
H	5.231161306	-15.314324849	0.028933339
C	2.369150237	-12.754374810	-0.359946818
H	1.307920081	-12.843045970	-0.598693382
H	2.562276373	-13.376469901	0.517324313
C	2.682118649	-11.289642740	-0.034362440
H	2.495834463	-11.093978727	1.022885527
H	3.744100513	-11.103713926	-0.210765077
H	1.985240042	-10.593559982	-1.930328743
H	3.044285716	-15.829548857	-0.241999977
C	0.388396048	-21.863910451	-0.245539568
C	1.556882213	-21.604079511	0.717190813
C	0.922150217	-19.546242010	0.121447701
C	-0.043748011	-20.443259412	-0.663218012
H	0.688785292	-22.473653488	-1.097396704
H	-0.420305604	-22.384867724	0.268555470
H	0.037494382	-20.284016948	-1.738238524
H	-1.076027993	-20.244125904	-0.372947804
C	1.074470363	-20.336392717	1.418767116
H	1.795229143	-19.909318836	2.107925845
H	0.126627142	-20.480771663	1.936786211
C	2.740967560	-21.114584149	-0.132976492
C	2.310205499	-19.678402249	-0.520134327
H	2.231936526	-19.555310668	-1.602007556
H	1.781087199	-22.458549956	1.351715550
H	0.582539476	-18.515284932	0.215626537
C	4.118213064	-20.983564558	0.548983017
C	4.207630606	-19.532076563	1.026758415
C	3.373826719	-18.712152373	0.033762795
H	3.804845297	-19.445547510	2.036552813

H	5.237894299	-19.180062680	1.056572759
H	2.894602135	-17.876526277	0.549614698
H	2.842146271	-21.763573043	-1.006731827
C	4.250471343	-18.144597017	-1.088940621
H	5.158998182	-17.731654014	-0.650307560
H	4.546903995	-18.965983023	-1.746913495
C	3.550340655	-17.060287045	-1.914143355
H	4.046753227	-16.956719290	-2.880985707
H	2.520143021	-17.367048152	-2.113201397
C	4.389749163	-21.990242283	1.659652803
H	3.714155430	-21.838406873	2.497742462
H	5.409177859	-21.875854136	2.023526381
H	4.876900711	-21.130210687	-0.229041691
C	3.355858010	-10.815886329	-5.954087668
C	4.408949197	-11.641146629	-5.599331727
H	4.272763392	-12.383217845	-4.831192407
C	5.649426350	-11.521909737	-6.221409389
C	5.821316416	-10.554287673	-7.211236626
H	6.768120459	-10.430096811	-7.711873320
C	4.761174325	-9.741673763	-7.562195342
H	4.898807164	-8.993997730	-8.329877691
C	3.521983963	-9.854557995	-6.949345090
C	2.371281999	-8.977807730	-7.378150469
C	1.190282057	-9.131880854	-6.450875758
C	0.093003987	-8.291312116	-6.563382566
H	0.109223491	-7.504798665	-7.303930684
C	-1.009892602	-8.425139294	-5.743146755
H	-1.839556476	-7.747875664	-5.866317640
C	-1.035465546	-9.431684902	-4.776777839
C	0.056866693	-10.289428892	-4.666956351
H	0.047883925	-11.069868724	-3.924121024
C	1.162326148	-10.139398487	-5.488715134
C	1.974802045	-9.255001856	-8.806593375
C	1.496846287	-10.408628512	-9.392443425
H	1.352746388	-11.308681998	-8.814278226
C	1.209471074	-10.368916287	-10.748773815
H	0.833884265	-11.256507849	-11.236035086
C	1.394704520	-9.210135418	-11.494013734
H	1.160704973	-9.215662328	-12.548062273
C	1.873852947	-8.052911861	-10.903410318
H	2.024152603	-7.144573345	-11.465510185
C	2.159957360	-8.095600208	-9.549627565
C	2.677078491	-7.055519273	-8.633603622
O	6.589418362	-12.381646963	-5.791379709
O	2.185580955	-11.013804534	-5.285983212
O	-2.035506221	-9.654090869	-3.905389724
O	2.791711384	-7.597678626	-7.407114943
O	2.963415010	-5.913634400	-8.874361584
C	-3.150331484	-8.766222394	-3.880697688
H	-2.802763707	-7.726085339	-3.781813078
H	-3.732277028	-8.862193312	-4.810592672
C	7.900352085	-12.348154757	-6.349384180
H	7.861585372	-12.547507703	-7.431473434
H	8.361961591	-11.362675176	-6.182776197
C	8.658133399	-13.386837070	-5.666175104
C	9.219641571	-14.265738022	-5.063971754
C	-3.968967120	-9.118346255	-2.729888685
C	-4.616836709	-9.432623009	-1.764499440
Au	9.846744433	-15.684223471	-3.882815154
Au	-5.477084855	-9.928409052	-0.086919429
H	4.270257734	-23.008674063	1.295072675

P	10.293107496	-17.255073597	-2.305662838
O	10.703598280	-18.444240422	2.378134136
O	7.427995119	-18.238554336	0.429845653
N	11.433110246	-19.434614052	-1.143669627
N	9.049233335	-19.217271480	-0.769221182
N	11.039314746	-17.557613987	0.349927004
C	10.849674236	-16.563096778	-0.670096621
H	11.795552781	-16.029461527	-0.866111489
H	10.100173995	-15.828023839	-0.369803290
C	8.825609769	-18.279089635	-1.844470692
H	8.005091834	-17.614902421	-1.548129334
H	8.526202831	-18.817302777	-2.758784221
C	11.488143528	-18.676944765	-2.372105451
H	11.231472530	-19.325360217	-3.217776129
H	12.506705948	-18.302989122	-2.523535803
C	11.900211081	-18.674359027	-0.013890856
H	12.900994669	-18.294666153	-0.257239805
H	11.960724501	-19.326187414	0.861631345
C	10.175327334	-20.114425831	-0.944415902
H	10.276810326	-20.747563299	-0.063988793
H	10.001280413	-20.744686826	-1.828521110
C	10.494323566	-17.530937389	1.603110599
C	9.636292421	-16.348814067	1.966159624
H	10.142845124	-15.405557983	1.777082946
H	9.402339094	-16.424010913	3.024842142
H	8.702113562	-16.386082326	1.407401883
C	8.223855421	-19.150895410	0.319710711
C	8.327324515	-20.255684258	1.336382179
H	9.236856088	-20.141284095	1.923721246
H	8.310190539	-21.235306699	0.865560402
H	7.478203696	-20.163321226	2.008968322
P	-6.248101427	-10.467763838	1.971652745
O	-9.124408174	-10.270472467	5.895261910
O	-9.080606552	-13.050878064	3.265432488
N	-5.619414277	-10.611227119	4.611822549
N	-6.991342817	-12.433152477	3.793408803
N	-7.846073013	-9.756416574	4.130264726
C	-7.625293860	-9.472941324	2.738796658
H	-7.346921454	-8.414002412	2.599704367
H	-8.525523180	-9.668466152	2.151880552
C	-6.700216350	-12.212355665	2.396193901
H	-7.580096799	-12.515544568	1.815885416
H	-5.844447029	-12.833063325	2.085421179
C	-5.022942671	-10.266035052	3.342262151
H	-4.160675226	-10.914040778	3.147892846
H	-4.669845581	-9.229450036	3.374418950
C	-6.666170377	-9.696690427	4.983482381
H	-6.248602935	-8.681804654	4.951167032
H	-7.000140988	-9.920410970	6.000068740
C	-5.959597252	-12.010014022	4.721028226
H	-6.290650386	-12.192646402	5.742382122
H	-5.042816505	-12.584621542	4.526440954
C	-9.055403271	-10.030896808	4.705255666
C	-10.268688022	-10.029954269	3.814127184
H	-10.351448303	-9.107375061	3.244661502
H	-11.143696682	-10.137447959	4.449623226
H	-10.232130887	-10.882663397	3.137556923
C	-8.224581661	-12.930914686	4.119558420
C	-8.447368745	-13.343260751	5.549912021
H	-8.571965107	-12.463664138	6.179356669
H	-7.628989838	-13.953086978	5.923614675

H	-9.370574885	-13.915819639	5.585635384
